# Supplementary material for: Transcriptome Remodeling Contributes to Epidemic Disease Caused by the Human Pathogen Streptococcus pyogenes
Source: mBio. 2016 May 31;7(3):e00403-16. doi: 10.1128/mBio.00403-16 (PMC4895104; doi:10.1128/mBio.00403-16)
Supplement: Text S1 — Supplemental Materials and Methods. Download [file mbo003162837s1.docx]

**Supplemental Materials and Methods**

**Strains**

The 1,200 GAS *emm*89 strains, 2 isolates of pharyngitis and 1,198 of invasive infections, studied are listed in Table S1. The vast majority of the strains, 1,178 were collected as part of comprehensive population-based public health surveillance of GAS invasive infections conducted in the United States, Finland, and Iceland between 1995 and 2014. Included in the study, as a sampling from additional geographic regions, are 21 strains from Canada and 1 strain from Italy. Strains were grown at 37°C with 5% CO2 on tryptic soy agar with 5% sheep blood (Becton, Dickinson) or in Todd-Hewitt broth with 2% w/v yeast extract (THY)(Difco Laboratories).

**Population genomic sequencing**

Isolation of chromosomal DNA, generation of paired-end libraries, and multiplexed sequencing were accomplished as previously described (1, 2). Briefly, DNA was extracted from overnight cell cultures using DNeasy 96 Blood & Tissue Kit (Qiagen), multiplexed libraries were generated using Nextera XT DNA Sample Preparation and Index Kits (Illumina), and multiplexed libraries (up to 96 samples per flow cell lane) were sequenced on Illumina instruments (HiSeq2500, MiSeq, NextSeq).

**Reference genome assembly and annotation**

Complete closed and annotated genome sequences were determined for 3 strains, one each representative of the 3 major phylogenetic clades identified in the *emm*89 population to serve as references in subsequent analyses. Paired-end sequence reads were *de novo* assembled using SPAdes (3) and resultant contigs were ordered relative to reference genomes using Progressive Mauve (4). Assembly gaps between the ordered contigs were bridged by PCR and closed by Sanger sequencing. Potential assembly errors in the closed genomes were identified and corrected using iCORN2 (5). Assemblies and read mappings were manually inspected/curated using Tablet (6) and Sequencher (www.genecodes.com). Non-coding RNAs were identified using tRNAscan-SE (7), Infernal (8) and R-fam (9). Gene and coding sequence features in the closed genomes were identified and annotated using RATT (10), RAST (11), BLAST (12), Batch-CD-Search (13), and Artemis (14). Protein secretion, lipid anchoring, and cell wall anchoring signal sequences were predicted using SignalP (15), LipoP (16), CW- pred (17) respectively. Genome circular atlases (Fig. S1) were generated using GenomeViz (18). The closed and annotated genome of clade 1 strain MGAS11027, clade 2 strain MGAS23530, and clade 3 strain MGAS27601 were deposited in NCBI GenBank under accession numbers CP013838, CP013839, and CP013840 respectively, and the strains were deposited in ATCC-BEI Resources under catalog numbers NR-33707, NR-33706, and NR-50285 respectively.

**Polymorphism discovery**

Sequence reads were quality filtered using Trimmomatic (19) and base calls error corrected using Musket (20). Reads were aligned to reference genomes using SMALT (www.sanger.ac.uk/resources/software/smalt), and polymorphisms between the aligned reads and the reference genomes were called using FreeBayes (21). Polymorphism calls were filtered on the basis of mapped quality (≥Q30), call consensus (≥70%), and depth of coverage (≥10) using VCFlib (github.com/ekg/vcflib#vcflib). SNPs were annotated and consequences predicted using SnpEff (22).

**Phylogenetic inference and population structure**

Concatenated total and core SNP multiple sequence alignments were generated using Prephix, Phrecon, and SNPswapper (github.com/codinghedgehog). Sequence alignments of the recombination blocks among GAS strains (Fig. S3) were generated using MAFFT (23). To constrain phylogenetic inferences to primarily vertically inherited SNPs, regions of putative horizontal transfer were predicted and polymorphisms within these regions were filtered-out using Gubbins (24) and/or BratNextGen (25). Clades of genetically related strains were defined by Bayesian clustering, using BAPs (26). Maximum- Likelihood phylogenetic inferences were made using RAxML (27) and Neighbor-Joining and Neighbor-Network inferences were made using SplitsTree (28). Genetic distances between strains (pairwise and mean) were determined using MEGA (29).

**Gene content and mobile genetic element analysis**

The known GAS pangenome core and accessory gene content were determined on the basis of 30 complete genomes of 18 *emm*-types (Table S2) using PanOCT (30) and BLAST (12) as previously described (1). Among the 53,336 CDSs of the 30 GAS genomes, PanOCT identified 3,338 ortholog clusters. Ortholog clusters were further condensed to 2,835 unique clusters on the basis of no two clusters sharing >95% amino acid identity as determined by BLAST reciprocal-best-hit. A GAS pseudo-pangenome sequence of ~3 Mbp was generated based on the 2,835 ortholog clusters by concatenating onto the *emm*89 MGAS23530 genome all accessory gene content not already represented in the MGAS23530 genome. The accessory genes were added starting with those of *emm*89 strains MGAS11027 and MGAS27061, and then the remaining 27 genomes by increasing *emm*-type (i.e. *emm*1, *emm*2, *emm*3, etc.). Sequence reads for the 1,200 strains studied were mapped to the resultant GAS-30 pangenome using SMALT and gene fragments per kilobase per million mapped reads (FPKMs) were determined using Cufflinks (31). Based on mapping of the *emm*89 reference genome sequencing reads to the GAS-30 pangenome, a FPKM value of >50 corresponded with gene presence and <50 with gene absence. Mobile genetic element content or phage-genotype was predicted on the basis of the gene content analysis. A phage was called present if a minimum of 80 percent of the respective phage’s gene content represented in the GAS-30 pangenome was determined to be present. To test for the acquisition of new/novel gene content, reads not mapping to the GAS-30 pangenome were assembled *de novo* using SPAdes and resultant contigs >100 nucleotides were queried against the NCBI nonredundant database using BLAST to determine their nature.

**Isogenic mutant strain construction**

Isogenic mutants were constructed by allelic exchange based on subclade 3D parental strain MGAS27556, which is wild type for all known GAS virulence regulators. DNA for the allelic exchange of the LiaS (K214R) variant into MGAS27556 was amplified from strain MGAS27552, a clinical isolate with a naturally occurring *liaS* A641G SNP using primers liaS(A641G)-1 (CCG CGT **GGATCC** CGA GCG CGA CCT GAA GCA ATA CCA A) and liaS(A641G)-2 (CCG CGT **GGATCC** CTT TTA TAA CCA AGA TAG CCG CAA T). The resultant 2,409 bp amplicon was cut with *Bam*HI and cloned into the *Bam*HI site of the suicide vector pBBL740 generating plasmid pBBL740-*liaS*(A641G). pBBL740- *liaS*(A641G) was electroporated into MGAS27556 and allelic exchange through single-crossover integration followed by double-crossover resolution was performed as described (2). Exchange of the liaS (A641G) SNP into MGAS27556, generating isogenic mutant strain MGAS27556 LiaS (K214R), was screened for by Sanger sequencing using primer liaS(A641G)-seq (CCA TAT CAA ATC CTA CAC CAT CAT C). Whole genome sequencing was performed on isogenic mutant strain MGAS27556 LiaS (K214R), as a final confirmation of the allelic exchange and to rule out the presence of confounding spurious spontaneous mutations.

**RNAseq transcriptome sequencing and quantitative real-time PCR expression analyses**

Whole genome transcriptional analysis was conducted for select strains using RNAseq as previously described with minor modifications (32). Strains used for RNAseq and qPCR expression analysis were selected on the basis of being genetically representative, that is the strains were not outliers in terms of core chromosomal SNPs, MGE content, or allelic variants for known virulence regulators relative to the subset they represent. Briefly, RNA from triplicate cultures grown in THY was stabilized with RNAprotect (Qiagen), pelleted, flash frozen, and stored at -80°C. Total RNA was isolated from cells mechanically disrupted using Lysing Matrix B on a FastPrep96 (MP Bio) using RNeasy Mini kits (Qiagen). Contaminating DNA was enzymatically degraded using Turbo DNA-free kits (ThermoFisher Scientific), and mRNA was enriched by depletion of rRNA using Ribo-Zero rRNA (Bacteria) removal kits (Epicentre). RNA quality was assessed using RNA 6000 Nano kit on Agilent 2100 Bioanalyzer (Agilent Technologies) and quantified by fluorometry using Qubit RNA BR assay kit (ThermoFisher Scientific). Multiplexed directional cDNA libraries were generated from the resultant RNA using ScriptSeq v2 RNA-Seq Library Preparation and ScriptSeq Index PCR primers kits (Epicentre). Library quality was assessed using High Sensitivity DNA kits with an Agilent 2100 Bioanalyzer and quantified using Qubit dsDNA HS kits (ThermoFisher Scientific). Multiplexed libraries were single-end 50 bp sequenced to high depth (~ 10 million reads/sample) on an Illumina HiSeq2500 instrument.

Sample strain RNAseq reads were mapped to the genome of the most closely genetically related *emm*89 reference strain (*e.x*. clade 3 strains were mapped to the reference strain MGAS27061 genome) and raw read counts per gene were determined using EDGE-pro v1.3.1 (33). In this investigation the use of multiple reference sequences was necessary, as the use of a single common reference did not permit accurate quantitative read mapping to the divergent sequences in the regions of HGT. Prior to assessment of differential expression, reads corresponding to rRNA and tRNA were excluded from the input data. To get accurate read mapping (*i.e.* accurate RNAseq mapped read counts) and compensate for the difference in gene content within the RB15 HGT region of subclade 3D strains, a hybrid reference genome sequence was generated that was a composite of the MGAS27061 genome and the MGAS5005 RB15 region. RNAseq data normalization and determination of genes significantly differentially expressed with Benjamini-Hochberg correction at a minimum 1.5-fold change was conducted using DESeq2 v1.6.3 running in RStudio v3.1.3 (34). RNAseq data was deposited in the NCBI GEO database under accession number GSE76816.

Quantitative real-time PCR (qPCR) was used to assess the expression of *nga*, *slo*, and *hasA* in a larger sampling of 11 genetically representative strains selected to encompass the most abundant subclades of the major population. The subclades represented by these 11 isolates encompass 1120 of the 1193 strains (94%) of the major *emm*89 population. The 11 strains were also selected to include collection from disparate geographic locations of the United States, Finland and Iceland. Measurements were done in triplicate, and the significance of differences in expression strain-to-strain, were assessed by one-way ANOVA. The primers, probes and protocol used in the RT-PCR analysis have been previously described (35).

**Mouse and non-human primate models of infection**

The capacity of strains representing the three primary *emm*89 clades to cause deep tissue disease was assessed in mouse and non-human primate models of necrotizing fasciitis as previously described (36, 37). The representative strains used were selected in part because they are genetically wild-type (*i.e.* have the most commonly occurring normally functioning allele) for all major virulence regulators such as *mga*, *covRS*, and *ropB*. Stocks of each strain were prepared in advance at known concentrations and stored frozen at -80°C. Inoculums were prepared by diluting frozen stocks in PBS to the desired dosages. Dilution plating and CFU enumeration confirmed doses administered. For survival experiments female CD1 outbred immunocompetent 5-to-6 week old mice were randomly assigned to treatment groups, 25 mice per strain, and inoculated at a uniform depth in the right hind limb with 2.5×108 CFU in 100ul of PBS. Mice were monitored and animals displaying distress using predefined criteria for near-mortality were euthanized with isofluorane. NF in the mouse model was evaluated using 5 mice per strain treatment group. Inoculation site lesions were excised, visually inspected, fixed in 10% phosphate buffered formalin, decalcified and embedded in paraffin using automated standard instruments. A pathologist blinded to the strain treatment groups scored the histopathology as previously described (37). NF in the non-human primate model was evaluated using 3 monkeys per strain treatment group. Adult cynomolgus macaques (*Macaca fasicularis*) (Charles River BRF) were anesthetized and inoculated intramuscularly in the anterior thigh to a uniform depth with 1x109 CFU/kg body mass. The animals were observed continuously for ~24 h, sacrificed, necropsied, and lesion volumes, and CFUs/g muscle were determined as previously described (37). All animal experiments were approved by the Institutional Animal Care and Use Committee of The Houston Methodist Research Institute.

**SI References**

1. **Nasser W, Beres SB, Olsen RJ, Dean MA, Rice KA, Long SW, Kristinsson KG, Gottfredsson M, Vuopio J, Raisanen K, Caugant DA, Steinbakk M, Low DE, McGeer A, Darenberg J, Henriques-Normark B, Van Beneden CA, Hoffmann S, Musser JM.** 2014. Evolutionary pathway to increased virulence and epidemic group A Streptococcus disease derived from 3,615 genome sequences. Proc Natl Acad Sci U S A **111:**E1768-1776.

2. **Zhu L, Olsen RJ, Nasser W, Beres SB, Vuopio J, Kristinsson KG, Gottfredsson M, Porter AR, DeLeo FR, Musser JM.** 2015. A molecular trigger for intercontinental epidemics of group A Streptococcus. J Clin Invest **125:**3545-3559.

3. **Bankevich A, Nurk S, Antipov D, Gurevich AA, Dvorkin M, Kulikov AS, Lesin VM, Nikolenko SI, Pham S, Prjibelski AD, Pyshkin AV, Sirotkin AV, Vyahhi N, Tesler G, Alekseyev MA, Pevzner PA.** 2012. SPAdes: a new genome assembly algorithm and its applications to single-cell sequencing. J Comput Biol **19:**455-477.

4. **Darling AE, Mau B, Perna NT.** 2010. progressiveMauve: multiple genome alignment with gene gain, loss and rearrangement. PLoS One **5:**e11147.

5. **Otto TD, Sanders M, Berriman M, Newbold C.** 2010. Iterative Correction of Reference Nucleotides (iCORN) using second generation sequencing technology. Bioinformatics **26:**1704-1707.

6. **Milne I, Bayer M, Cardle L, Shaw P, Stephen G, Wright F, Marshall D.** 2010. Tablet--next generation sequence assembly visualization. Bioinformatics **26:**401-402.

7. **Lowe TM, Eddy SR.** 1997. tRNAscan-SE: a program for improved detection of transfer RNA genes in genomic sequence. Nucleic Acids Res **25:**955-964.

8. **Nawrocki EP, Eddy SR.** 2013. Infernal 1.1: 100-fold faster RNA homology searches. Bioinformatics **29:**2933-2935.

9. **Nawrocki EP, Burge SW, Bateman A, Daub J, Eberhardt RY, Eddy SR, Floden EW, Gardner PP, Jones TA, Tate J, Finn RD.** 2015. Rfam 12.0: updates to the RNA families database. Nucleic Acids Res **43:**D130-137.

10. **Otto TD, Dillon GP, Degrave WS, Berriman M.** 2011. RATT: Rapid Annotation Transfer Tool. Nucleic Acids Res **39:**e57.

11. **Overbeek R, Olson R, Pusch GD, Olsen GJ, Davis JJ, Disz T, Edwards RA, Gerdes S, Parrello B, Shukla M, Vonstein V, Wattam AR, Xia F, Stevens R.** 2014. The SEED and the Rapid Annotation of microbial genomes using Subsystems Technology (RAST). Nucleic Acids Res **42:**D206-214.

12. **Camacho C, Coulouris G, Avagyan V, Ma N, Papadopoulos J, Bealer K, Madden TL.** 2009. BLAST+: architecture and applications. BMC Bioinformatics **10:**421.

13. **Marchler-Bauer A, Lu S, Anderson JB, Chitsaz F, Derbyshire MK, DeWeese-Scott C, Fong JH, Geer LY, Geer RC, Gonzales NR, Gwadz M, Hurwitz DI, Jackson JD, Ke Z, Lanczycki CJ, Lu F, Marchler GH, Mullokandov M, Omelchenko MV, Robertson CL, Song JS, Thanki N, Yamashita RA, Zhang D, Zhang N, Zheng C, Bryant SH.** 2011. CDD: a Conserved Domain Database for the functional annotation of proteins. Nucleic Acids Res **39:**D225-229.

14. **Carver T, Harris SR, Berriman M, Parkhill J, McQuillan JA.** 2012. Artemis: an integrated platform for visualization and analysis of high-throughput sequence-based experimental data. Bioinformatics **28:**464-469.

15. **Petersen TN, Brunak S, von Heijne G, Nielsen H.** 2011. SignalP 4.0: discriminating signal peptides from transmembrane regions. Nat Methods **8:**785-786.

16. **Bagos PG, Tsirigos KD, Liakopoulos TD, Hamodrakas SJ.** 2008. Prediction of lipoprotein signal peptides in Gram-positive bacteria with a Hidden Markov Model. J Proteome Res **7:**5082-5093.

17. **Litou ZI, Bagos PG, Tsirigos KD, Liakopoulos TD, Hamodrakas SJ.** 2008. Prediction of cell wall sorting signals in gram-positive bacteria with a hidden markov model: application to complete genomes. J Bioinform Comput Biol **6:**387-401.

18. **Ghai R, Hain T, Chakraborty T.** 2004. GenomeViz: visualizing microbial genomes. BMC Bioinformatics **5:**198.

19. **Bolger AM, Lohse M, Usadel B.** 2014. Trimmomatic: a flexible trimmer for Illumina sequence data. Bioinformatics **30:**2114-2120.

20. **Liu Y, Schroder J, Schmidt B.** 2013. Musket: a multistage k-mer spectrum-based error corrector for Illumina sequence data. Bioinformatics **29:**308-315.

21. **Garrison E, Gabor M.** 2012. Haplotype-based variant detection from short-read sequencing.

22. **Cingolani P, Platts A, Wang le L, Coon M, Nguyen T, Wang L, Land SJ, Lu X, Ruden DM.** 2012. A program for annotating and predicting the effects of single nucleotide polymorphisms, SnpEff: SNPs in the genome of Drosophila melanogaster strain w1118; iso-2; iso-3. Fly (Austin) **6:**80-92.

23. **Katoh K, Standley DM.** 2013. MAFFT multiple sequence alignment software version 7: improvements in performance and usability. Mol Biol Evol **30:**772-780.

24. **Croucher NJ, Page AJ, Connor TR, Delaney AJ, Keane JA, Bentley SD, Parkhill J, Harris SR.** 2015. Rapid phylogenetic analysis of large samples of recombinant bacterial whole genome sequences using Gubbins. Nucleic Acids Res **43:**e15.

25. **Marttinen P, Hanage WP, Croucher NJ, Connor TR, Harris SR, Bentley SD, Corander J.** 2012. Detection of recombination events in bacterial genomes from large population samples. Nucleic Acids Res **40:**e6.

26. **Cheng L, Connor TR, Siren J, Aanensen DM, Corander J.** 2013. Hierarchical and spatially explicit clustering of DNA sequences with BAPS software. Mol Biol Evol **30:**1224-1228.

27. **Stamatakis A.** 2014. RAxML version 8: a tool for phylogenetic analysis and post-analysis of large phylogenies. Bioinformatics **30:**1312-1313.

28. **Kloepper TH, Huson DH.** 2008. Drawing explicit phylogenetic networks and their integration into SplitsTree. BMC Evol Biol **8:**22.

29. **Tamura K, Stecher G, Peterson D, Filipski A, Kumar S.** 2013. MEGA6: Molecular Evolutionary Genetics Analysis version 6.0. Mol Biol Evol **30:**2725-2729.

30. **Fouts DE, Brinkac L, Beck E, Inman J, Sutton G.** 2012. PanOCT: automated clustering of orthologs using conserved gene neighborhood for pan-genomic analysis of bacterial strains and closely related species. Nucleic Acids Res **40:**e172.

31. **Trapnell C, Roberts A, Goff L, Pertea G, Kim D, Kelley DR, Pimentel H, Salzberg SL, Rinn JL, Pachter L.** 2012. Differential gene and transcript expression analysis of RNA-seq experiments with TopHat and Cufflinks. Nat Protoc **7:**562-578.

32. **Olsen RJ, Fittipaldi N, Kachroo P, Sanson MA, Long SW, Como-Sabetti KJ, Valson C, Cantu C, Lynfield R, Van Beneden C, Beres SB, Musser JM.** 2014. Clinical laboratory response to a mock outbreak of invasive bacterial infections: a preparedness study. J Clin Microbiol **52:**4210-4216.

33. **Magoc T, Wood D, Salzberg SL.** 2013. EDGE-pro: Estimated Degree of Gene Expression in Prokaryotic Genomes. Evol Bioinform Online **9:**127-136.

34. **Love MI, Huber W, Anders S.** 2014. Moderated estimation of fold change and dispersion for RNA-seq data with DESeq2. Genome Biol **15:**550.

35. **Zhu L, Olsen RJ, Nasser W, de la Riva Morales I, Musser JM.** 2015. Trading Capsule for Increased Cytotoxin Production: Contribution to Virulence of a Newly Emerged Clade of emm89 Streptococcus pyogenes. MBio **6:**e01378-01315.

36. **Olsen RJ, Musser JM.** 2010. Molecular pathogenesis of necrotizing fasciitis. Annu Rev Pathol **5:**1-31.

37. **Olsen RJ, Sitkiewicz I, Ayeras AA, Gonulal VE, Cantu C, Beres SB, Green NM, Lei B, Humbird T, Greaver J, Chang E, Ragasa WP, Montgomery CA, Cartwright J, Jr., McGeer A, Low DE, Whitney AR, Cagle PT, Blasdel TL, DeLeo FR, Musser JM.** 2010. Decreased necrotizing fasciitis capacity caused by a single nucleotide mutation that alters a multiple gene virulence axis. Proc Natl Acad Sci U S A **107:**888-893.
