# Supplementary figures and images for: Transcriptome Remodeling Contributes to Epidemic Disease Caused by the Human Pathogen Streptococcus pyogenes
Source: mBio. 2016 May 31;7(3):e00403-16. doi: 10.1128/mBio.00403-16 (PMC4895104; doi:10.1128/mBio.00403-16)

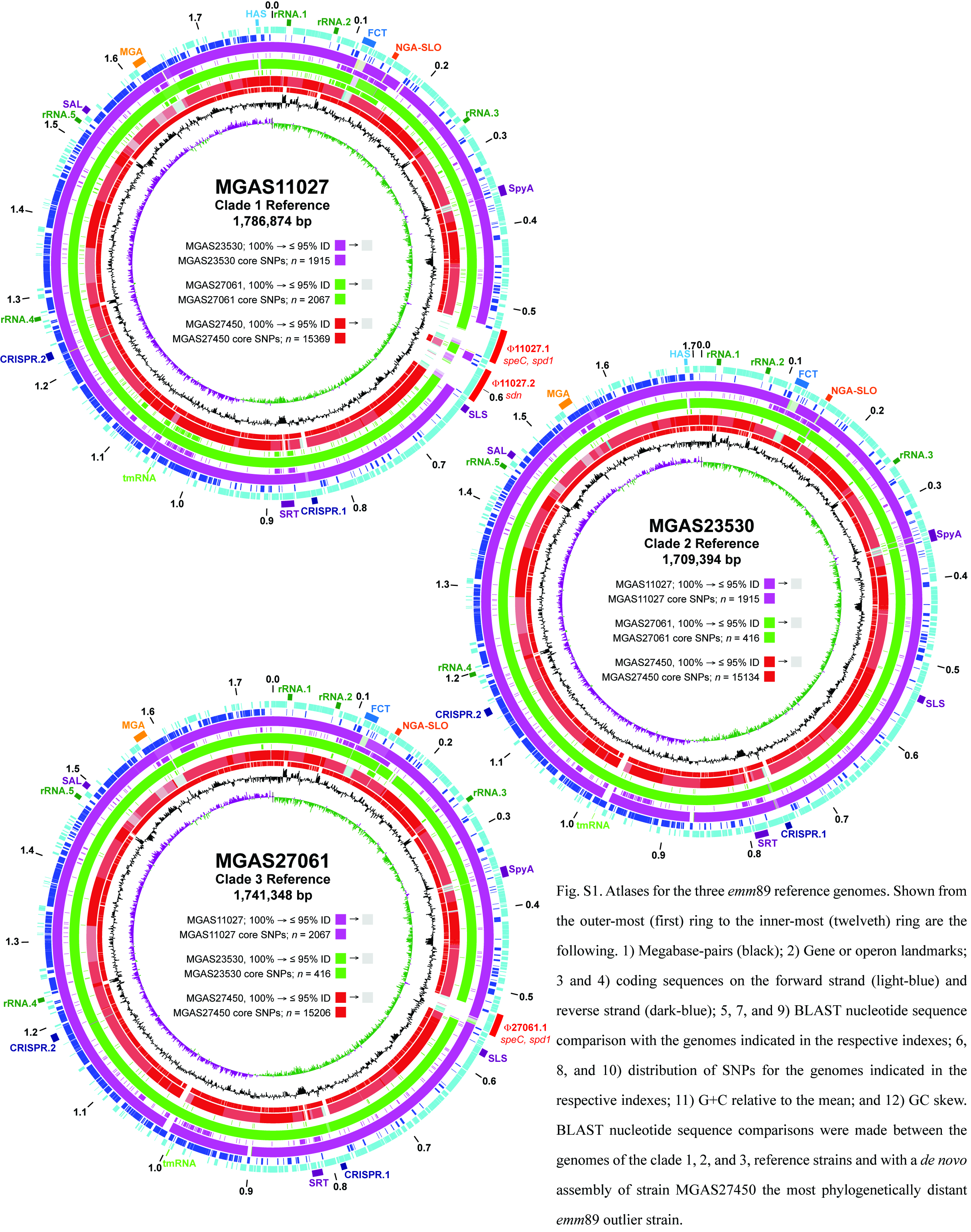

Supplement: Figure S1 — Atlases for the three emm89 reference genomes. Shown from the outermost (1st) ring to the innermost (12th) ring are the following: ring 1, megabase pairs (black); ring 2, gene or operon landmarks; rings 3 and 4, coding sequences on the forward strand (light blue) and reverse strand (dark blue); rings 5, 7, and 9, BLAST nucleotide sequence comparison with the genomes indicated in the respective indexes; rings 6, 8, and 10, distribution of SNPs for the genomes indicated in the respective indexes; ring 11, G+C relative to the mean; and ring 12, GC skew. BLAST nucleotide sequence comparisons were made between the genomes of the clade 1, 2, and 3 reference strains and with a de novo assembly of strain MGAS27450, the most phylogenetically distant emm89 outlier strain. Download [file mbo003162837sf1.tif]
