# Supplementary material for: Transcriptome Remodeling Contributes to Epidemic Disease Caused by the Human Pathogen Streptococcus pyogenes
Source: mBio. 2016 May 31;7(3):e00403-16. doi: 10.1128/mBio.00403-16 (PMC4895104; doi:10.1128/mBio.00403-16)
Supplement: Figure S2 — Genetic relationships among emm89 reference strains, with emm1 reference strain SF370 used as the rooting outgroup. Genetic relationships among the three emm89 reference strains and the seven outlier strains are shown using emm1 reference strain SF370 as an outgroup. Relationships were inferred based on 26,371 core SNPs by neighbor-network decomposition of splits. The sequence of branching of the three numerically dominant emm89 primary clades along the evolutionary path leading to the contemporary epidemic emm89 strains is clade 1 (MGAS11027) followed by clade 2 (MGAS23530) and then epidemic clade 3 (MGAS27061). Download [file mbo003162837sf2.pdf]

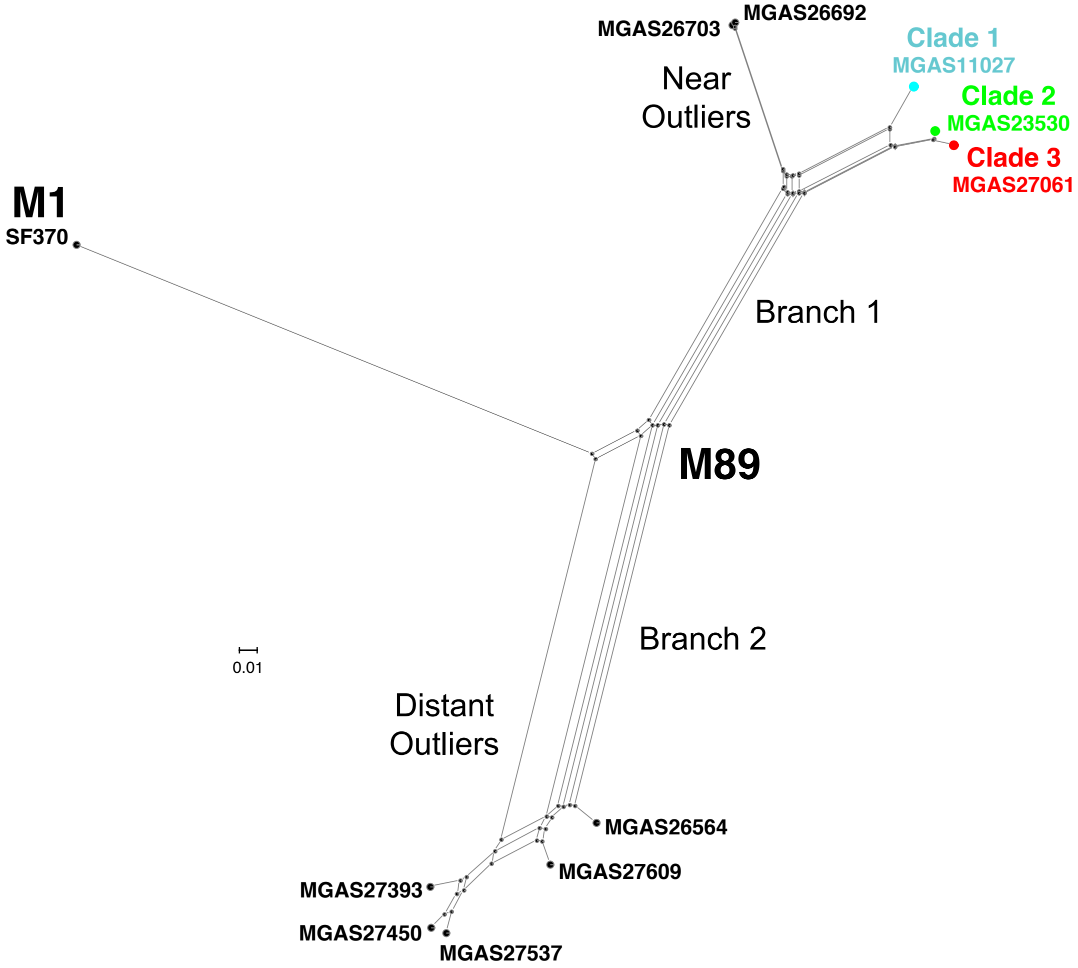

Fig. S2. Genetic relationships among *emm*89 reference strain, with *emm*1 reference strain SF370 used as the rooting outgroup. Shown are genetic relationships among the three *emm*89 reference strains and the seven outlier strains using *emm*1 reference strain SF370 as an outgroup. Relationships were inferred based on 26,371 core SNPs by neighbor-network splits-decomposition. The sequence of branching of the three numerically dominant *emm*89 primary clades along the evolutionary path leading to the contemporary epidemic *emm*89 strains is clade1 (MGAS11027), followed by clade 2 (MGAS23530) and then epidemic clade 3 (MGAS27061).
