# Supplementary material for: Transcriptome Remodeling Contributes to Epidemic Disease Caused by the Human Pathogen Streptococcus pyogenes
Source: mBio. 2016 May 31;7(3):e00403-16. doi: 10.1128/mBio.00403-16 (PMC4895104; doi:10.1128/mBio.00403-16)
Supplement: Figure S3 — Potential horizontal gene transfer (HGT) region donors. The genetic relationships among the three emm89 clade reference strains and 39 strains of 18 other emm-types for which there were complete genome sequences publically available as of 10 July 2015 are shown for each of the predicted recombination blocks (RB) separating the clades. Sequences flanking the predicted recombination blocks in the strain MGAS23530 genome were used to define the corresponding regions in the other strains using BLASTn. The sequences corresponding to the predicted recombination blocks among all 42 strains were aligned using MAFFT, and relationships were inferred by neighbor-network decomposition of splits using SplitsTree. The length of the recombination block and locus tags of the genes involved are listed relative to strain MGAS23530. Of note, seven of the eight recombination blocks separating all 359 clade 1 strains from all 78 clade 2 strains share a more recent common ancestor with emm2 reference strain MGAS10270 than with reference strains of any of the other emm-types used in this comparison. Download [file mbo003162837sf3.pdf]

Fig. S3. Potential HGT region donors

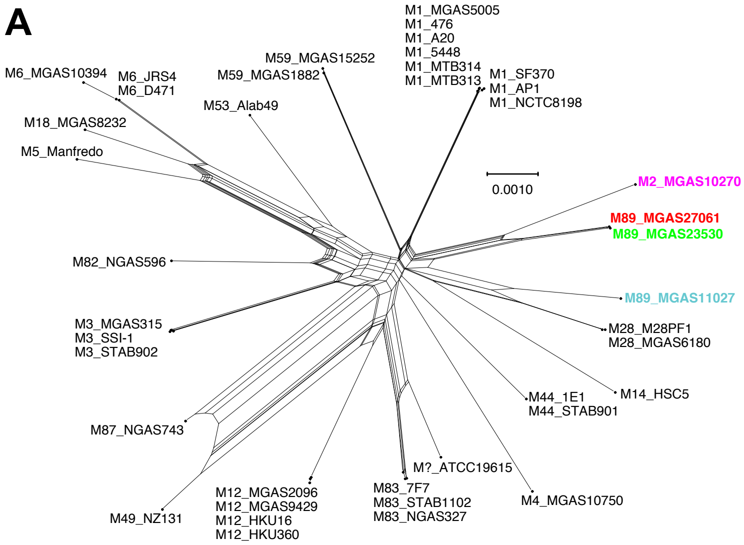

RB1 5'-end C1 to C2, 18618 bp; MGAS23530_0120 - 0142

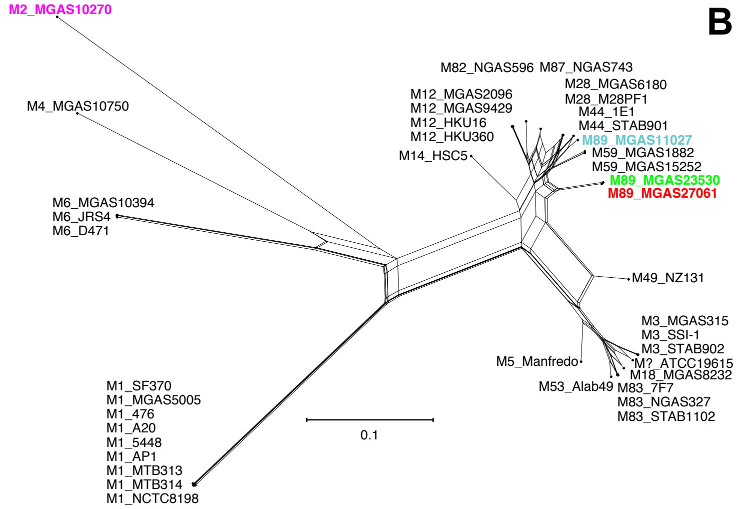

RB1 FCT C1 to C2, 13525 bp; MGAS23530_0143 - 0152

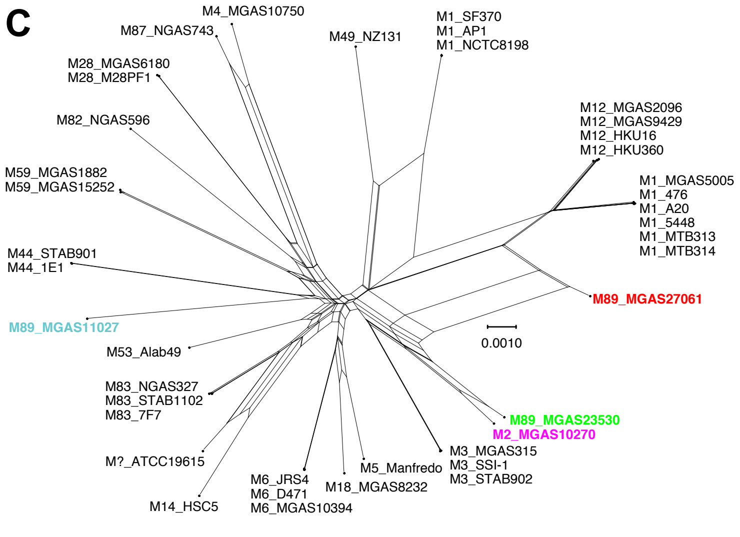

RB1 3'-end C1 to C2, 39631 bp; MGAS23530_0153 - 0191

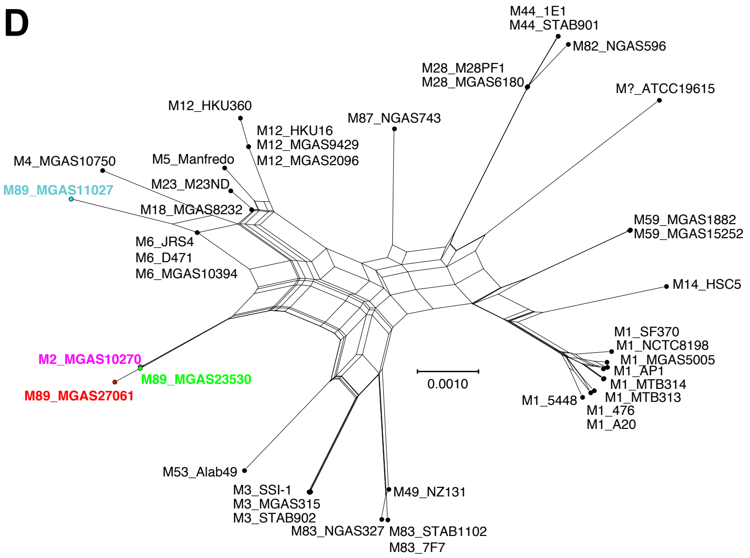

RB2 C1 to C2, 2094 bp; MGAS23530_0315 - 0316

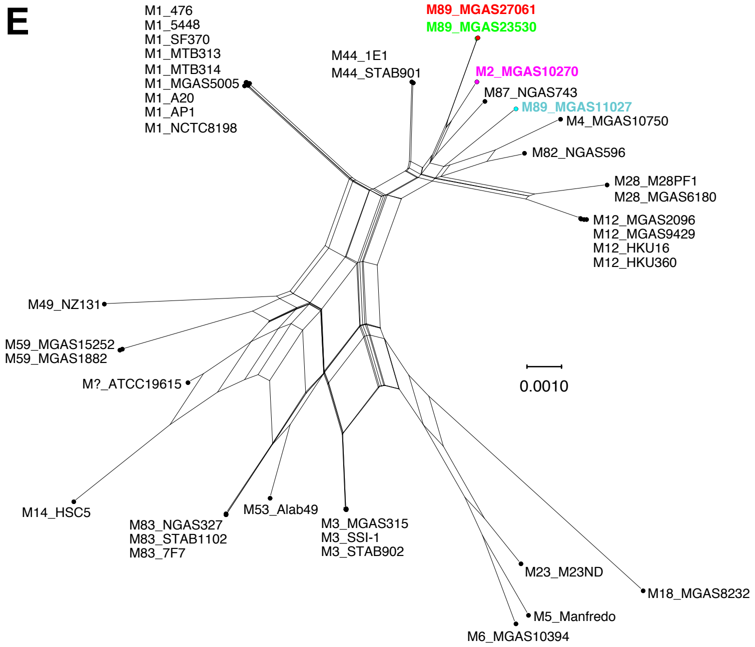

RB3-4 C1 to C2, 27172bp; MGAS23530_0786-0812

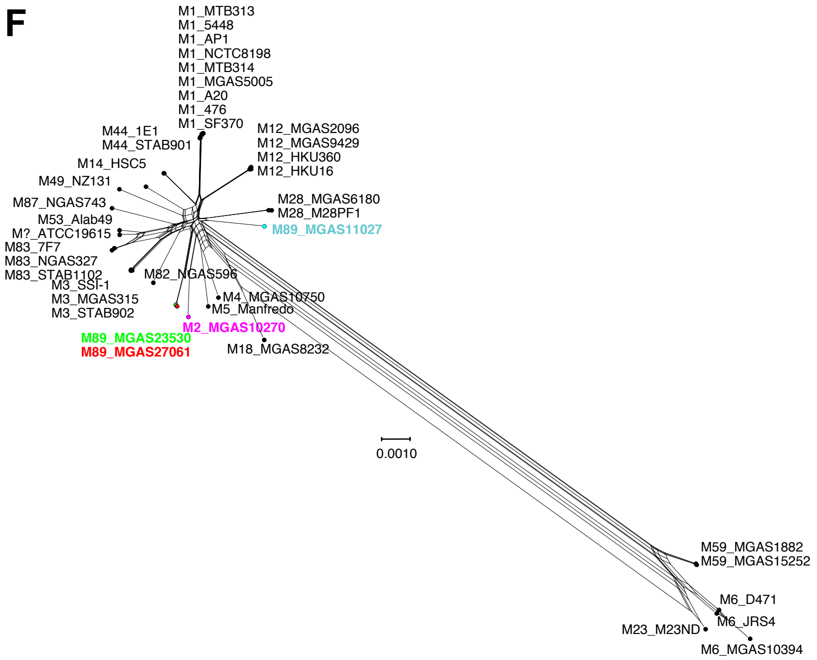

RB5 C1 to C2, 39037bp; MGAS23530_0930-0970

Fig. S3. Potential HGT region donors

G

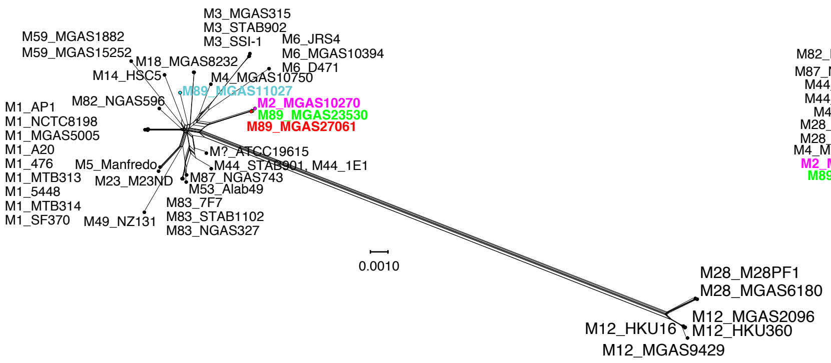

RB6 C1 to C2, 7789 bp; MGAS23530_1027 - 1032

H

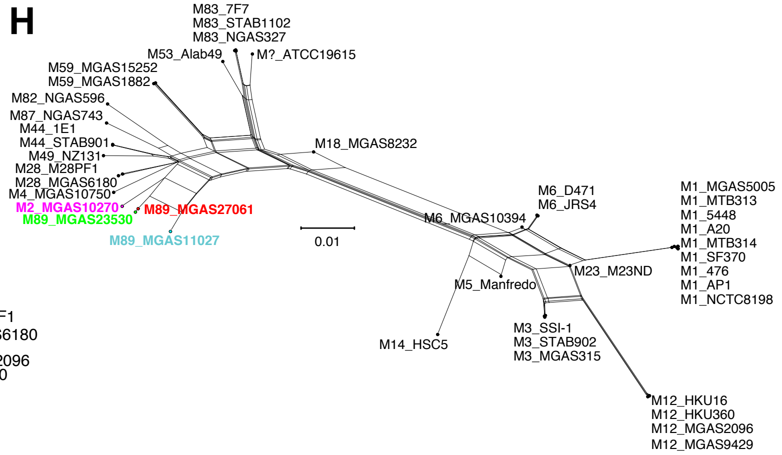

RB7 C1 to C2, 17515 bp; MGAS23530_1532 - 1544

I

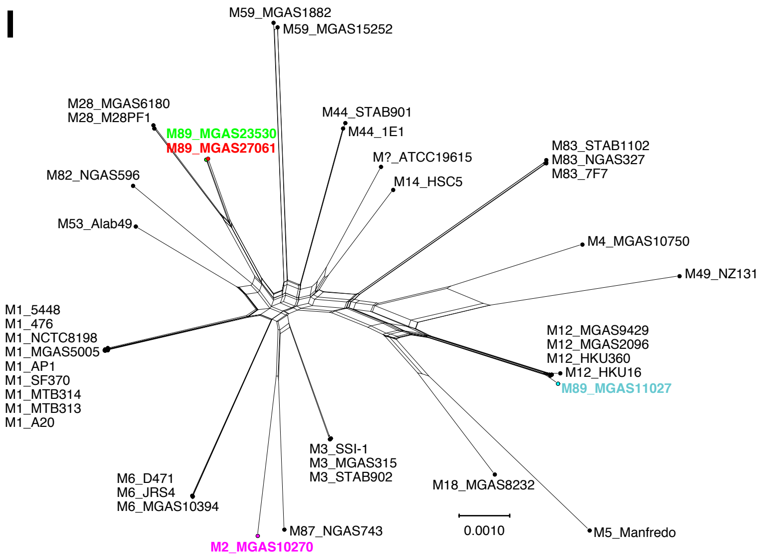

RB8 C1 to C2, 19532 bp; MGAS23530_1561 - 1581

J

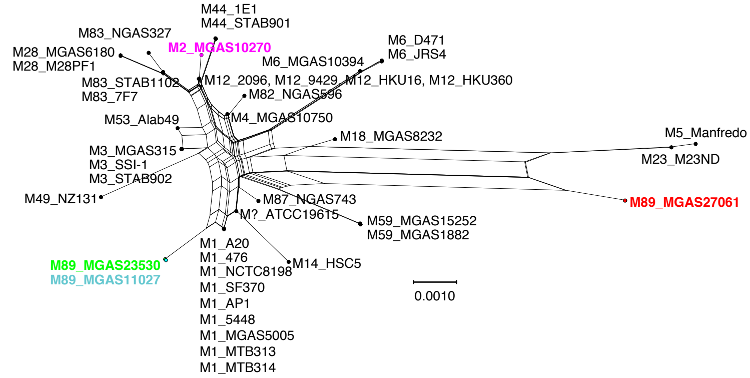

RB9 C2 to C3, 1764 bp; MGAS23530_0118 - 0119

K

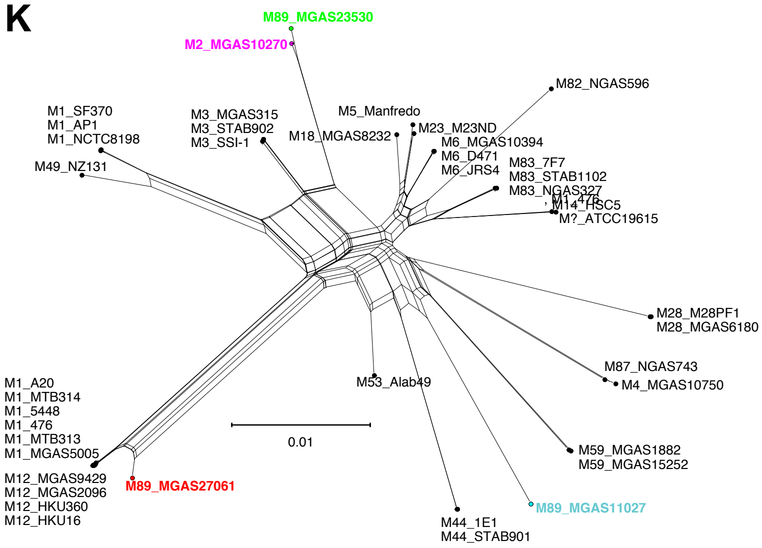

RB10 C2 to C3, 10407 bp; MGAS23530_0173-0183

L

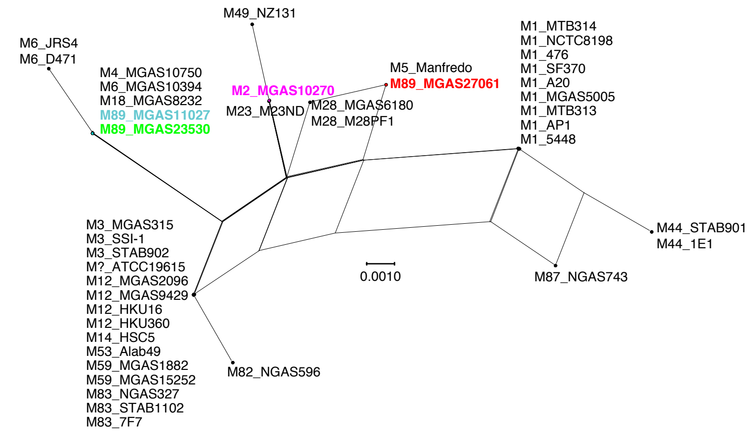

RB11 C2 to C3, 352 bp; MGAS23530_0265

Fig. S3. Potential HGT region donors

M

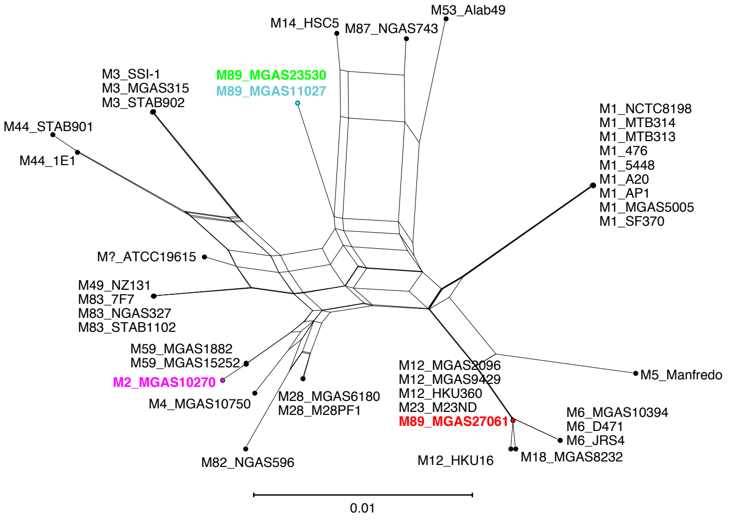

RB12 C2 to C3, 764 bp; MGAS23530_1464

N

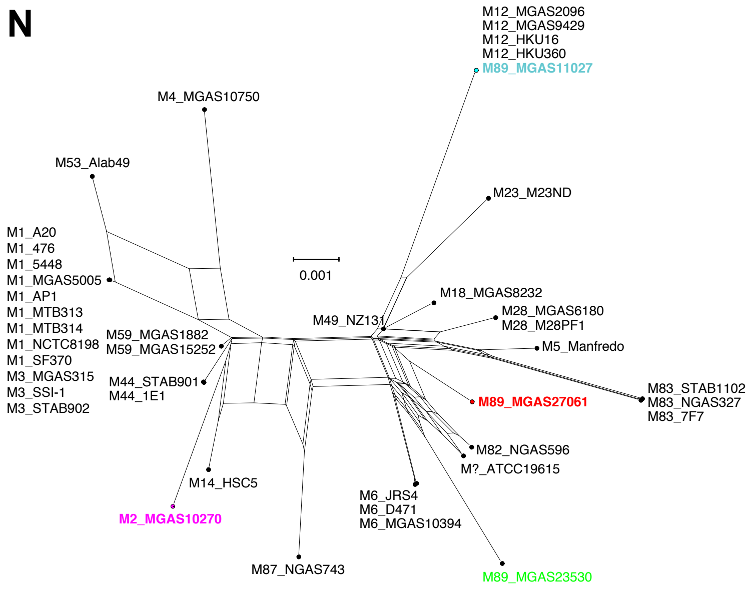

RB13 C2 to C3, 801 bp; MGAS23530_1543 - 1544

O

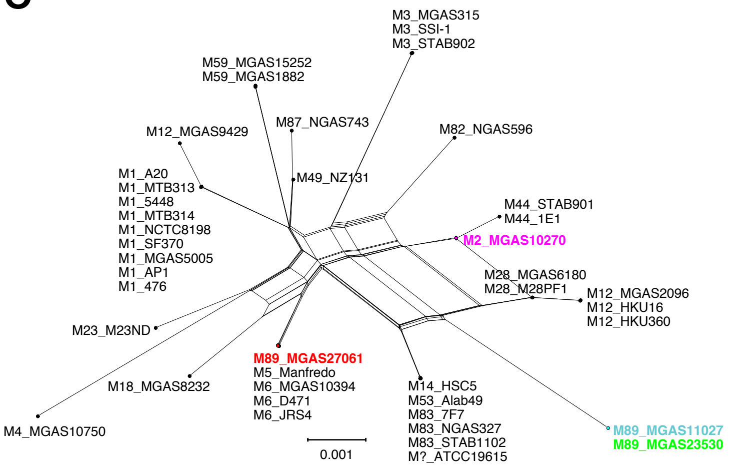

RB14 C2 to C3, 1193 bp; MGAS23530_1670 - 1671

P

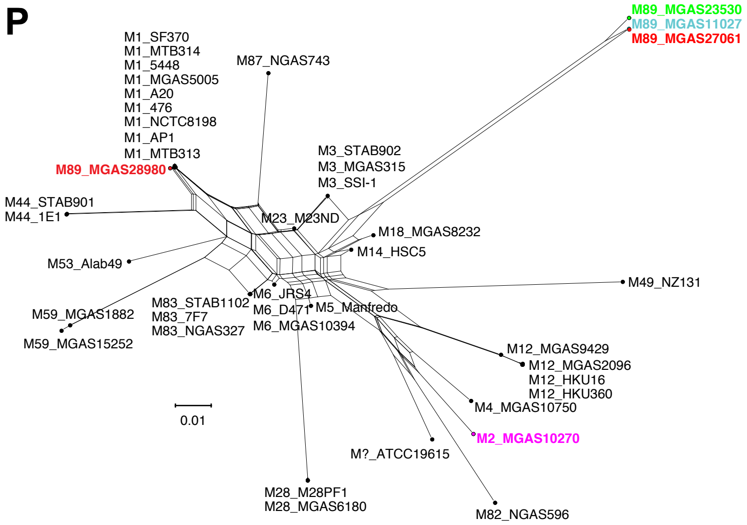

RB15 C3 to SC3D, 17818 bp; MGAS23530_0365 - 0385

Fig. S3. Potential horizontal gene transfer (HGT) region donors. Shown for each of the predicted recombination blocks (RB) separating the clades are the genetic relationships among the three *emm*89 clade reference strains and 39 strains of 18 other *emm* types for which there are complete genome sequences publically available as of July 10, 2015. Sequences flanking the predicted recombination blocks in the strain MGAS23530 genome were used to define the corresponding regions in the other strains using blastn. The sequences corresponding to the predicted recombination blocks among all 42 strains were aligned using MAFFT, and relationships were inferred by neighbor-network split-decomposition using SplitsTree. The length of the recombination block and locus tags of the genes involved are listed relative to strain MGAS23530. Of note, seven of the eight recombination blocks separating all 359 clade 1 strains from all 78 clade 2 strains share a more recent common ancestor with *emm*2 reference strain MGAS10270 than with reference strains of any of the other *emm*-types used in this comparison.
