# Supplementary material for: Transcriptome Remodeling Contributes to Epidemic Disease Caused by the Human Pathogen Streptococcus pyogenes
Source: mBio. 2016 May 31;7(3):e00403-16. doi: 10.1128/mBio.00403-16 (PMC4895104; doi:10.1128/mBio.00403-16)
Supplement: Figure S4 — Genetic relationships among emm89 subclade 3D strains. Genetic relationships among the 33 subclade 3D strains are shown using clade 3 reference strain MGAS27061 as the outgroup. Relationships were inferred based on 157 core SNPs by neighbor-joining using SplitsTree. All subclade 3D strains differ from all progenitor clade 3 strains by an 18 kb region of HGT involving the virulence factors SpyA and SpeJ (Table 1; recombination block 15). To constrain the inference primarily to vertically inherited SNPs, SNPs within putative regions of HGT were identified and filtered out using Gubbins. The 11 strains with the CovR (S130N) substitution branch together, indicating inheritance by descent. Similarly, all but 1 of the 6 strains with the LiaS (K214R) substitution branch together, again indicating inheritance by descent. We attribute the single LiaS (K214R) strain not branching with the others as likely being due to a few scant horizontally acquired polymorphisms that were insufficient to statistically significantly elevate the SNP density and therefore were not detected/excluded by Gubbins. Download [file mbo003162837sf4.pdf]

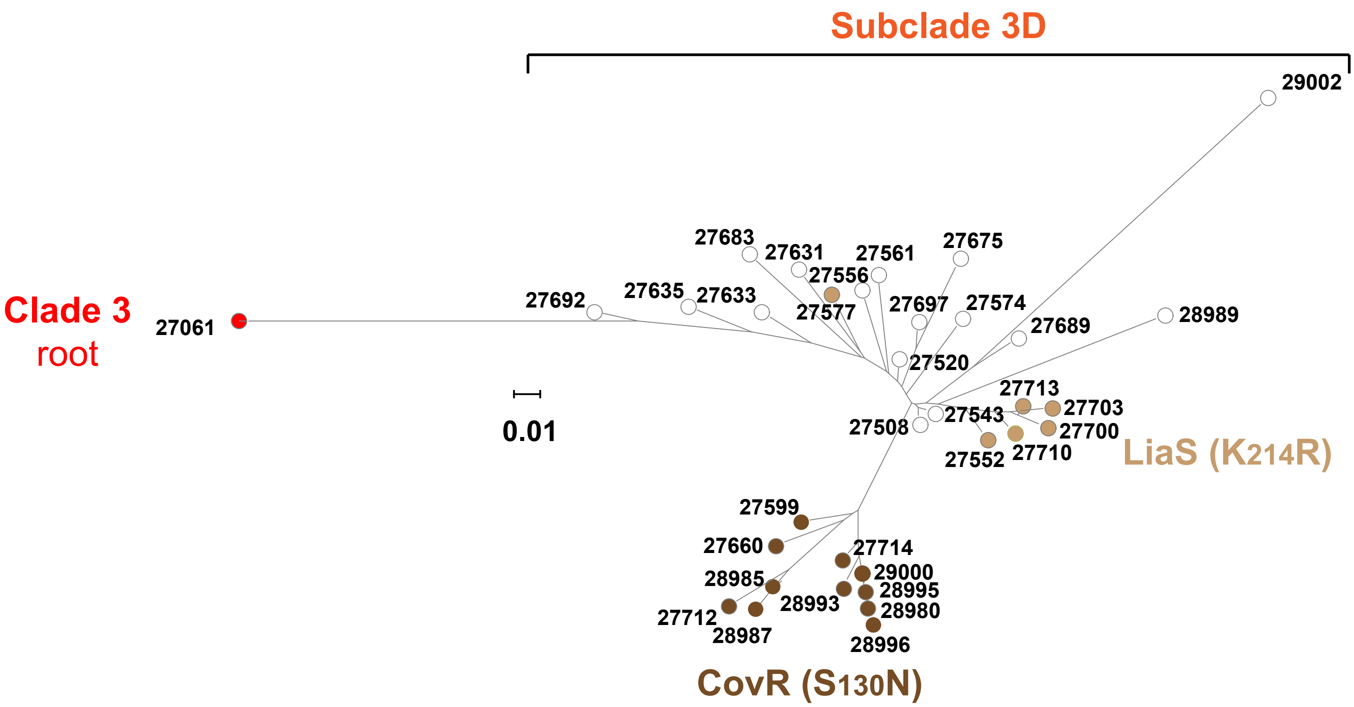

Fig. S4. Genetic relationships among *emm*89 subclade 3D strains. Shown are genetic relationships among the 33 subclade 3D strains using clade 3 reference strain MGAS27061 as an outgroup. Relationships were inferred based on 157 core SNPs by neighbor-joining using SplitsTree. All subclade 3D strains differ from all progenitor clade 3 strains by an 18 kb region of HGT involving the virulence factors SpyA and SpeJ (Table 1, recombination block 15). To constrain the inference primarily to vertically inherited SNPs, SNPs within putative regions of HGT were identified and filtered out using GUBBINs. The 11 strains with the CovR (S130N) substitution branch together indicating inheritance by descent. Similarly all but one of the 6 strains with the LiaS (K214R) susbstitution branch together again indicating inheritance by descent. We attribute the single LiaS (K214R) strain not branching with the others as likely being due to a few scant horizontally acquired polymorphisms that were insufficient to statistically significantly elevate the SNP density and therefore were not detected/excluded by GUBBINs.
