# Supplementary material for: Transcriptome Remodeling Contributes to Epidemic Disease Caused by the Human Pathogen Streptococcus pyogenes
Source: mBio. 2016 May 31;7(3):e00403-16. doi: 10.1128/mBio.00403-16 (PMC4895104; doi:10.1128/mBio.00403-16)
Supplement: Figure S5 — Comparison of phages 11027.1 and 27061.1. Shown above is a percent identity plot, and below is a dot matrix alignment. The phages are similar over the 5′ first ~13 kb sequence, which includes the integrase, replication, and lytic/lysogenic regulatory genes; the phages diverge over most of the central portions encoding head and tail coat proteins and then are similar again over the 3′ last ~3 kb sequence, which encodes the secreted virulence factors streptococcal pyrogenic exotoxin C (SpeC) superantigen and the streptococcal phage DNase 1 (Spd1). The divergence in sequence between phages 11027.1 and 27061.1 means that 27061.1 did not evolve from 11027.1 through a simple single deletion event. Despite being integrated at the same genomic locus and encoding the same virulence factors, they are distinct mosaic phages. Download [file mbo003162837sf5.pdf]

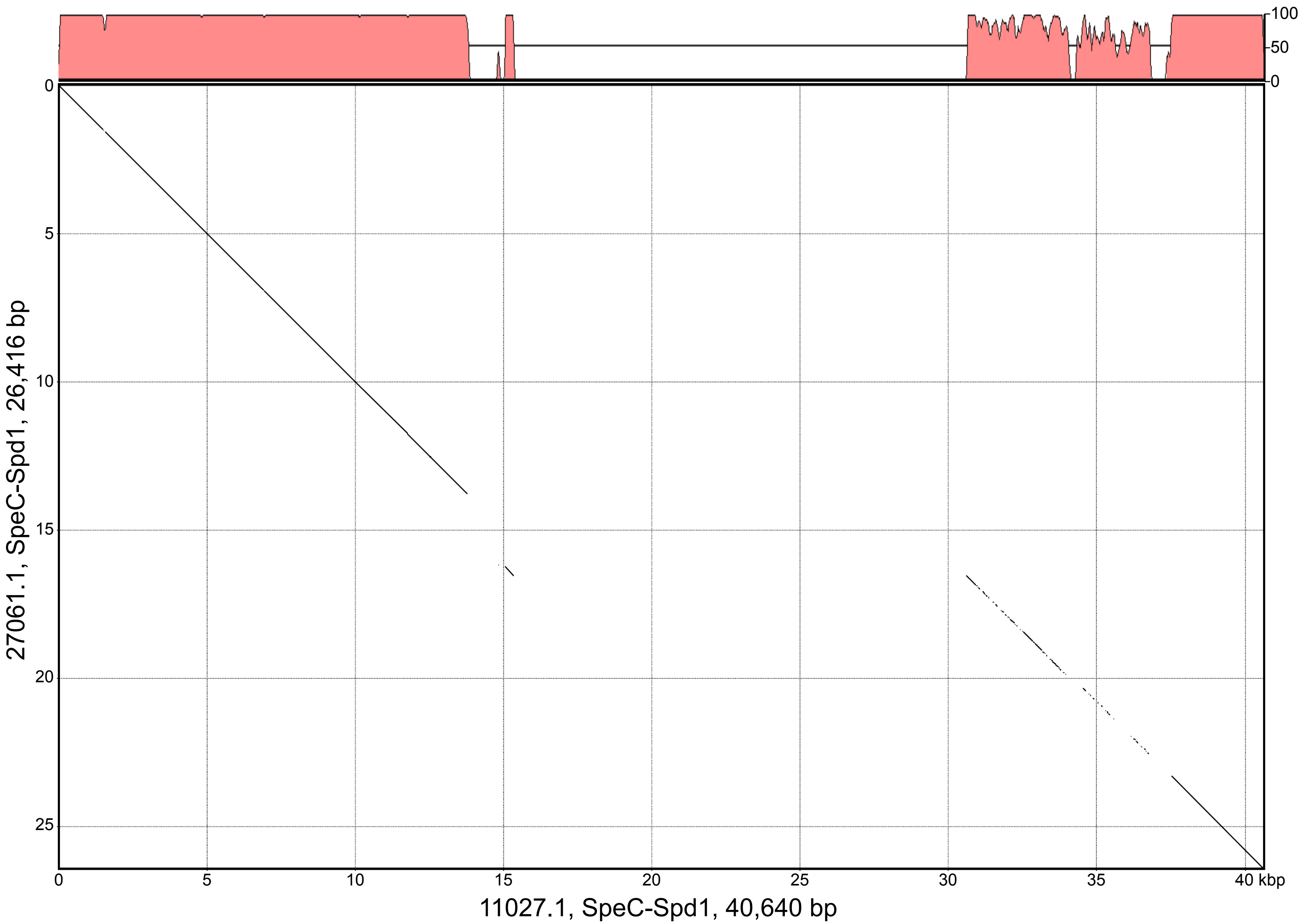

Fig. S5. Comparison of phages 11027.1 and 27061.1. Shown above is a percent identity plot and below is a dot matrix alignment. The phages are similar over the 5' first ~13 kb encoding the integrase, replication and lytic/lysogenic regulatory genes, diverge over most of the central portions encoding head and tail coat proteins, and then are similar again over the 3' last ~3 kb encoding the secreted virulence factors streptococcal pyrogenic exotoxin C (SpeC) superantigen and the streptococcal phage DNase 1 (Spd1). The divergence in sequence between 11027.1 and 27061.1 means that 27061.1 did not evolve from 11027.1 through a simple single deletion event. Despite being integrated at the same genomic locus and encoding the same virulence factors, they are distinct mosaic phages.
