# Supplementary material for: Transcriptome Remodeling Contributes to Epidemic Disease Caused by the Human Pathogen Streptococcus pyogenes
Source: mBio. 2016 May 31;7(3):e00403-16. doi: 10.1128/mBio.00403-16 (PMC4895104; doi:10.1128/mBio.00403-16)
Supplement: Figure S6 — hasABC promoter variants. (A) hasABC promoter pattern variants identified among the emm89 clade 1 and clade 2 strains are illustrated. Patterns A and B account for 99% of the strains. Pattern B has a 38-bp deletion relative to pattern A which eliminates a putative Rho-independent terminator. In M1 strain MGAS2221, deletion of this terminator results in release of hasABC from CovR repression, resulting in enhanced capsule production. (B) Distribution of hasABC promoter variants among the clade 1 and 2 strains. (C) Distribution of hasABC promoter variants among the clade 1 and clade 2 strains. Genetic relationships among the emm89 clade 1 and clade 2 strains inferred by neighbor-joining based on 5,663 core SNPs filtered using Gubbins to exclude regions of horizontal gene transfer are illustrated. Strains are colored by promoter variant as indicated in the index. Clade 1 strains are a mix of nearly equal amounts of pattern A (weak/repressed) and pattern B (strong/derepressed) promoter variants, whereas the vast majority of clade 2 strains are of pattern A. These findings are consistent with the level of hasABC transcripts for clade 1 strain MGAS11027 being significantly greater than that for clade 2 strain MGAS23530 as determined by RNAseq. Download [file mbo003162837sf6.pdf]

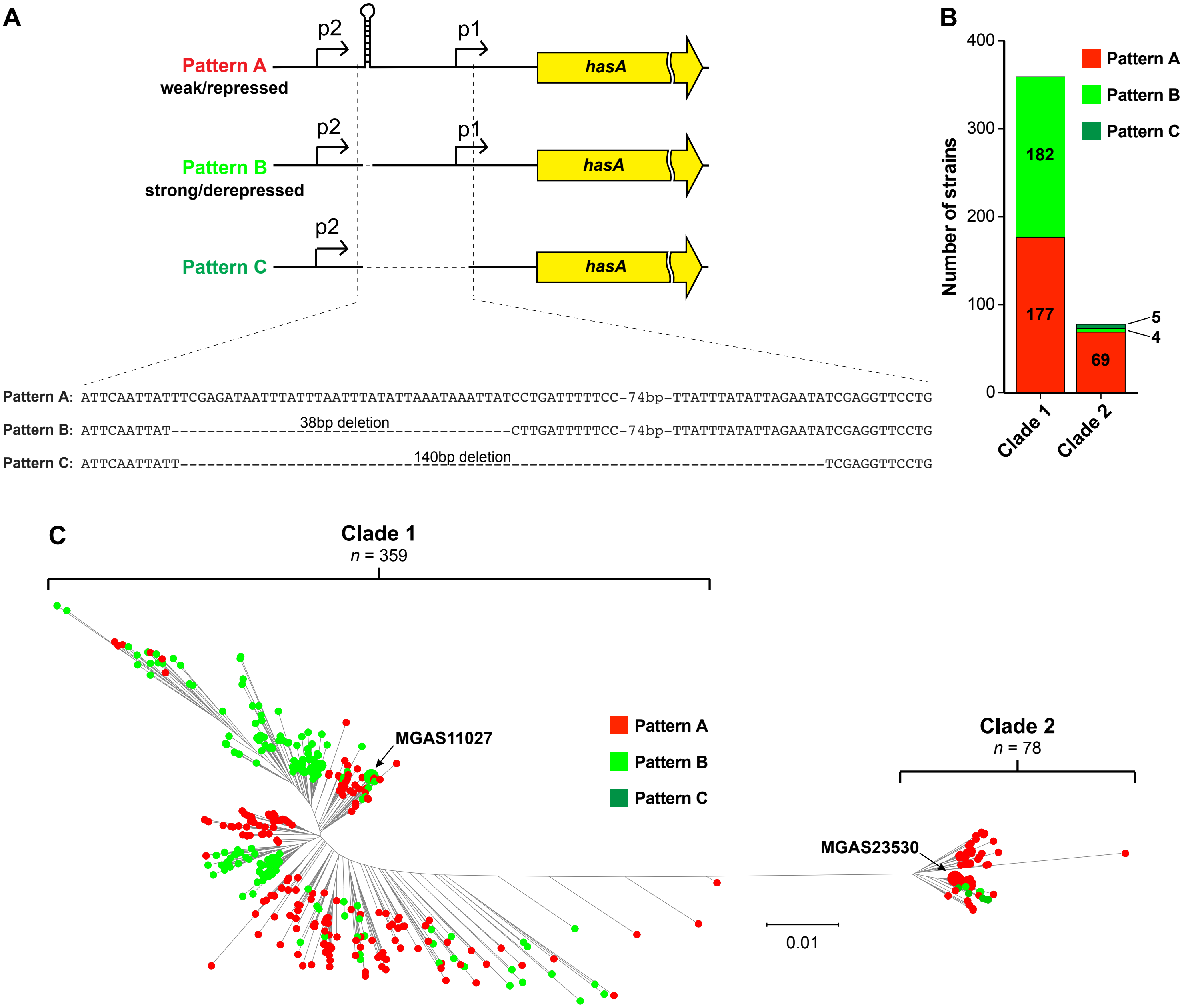

Fig. S6. *hasABC* promoter variants. (*A*) Illustrated are *hasABC* promoter pattern variants identified among the *emm*89 clade 1 and and 2 strains. Patterns A and B account for 99% of the strains. Pattern B has a 38 bp deletion relative to pattern A, which eliminates a putative Rho independent terminator. In M1 strain MGAS2221, deletion of this terminator results in release of *hasABC* from CovR repression resulting in enhanced capsule production. (*B*) Distribution of *hasABC* promoter variants among the clade 1 and 2 strains. (*C*) Distribution of *hasABC* promoter variants among the clade 1 and 2 strains. Illustrated is genetic relationships among the *emm*89 clade 1 and 2 strains inferred by neighbor-joining based on 5,663 core SNPs filtered using GUBBINs to exclude regions of horizontal gene transfer. Strains are colored by promoter variant as indicated in the index. Clade 1 strains are a nearly equal mix of pattern A (weak/repressed) and pattern B (strong/derepressed) promoter variants, whereas the vast majority of clade 2 strains are pattern A. These findings are consistent with the significantly greater level of *hasABC* transcripts for clade 1 strain MGAS11027 relative to clade 2 strain MGAS23530 determined by RNAseq.
