# Supplementary material for: Transcriptome Remodeling Contributes to Epidemic Disease Caused by the Human Pathogen Streptococcus pyogenes
Source: mBio. 2016 May 31;7(3):e00403-16. doi: 10.1128/mBio.00403-16 (PMC4895104; doi:10.1128/mBio.00403-16)
Supplement: Table S1 — Strains and characteristics. [file mbo003162837st1.docx]

**Supplemental Table S1. Strains and Characteristics**

| **No.** | **Strain** | **Country** | **Region** | **Year** | **Infection** | **Clade** | ***nga-ifs-slo***  **promoter**  **variant^a^** | ***hasABC***  **promoter**  **variant** | **MLST^b^** | **Phage-Type** |
| --- | --- | --- | --- | --- | --- | --- | --- | --- | --- | --- |
| 1 | MGAS11027 | United States | TX | 2002 | Pharyngitis | 1 | 1 | B | 407 | 3 |
| 2 | MGAS26563 | United States | GA | 1995 | Invasive | 1 | 1 | A | NF | 46 |
| 3 | MGAS26565 | United States | CT | 1995 | Invasive | 1 | 1 | A | 407 | 64 |
| 4 | MGAS26566 | United States | CT | 1995 | Invasive | 1 | 1 | A | 407 | 3 |
| 5 | MGAS26567 | United States | CT | 1995 | Invasive | 1 | 1 | A | 407 | 5 |
| 6 | MGAS26568 | United States | GA | 1996 | Invasive | 1 | 1 | A | 407 | 3 |
| 7 | MGAS26569 | United States | MN | 1995 | Invasive | 1 | 1 | A | 407 | 3 |
| 8 | MGAS26570 | United States | MN | 1995 | Invasive | 1 | 1 | A | 407 | 10 |
| 9 | MGAS26573 | United States | MD | 2009 | Invasive | 1 | 1 | B | 407 | 12 |
| 10 | MGAS26574 | United States | MD | 2009 | Invasive | 1 | 1 | B | 407 | 12 |
| 11 | MGAS26576 | United States | NY | 2010 | Invasive | 1 | 1 | B | 407 | 5 |
| 12 | MGAS26578 | United States | CA | 2010 | Invasive | 1 | 1 | A | 407 | 3 |
| 13 | MGAS26579 | United States | TN | 2004 | Invasive | 1 | 1 | A | 407 | 9 |
| 14 | MGAS26580 | United States | OR | 2005 | Invasive | 1 | 1 | B | 407 | 6 |
| 15 | MGAS26581 | United States | OR | 2005 | Invasive | 1 | 1 | B | 407 | 6 |
| 16 | MGAS26582 | United States | MN | 2005 | Invasive | 1 | 1 | B | 407 | 3 |
| 17 | MGAS26584 | United States | GA | 2005 | Invasive | 1 | 1 | A | 407 | 3 |
| 18 | MGAS26585 | United States | CO | 2005 | Invasive | 1 | 1 | B | 407 | 3 |
| 19 | MGAS26586 | United States | MN | 2005 | Invasive | 1 | 1 | B | 407 | 3 |
| 20 | MGAS26587 | United States | MN | 2005 | Invasive | 1 | 1 | A | 407 | 10 |
| 21 | MGAS26588 | United States | MN | 2005 | Invasive | 1 | 1 | B | 407 | 3 |
| 22 | MGAS26589 | United States | MN | 2005 | Invasive | 1 | 1 | B | 407 | 3 |
| 23 | MGAS26590 | United States | OR | 2005 | Invasive | 1 | 1 | B | 407 | 6 |
| 24 | MGAS26591 | United States | OR | 2005 | Invasive | 1 | 1 | B | 407 | 6 |
| 25 | MGAS26592 | United States | GA | 2005 | Invasive | 1 | 1 | B | 407 | 3 |
| 26 | MGAS26593 | United States | CO | 2005 | Invasive | 1 | 1 | B | 407 | 3 |
| 27 | MGAS26595 | United States | OR | 2005 | Invasive | 1 | 1 | B | 407 | 6 |
| 28 | MGAS26596 | United States | MN | 2005 | Invasive | 1 | 1 | B | 407 | 3 |
| 29 | MGAS26598 | United States | TN | 2005 | Invasive | 1 | 1 | A | 407 | 3 |
| 30 | MGAS26599 | United States | CO | 2005 | Invasive | 1 | 1 | B | 407 | 3 |
| 31 | MGAS26600 | United States | GA | 2005 | Invasive | 1 | 1 | B | 407 | 21 |
| 32 | MGAS26601 | United States | MD | 2005 | Invasive | 1 | 1 | A | 407 | 2 |
| 33 | MGAS26602 | United States | NY | 2006 | Invasive | 1 | 1 | A | 407 | 3 |
| 34 | MGAS26604 | United States | OR | 2006 | Invasive | 1 | 1 | B | 407 | 10 |
| 35 | MGAS26605 | United States | GA | 2006 | Invasive | 1 | 1 | A | 407 | 54 |
| 36 | MGAS26606 | United States | MN | 2005 | Invasive | 1 | 1 | B | 407 | 3 |
| 37 | MGAS26607 | United States | MN | 2005 | Invasive | 1 | 1 | A | 407 | 6 |
| 38 | MGAS26608 | United States | CO | 2005 | Invasive | 1 | 1 | B | 407 | 12 |
| 39 | MGAS26610 | United States | CO | 2005 | Invasive | 1 | 1 | B | 407 | 3 |
| 40 | MGAS26612 | United States | MD | 2005 | Invasive | 1 | 1 | A | 407 | 5 |
| 41 | MGAS26613 | United States | MD | 2005 | Invasive | 1 | 1 | B | 407 | 10 |
| 42 | MGAS26615 | United States | NY | 2006 | Invasive | 1 | 1 | A | 407 | 11 |
| 43 | MGAS26618 | United States | TN | 2008 | Invasive | 1 | 1 | A | 407 | 6 |
| 44 | MGAS26620 | United States | TN | 2008 | Invasive | 1 | 1 | B | 407 | 3 |
| 45 | MGAS26635 | United States | GA | 2009 | Invasive | 1 | 1 | B | 407 | 3 |
| 46 | MGAS26637 | United States | NY | 2009 | Invasive | 1 | 1 | B | 407 | 5 |
| 47 | MGAS26639 | United States | NY | 2009 | Invasive | 1 | 1 | B | 407 | 5 |
| 48 | MGAS26642 | United States | TN | 2009 | Invasive | 1 | 1 | B | 407 | 3 |
| 49 | MGAS26645 | United States | MD | 2009 | Invasive | 1 | 1 | B | 407 | 3 |
| 50 | MGAS26653 | United States | CA | 2009 | Invasive | 1 | 1 | A | 407 | 5 |
| 51 | MGAS26656 | United States | CO | 2009 | Invasive | 1 | 1 | A | 407 | 13 |
| 52 | MGAS26658 | United States | OR | 2010 | Invasive | 1 | 1 | B | 407 | 3 |
| 53 | MGAS26662 | United States | NM | 2009 | Invasive | 1 | 1 | A | 407 | 3 |
| 54 | MGAS26666 | United States | GA | 2009 | Invasive | 1 | 1 | A | 407 | 5 |
| 55 | MGAS26667 | United States | NY | 2009 | Invasive | 1 | 1 | B | 407 | 5 |
| 56 | MGAS26668 | United States | CO | 2009 | Invasive | 1 | 1 | B | 407 | 3 |
| 57 | MGAS26669 | United States | CA | 2009 | Invasive | 1 | 1 | A | 407 | 5 |
| 58 | MGAS26685 | United States | GA | 2009 | Invasive | 1 | 1 | A | 407 | 3 |
| 59 | MGAS26693 | United States | CO | 2001 | Invasive | 1 | 1 | B | 407 | 3 |
| 60 | MGAS26694 | United States | CO | 2001 | Invasive | 1 | 1 | B | 407 | 12 |
| 61 | MGAS26695 | United States | CO | 2001 | Invasive | 1 | 1 | B | 407 | 3 |
| 62 | MGAS26696 | United States | CT | 2001 | Invasive | 1 | 1 | B | 407 | 5 |
| 63 | MGAS26697 | United States | CT | 2001 | Invasive | 1 | 1 | A | 407 | 6 |
| 64 | MGAS26700 | United States | CT | 2001 | Invasive | 1 | 1 | B | 407 | 5 |
| 65 | MGAS26701 | United States | MD | 2002 | Invasive | 1 | 1 | A | 407 | 44 |
| 66 | MGAS26702 | United States | MD | 2002 | Invasive | 1 | 1 | A | 407 | 5 |
| 67 | MGAS26706 | United States | MD | 2002 | Invasive | 1 | 1 | A | 407 | 5 |
| 68 | MGAS26707 | United States | CT | 2001 | Invasive | 1 | 1 | A | 407 | 26 |
| 69 | MGAS26708 | United States | CT | 2001 | Invasive | 1 | 1 | B | 407 | 3 |
| 70 | MGAS26709 | United States | CT | 2001 | Invasive | 1 | 1 | B | 407 | 3 |
| 71 | MGAS26710 | United States | CT | 2001 | Invasive | 1 | 1 | A | 407 | 49 |
| 72 | MGAS26717 | United States | OR | 1998 | Invasive | 1 | 1 | A | 407 | 20 |
| 73 | MGAS26718 | United States | CT | 1998 | Invasive | 1 | 1 | A | 407 | 14 |
| 74 | MGAS26719 | United States | CT | 1998 | Invasive | 1 | 1 | A | 407 | 11 |
| 75 | MGAS26720 | United States | CT | 1998 | Invasive | 1 | 1 | A | 407 | 11 |
| 76 | MGAS26721 | United States | CT | 2002 | Invasive | 1 | 1 | A | 407 | 13 |
| 77 | MGAS26722 | United States | CT | 2002 | Invasive | 1 | 1 | B | 407 | 3 |
| 78 | MGAS26725 | United States | MN | 2002 | Invasive | 1 | 1 | A | 407 | 3 |
| 79 | MGAS26726 | United States | NM | 2010 | Invasive | 1 | 1 | B | 407 | 3 |
| 80 | MGAS26732 | United States | MD | 2010 | Invasive | 1 | 1 | B | 407 | 3 |
| 81 | MGAS26735 | United States | NY | 2002 | Invasive | 1 | 1 | B | 407 | 9 |
| 82 | MGAS26736 | United States | CT | 1998 | Invasive | 1 | 1 | A | 407 | 6 |
| 83 | MGAS26737 | United States | MD | 1998 | Invasive | 1 | 1 | A | 407 | 8 |
| 84 | MGAS26738 | United States | GA | 1999 | Invasive | 1 | 1 | A | 407 | 11 |
| 85 | MGAS26739 | United States | CA | 1999 | Invasive | 1 | 1 | A | 407 | 20 |
| 86 | MGAS26740 | United States | CT | 1999 | Invasive | 1 | 1 | B | 407 | 3 |
| 87 | MGAS26741 | United States | MN | 1999 | Invasive | 1 | 1 | A | 407 | 15 |
| 88 | MGAS26742 | United States | MN | 1999 | Invasive | 1 | 1 | B | 407 | 3 |
| 89 | MGAS26743 | United States | GA | 1999 | Invasive | 1 | 1 | A | 407 | 3 |
| 90 | MGAS26747 | United States | MD | 2002 | Invasive | 1 | 1 | A | 407 | 21 |
| 91 | MGAS26748 | United States | MD | 2002 | Invasive | 1 | 1 | A | 407 | 6 |
| 92 | MGAS26749 | United States | MD | 2002 | Invasive | 1 | 1 | A | 407 | 3 |
| 93 | MGAS26750 | United States | NY | 1999 | Invasive | 1 | 1 | B | 407 | 3 |
| 94 | MGAS26751 | United States | CT | 1999 | Invasive | 1 | 1 | A | 407 | 5 |
| 95 | MGAS26752 | United States | CT | 1999 | Invasive | 1 | 1 | A | 407 | 5 |
| 96 | MGAS26753 | United States | CT | 1999 | Invasive | 1 | 1 | A | 407 | 10 |
| 97 | MGAS26754 | United States | CT | 1999 | Invasive | 1 | 1 | A | 407 | 51 |
| 98 | MGAS26755 | United States | CT | 1999 | Invasive | 1 | 1 | A | 407 | 5 |
| 99 | MGAS26756 | United States | MN | 1998 | Invasive | 1 | 1 | A | 407 | 15 |
| 100 | MGAS26757 | United States | MN | 2002 | Invasive | 1 | 1 | B | 407 | 3 |
| 101 | MGAS26758 | United States | MN | 2002 | Invasive | 1 | 1 | B | 407 | 3 |
| 102 | MGAS26759 | United States | MN | 2002 | Invasive | 1 | 1 | A | 407 | 13 |
| 103 | MGAS26760 | United States | GA | 2002 | Invasive | 1 | 1 | A | 407 | 3 |
| 104 | MGAS26762 | United States | CT | 2002 | Invasive | 1 | 1 | A | 407 | 3 |
| 105 | MGAS26766 | United States | CO | 2001 | Invasive | 1 | 1 | A | 407 | 28 |
| 106 | MGAS26767 | United States | CA | 2002 | Invasive | 1 | 1 | A | 407 | 3 |
| 107 | MGAS26770 | United States | MD | 2002 | Invasive | 1 | 1 | A | 407 | 5 |
| 108 | MGAS26771 | United States | CO | 2002 | Invasive | 1 | 1 | B | NF | 3 |
| 109 | MGAS26772 | United States | CO | 2002 | Invasive | 1 | 1 | B | 407 | 3 |
| 110 | MGAS26774 | United States | MD | 1998 | Invasive | 1 | 1 | A | 407 | 9 |
| 111 | MGAS26776 | United States | GA | 2010 | Invasive | 1 | 1 | B | 407 | 3 |
| 112 | MGAS26780 | United States | GA | 2010 | Invasive | 1 | 1 | A | 407 | 5 |
| 113 | MGAS26785 | United States | MD | 2010 | Invasive | 1 | 1 | B | 407 | 3 |
| 114 | MGAS26795 | United States | NY | 2011 | Invasive | 1 | 1 | B | 407 | 21 |
| 115 | MGAS26798 | United States | MN | 2011 | Invasive | 1 | 1 | A | 407 | 3 |
| 116 | MGAS26807 | United States | CO | 2011 | Invasive | 1 | 1 | B | 407 | 3 |
| 117 | MGAS26814 | United States | GA | 2001 | Invasive | 1 | 1 | A | 407 | 11 |
| 118 | MGAS26821 | United States | MD | 2002 | Invasive | 1 | 1 | A | 407 | 5 |
| 119 | MGAS26822 | United States | MD | 2002 | Invasive | 1 | 1 | A | 407 | 5 |
| 120 | MGAS26823 | United States | CO | 2002 | Invasive | 1 | 1 | B | 407 | 3 |
| 121 | MGAS26824 | United States | CO | 2002 | Invasive | 1 | 1 | B | 407 | 3 |
| 122 | MGAS26827 | United States | MN | 2011 | Invasive | 1 | 1 | B | 407 | 12 |
| 123 | MGAS26829 | United States | CO | 2002 | Invasive | 1 | 1 | B | 407 | 3 |
| 124 | MGAS26830 | United States | NY | 1998 | Invasive | 1 | 1 | B | 407 | 3 |
| 125 | MGAS26832 | United States | CO | 2011 | Invasive | 1 | 1 | B | 407 | 3 |
| 126 | MGAS26833 | United States | GA | 1998 | Invasive | 1 | 1 | A | 407 | 3 |
| 127 | MGAS26834 | United States | MD | 1998 | Invasive | 1 | 1 | A | 407 | 6 |
| 128 | MGAS26835 | United States | MD | 2002 | Invasive | 1 | 1 | B | 407 | 5 |
| 129 | MGAS26838 | United States | TN | 2011 | Invasive | 1 | 1 | A | 407 | 2 |
| 130 | MGAS26839 | United States | MN | 2001 | Invasive | 1 | 1 | A | 407 | 5 |
| 131 | MGAS26840 | United States | MD | 2000 | Invasive | 1 | 1 | B | 407 | 3 |
| 132 | MGAS26841 | United States | MD | 2000 | Invasive | 1 | 1 | A | 407 | 6 |
| 133 | MGAS26843 | United States | CO | 2003 | Invasive | 1 | 1 | B | 407 | 3 |
| 134 | MGAS26847 | United States | TN | 2001 | Invasive | 1 | 1 | A | 407 | 3 |
| 135 | MGAS26848 | United States | CA | 2003 | Invasive | 1 | 1 | A | NF | 5 |
| 136 | MGAS26849 | United States | NY | 1999 | Invasive | 1 | 1 | A | 407 | 5 |
| 137 | MGAS26850 | United States | CA | 1999 | Invasive | 1 | 1 | B | 407 | 3 |
| 138 | MGAS26851 | United States | CT | 2003 | Invasive | 1 | 1 | A | 407 | 3 |
| 139 | MGAS26852 | United States | CT | 2003 | Invasive | 1 | 1 | B | 407 | 3 |
| 140 | MGAS26853 | United States | CT | 2003 | Invasive | 1 | 1 | B | 407 | 3 |
| 141 | MGAS26855 | United States | NY | 2000 | Invasive | 1 | 1 | B | 407 | 3 |
| 142 | MGAS26856 | United States | MN | 2000 | Invasive | 1 | 1 | A | 407 | 6 |
| 143 | MGAS26857 | United States | MN | 2000 | Invasive | 1 | 1 | A | 407 | 5 |
| 144 | MGAS26858 | United States | GA | 2000 | Invasive | 1 | 1 | A | 407 | 3 |
| 145 | MGAS26860 | United States | MD | 2003 | Invasive | 1 | 1 | B | 407 | 3 |
| 146 | MGAS26861 | United States | NY | 2003 | Invasive | 1 | 1 | B | 407 | 5 |
| 147 | MGAS26862 | United States | CT | 2003 | Invasive | 1 | 1 | A | 407 | 5 |
| 148 | MGAS26863 | United States | CT | 2003 | Invasive | 1 | 1 | A | 407 | 2 |
| 149 | MGAS26864 | United States | MN | 2003 | Invasive | 1 | 1 | B | 407 | 3 |
| 150 | MGAS26865 | United States | MN | 2003 | Invasive | 1 | 1 | B | 407 | 2 |
| 151 | MGAS26866 | United States | MN | 2003 | Invasive | 1 | 1 | B | 407 | 6 |
| 152 | MGAS26867 | United States | NY | 2003 | Invasive | 1 | 1 | A | 407 | 1 |
| 153 | MGAS26873 | United States | TN | 2010 | Invasive | 1 | 1 | A | 407 | 6 |
| 154 | MGAS26875 | United States | NY | 1997 | Invasive | 1 | 1 | A | 407 | 11 |
| 155 | MGAS26876 | United States | NY | 2002 | Invasive | 1 | 1 | A | 407 | 3 |
| 156 | MGAS26877 | United States | NY | 2002 | Invasive | 1 | 1 | A | 407 | 3 |
| 157 | MGAS26878 | United States | NY | 2002 | Invasive | 1 | 1 | B | 407 | 5 |
| 158 | MGAS26879 | United States | NY | 2002 | Invasive | 1 | 1 | B | 407 | 3 |
| 159 | MGAS26880 | United States | NY | 2002 | Invasive | 1 | 1 | A | 407 | 3 |
| 160 | MGAS26881 | United States | GA | 2003 | Invasive | 1 | 1 | A | 407 | 5 |
| 161 | MGAS26882 | United States | MD | 1998 | Invasive | 1 | 1 | A | 407 | 10 |
| 162 | MGAS26883 | United States | MD | 1998 | Invasive | 1 | 1 | A | 407 | 6 |
| 163 | MGAS26884 | United States | MD | 1998 | Invasive | 1 | 1 | A | 407 | 6 |
| 164 | MGAS26885 | United States | MD | 1998 | Invasive | 1 | 1 | A | 407 | 3 |
| 165 | MGAS26886 | United States | MD | 1998 | Invasive | 1 | 1 | A | 407 | 3 |
| 166 | MGAS26887 | United States | MN | 1998 | Invasive | 1 | 1 | A | 407 | 3 |
| 167 | MGAS26888 | United States | MN | 1998 | Invasive | 1 | 1 | A | 407 | 3 |
| 168 | MGAS26889 | United States | GA | 2003 | Invasive | 1 | 1 | A | 407 | 3 |
| 169 | MGAS26890 | United States | NY | 1998 | Invasive | 1 | 1 | A | 407 | 3 |
| 170 | MGAS26891 | United States | MN | 1998 | Invasive | 1 | 1 | A | 407 | 3 |
| 171 | MGAS26892 | United States | CT | 1998 | Invasive | 1 | 1 | A | 407 | 6 |
| 172 | MGAS26893 | United States | MN | 2003 | Invasive | 1 | 1 | A | 407 | 16 |
| 173 | MGAS26894 | United States | MN | 1997 | Invasive | 1 | 1 | A | 407 | 3 |
| 174 | MGAS26895 | United States | MN | 1997 | Invasive | 1 | 1 | A | 407 | 3 |
| 175 | MGAS26896 | United States | MN | 1997 | Invasive | 1 | 1 | A | 407 | 3 |
| 176 | MGAS26897 | United States | MN | 1997 | Invasive | 1 | 1 | A | 407 | 3 |
| 177 | MGAS26898 | United States | MN | 1997 | Invasive | 1 | 1 | A | 407 | 8 |
| 178 | MGAS26900 | United States | CT | 2003 | Invasive | 1 | 1 | B | 407 | 9 |
| 179 | MGAS26901 | United States | CT | 2003 | Invasive | 1 | 1 | B | 407 | 3 |
| 180 | MGAS26902 | United States | NY | 2003 | Invasive | 1 | 1 | A | 407 | 5 |
| 181 | MGAS26903 | United States | CO | 2003 | Invasive | 1 | 1 | B | 407 | 3 |
| 182 | MGAS26904 | United States | CO | 2003 | Invasive | 1 | 1 | B | 407 | 3 |
| 183 | MGAS26906 | United States | CO | 2002 | Invasive | 1 | 1 | B | 407 | 3 |
| 184 | MGAS26907 | United States | MN | 2003 | Invasive | 1 | 1 | B | 407 | 3 |
| 185 | MGAS26908 | United States | GA | 2000 | Invasive | 1 | 1 | A | NF | 9 |
| 186 | MGAS26909 | United States | GA | 2000 | Invasive | 1 | 1 | A | 407 | 40 |
| 187 | MGAS26910 | United States | NY | 1999 | Invasive | 1 | 1 | A | 407 | 9 |
| 188 | MGAS26911 | United States | MN | 1999 | Invasive | 1 | 1 | A | 407 | 5 |
| 189 | MGAS26912 | United States | OR | 1999 | Invasive | 1 | 1 | B | 407 | 3 |
| 190 | MGAS26913 | United States | MD | 1999 | Invasive | 1 | 1 | A | 407 | 11 |
| 191 | MGAS26914 | United States | MD | 1999 | Invasive | 1 | 1 | A | 407 | 9 |
| 192 | MGAS26917 | United States | MD | 2010 | Invasive | 1 | 1 | B | 407 | 6 |
| 193 | MGAS26918 | United States | OR | 1999 | Invasive | 1 | 1 | B | 407 | 3 |
| 194 | MGAS26919 | United States | NY | 2003 | Invasive | 1 | 1 | A | 407 | 3 |
| 195 | MGAS26921 | United States | CT | 2003 | Invasive | 1 | 1 | B | 407 | 3 |
| 196 | MGAS26922 | United States | CT | 2003 | Invasive | 1 | 1 | A | 407 | 11 |
| 197 | MGAS26924 | United States | CT | 2003 | Invasive | 1 | 1 | B | 407 | 3 |
| 198 | MGAS26925 | United States | CT | 2003 | Invasive | 1 | 1 | A | 407 | 5 |
| 199 | MGAS26926 | United States | CT | 2003 | Invasive | 1 | 1 | B | NF | 63 |
| 200 | MGAS26927 | United States | NY | 2003 | Invasive | 1 | 1 | B | 407 | 12 |
| 201 | MGAS26928 | United States | NY | 2003 | Invasive | 1 | 1 | A | 407 | 3 |
| 202 | MGAS26929 | United States | MD | 2003 | Invasive | 1 | 1 | B | 407 | 3 |
| 203 | MGAS26930 | United States | MD | 2003 | Invasive | 1 | 1 | A | 407 | 3 |
| 204 | MGAS26931 | United States | MD | 2003 | Invasive | 1 | 1 | A | 407 | 5 |
| 205 | MGAS26932 | United States | MD | 2003 | Invasive | 1 | 1 | B | 407 | 3 |
| 206 | MGAS26934 | United States | CO | 2003 | Invasive | 1 | 1 | B | 407 | 3 |
| 207 | MGAS26935 | United States | MN | 2003 | Invasive | 1 | 1 | A | 407 | 16 |
| 208 | MGAS26936 | United States | MN | 2003 | Invasive | 1 | 1 | B | 407 | 3 |
| 209 | MGAS26937 | United States | CO | 2010 | Invasive | 1 | 1 | B | 407 | 2 |
| 210 | MGAS26940 | United States | GA | 1998 | Invasive | 1 | 1 | B | 407 | 3 |
| 211 | MGAS26941 | United States | NY | 1999 | Invasive | 1 | 1 | A | 407 | 5 |
| 212 | MGAS26942 | United States | CT | 1999 | Invasive | 1 | 1 | A | NF | 11 |
| 213 | MGAS26943 | United States | CT | 1999 | Invasive | 1 | 1 | A | 407 | 13 |
| 214 | MGAS26953 | United States | CO | 2010 | Invasive | 1 | 1 | B | 407 | 6 |
| 215 | MGAS26954 | United States | MN | 2006 | Invasive | 1 | 1 | B | 407 | 5 |
| 216 | MGAS26959 | United States | MD | 2006 | Invasive | 1 | 1 | A | 407 | 2 |
| 217 | MGAS26960 | United States | TN | 2006 | Invasive | 1 | 1 | B | 407 | 6 |
| 218 | MGAS26961 | United States | TN | 2006 | Invasive | 1 | 1 | A | 407 | 5 |
| 219 | MGAS26963 | United States | NM | 2006 | Invasive | 1 | 1 | B | 407 | 6 |
| 220 | MGAS26964 | United States | NM | 2006 | Invasive | 1 | 1 | B | 407 | 6 |
| 221 | MGAS26966 | United States | NM | 2006 | Invasive | 1 | 1 | B | 407 | 10 |
| 222 | MGAS26967 | United States | CO | 2010 | Invasive | 1 | 1 | B | 407 | 3 |
| 223 | MGAS26968 | United States | MN | 2006 | Invasive | 1 | 1 | B | 407 | 10 |
| 224 | MGAS26970 | United States | MD | 2006 | Invasive | 1 | 1 | B | 407 | 3 |
| 225 | MGAS26972 | United States | CO | 2006 | Invasive | 1 | 1 | B | 407 | 3 |
| 226 | MGAS26973 | United States | NY | 2007 | Invasive | 1 | 1 | B | 407 | 3 |
| 227 | MGAS26975 | United States | MN | 2006 | Invasive | 1 | 1 | B | 407 | 3 |
| 228 | MGAS26976 | United States | GA | 2007 | Invasive | 1 | 1 | B | 407 | 6 |
| 229 | MGAS26977 | United States | GA | 2007 | Invasive | 1 | 1 | B | 407 | 3 |
| 230 | MGAS26978 | United States | CA | 2006 | Invasive | 1 | 1 | A | 407 | 5 |
| 231 | MGAS26979 | United States | GA | 2007 | Invasive | 1 | 1 | B | 407 | 9 |
| 232 | MGAS26982 | United States | MN | 2007 | Invasive | 1 | 1 | A | 407 | 9 |
| 233 | MGAS26983 | United States | CO | 2007 | Invasive | 1 | 1 | A | 407 | 53 |
| 234 | MGAS26987 | United States | TN | 2007 | Invasive | 1 | 1 | A | NF | 6 |
| 235 | MGAS26989 | United States | TN | 2007 | Invasive | 1 | 1 | A | 407 | 6 |
| 236 | MGAS26992 | United States | CO | 2007 | Invasive | 1 | 1 | A | 407 | 13 |
| 237 | MGAS26993 | United States | CO | 2007 | Invasive | 1 | 1 | B | 407 | 2 |
| 238 | MGAS26994 | United States | CO | 2007 | Invasive | 1 | 1 | B | 407 | 2 |
| 239 | MGAS26995 | United States | CO | 2007 | Invasive | 1 | 1 | B | 407 | 2 |
| 240 | MGAS26996 | United States | MD | 2007 | Invasive | 1 | 1 | B | 407 | 9 |
| 241 | MGAS26999 | United States | CA | 2007 | Invasive | 1 | 1 | A | 407 | 3 |
| 242 | MGAS27001 | United States | NY | 2007 | Invasive | 1 | 1 | A | 407 | 5 |
| 243 | MGAS27002 | United States | TN | 2007 | Invasive | 1 | 1 | A | 407 | 3 |
| 244 | MGAS27003 | United States | TN | 2007 | Invasive | 1 | 1 | A | 407 | 6 |
| 245 | MGAS27007 | United States | CO | 2007 | Invasive | 1 | 1 | B | 407 | 2 |
| 246 | MGAS27008 | United States | CO | 2007 | Invasive | 1 | 1 | B | 407 | 3 |
| 247 | MGAS27009 | United States | NM | 2007 | Invasive | 1 | 1 | A | 407 | 50 |
| 248 | MGAS27010 | United States | TN | 2007 | Invasive | 1 | 1 | A | 407 | 41 |
| 249 | MGAS27014 | United States | MD | 2008 | Invasive | 1 | 1 | B | 407 | 3 |
| 250 | MGAS27016 | United States | MN | 2001 | Invasive | 1 | 1 | A | 407 | 20 |
| 251 | MGAS27022 | United States | CO | 2008 | Invasive | 1 | 1 | B | 407 | 3 |
| 252 | MGAS27023 | United States | CO | 2008 | Invasive | 1 | 1 | A | 407 | 16 |
| 253 | MGAS27027 | United States | NY | 2008 | Invasive | 1 | 1 | B | NF | 3 |
| 254 | MGAS27029 | United States | GA | 2008 | Invasive | 1 | 1 | B | 407 | 12 |
| 255 | MGAS27031 | United States | GA | 2008 | Invasive | 1 | 1 | B | 407 | 3 |
| 256 | MGAS27032 | United States | MN | 2008 | Invasive | 1 | 1 | B | 407 | 27 |
| 257 | MGAS27033 | United States | MN | 2008 | Invasive | 1 | 1 | B | 407 | 6 |
| 258 | MGAS27034 | United States | TN | 2008 | Invasive | 1 | 1 | A | 407 | 6 |
| 259 | MGAS27035 | United States | TN | 2008 | Invasive | 1 | 1 | B | 407 | 62 |
| 260 | MGAS27036 | United States | TN | 2008 | Invasive | 1 | 1 | A | 407 | 10 |
| 261 | MGAS27037 | United States | TN | 2008 | Invasive | 1 | 1 | A | 407 | 6 |
| 262 | MGAS27039 | United States | OR | 2008 | Invasive | 1 | 1 | B | 407 | 3 |
| 263 | MGAS27040 | United States | OR | 2008 | Invasive | 1 | 1 | B | 407 | 6 |
| 264 | MGAS27043 | United States | MD | 2008 | Invasive | 1 | 1 | B | 407 | 3 |
| 265 | MGAS27046 | United States | TN | 2008 | Invasive | 1 | 1 | A | 407 | 6 |
| 266 | MGAS27048 | United States | NY | 2008 | Invasive | 1 | 1 | A | 407 | 3 |
| 267 | MGAS27050 | United States | CO | 2008 | Invasive | 1 | 1 | A | 407 | 8 |
| 268 | MGAS27052 | United States | CO | 2008 | Invasive | 1 | 1 | A | 407 | 13 |
| 269 | MGAS27056 | United States | TN | 2008 | Invasive | 1 | 1 | A | 407 | 6 |
| 270 | MGAS27058 | United States | TN | 2008 | Invasive | 1 | 1 | A | 407 | 6 |
| 271 | MGAS27059 | United States | CO | 2008 | Invasive | 1 | 1 | B | 407 | 2 |
| 272 | MGAS27065 | United States | MN | 2008 | Invasive | 1 | 1 | B | 407 | 5 |
| 273 | MGAS27066 | United States | MN | 2008 | Invasive | 1 | 1 | A | 407 | 9 |
| 274 | MGAS27067 | United States | CO | 2008 | Invasive | 1 | 1 | B | 407 | 3 |
| 275 | MGAS27068 | United States | CO | 2008 | Invasive | 1 | 1 | A | 407 | 16 |
| 276 | MGAS27088 | United States | NM | 2011 | Invasive | 1 | 1 | B | 407 | 3 |
| 277 | MGAS27091 | United States | CO | 2011 | Invasive | 1 | 1 | B | 407 | 3 |
| 278 | MGAS27118 | United States | NY | 2012 | Invasive | 1 | 1 | A | 407 | 3 |
| 279 | MGAS27124 | United States | NM | 2012 | Invasive | 1 | 1 | B | 407 | 26 |
| 280 | MGAS27132 | United States | NM | 2012 | Invasive | 1 | 1 | B | 407 | 3 |
| 281 | MGAS27141 | United States | MD | 2012 | Invasive | 1 | 1 | B | NF | 3 |
| 282 | MGAS27171 | United States | NY | 2012 | Invasive | 1 | 1 | A | 407 | 3 |
| 283 | MGAS27215 | United States | NM | 2012 | Invasive | 1 | 1 | B | 407 | 3 |
| 284 | MGAS27217 | United States | MD | 2012 | Invasive | 1 | 1 | B | 407 | 6 |
| 285 | MGAS27224 | United States | TN | 2012 | Invasive | 1 | 1 | B | 407 | 3 |
| 286 | MGAS27263 | United States | NY | 2013 | Invasive | 1 | 1 | B | 407 | 11 |
| 287 | MGAS27268 | United States | MN | 2013 | Invasive | 1 | 1 | B | 407 | 12 |
| 288 | MGAS27305 | United States | CO | 2013 | Invasive | 1 | 1 | B | 407 | 3 |
| 289 | MGAS27319 | United States | NY | 2013 | Invasive | 1 | 1 | B | 407 | 5 |
| 290 | MGAS27335 | United States | CA | 2000 | Invasive | 1 | 1 | A | 407 | 3 |
| 291 | MGAS27336 | United States | MD | 2000 | Invasive | 1 | 1 | A | 407 | 3 |
| 292 | MGAS27337 | United States | MD | 2000 | Invasive | 1 | 1 | A | 407 | 6 |
| 293 | MGAS27338 | United States | MD | 2000 | Invasive | 1 | 1 | A | 407 | 3 |
| 294 | MGAS27339 | United States | MN | 2000 | Invasive | 1 | 1 | A | 407 | 11 |
| 295 | MGAS27344 | United States | CT | 2003 | Invasive | 1 | 1 | B | 407 | 3 |
| 296 | MGAS27345 | United States | GA | 2003 | Invasive | 1 | 1 | B | 407 | 3 |
| 297 | MGAS27346 | United States | NY | 2003 | Invasive | 1 | 1 | B | 407 | 5 |
| 298 | MGAS27347 | United States | NY | 2003 | Invasive | 1 | 1 | A | 407 | 5 |
| 299 | MGAS27348 | United States | NY | 1999 | Invasive | 1 | 1 | B | 407 | 5 |
| 300 | MGAS27349 | United States | CO | 2003 | Invasive | 1 | 1 | B | 407 | 3 |
| 301 | MGAS27350 | United States | CT | 2000 | Invasive | 1 | 1 | A | 407 | 13 |
| 302 | MGAS27351 | United States | CT | 2000 | Invasive | 1 | 1 | B | NF | 2 |
| 303 | MGAS27353 | United States | NY | 2000 | Invasive | 1 | 1 | B | 407 | 3 |
| 304 | MGAS27354 | United States | NY | 2000 | Invasive | 1 | 1 | A | NF | 5 |
| 305 | MGAS27355 | United States | CA | 2003 | Invasive | 1 | 1 | A | 407 | 10 |
| 306 | MGAS27356 | United States | MN | 2003 | Invasive | 1 | 1 | B | 407 | 58 |
| 307 | MGAS27357 | United States | MN | 2003 | Invasive | 1 | 1 | B | 407 | 9 |
| 308 | MGAS27358 | United States | CT | 2003 | Invasive | 1 | 1 | B | 407 | 3 |
| 309 | MGAS27359 | United States | CT | 2003 | Invasive | 1 | 1 | B | 407 | 27 |
| 310 | MGAS27360 | United States | GA | 2003 | Invasive | 1 | 1 | A | 407 | 3 |
| 311 | MGAS27361 | United States | CO | 2004 | Invasive | 1 | 1 | B | 407 | 3 |
| 312 | MGAS27363 | United States | NY | 2004 | Invasive | 1 | 1 | B | 407 | 5 |
| 313 | MGAS27364 | United States | GA | 2000 | Invasive | 1 | 1 | A | 407 | 3 |
| 314 | MGAS27366 | United States | CO | 2004 | Invasive | 1 | 1 | B | 407 | 3 |
| 315 | MGAS27367 | United States | MN | 2004 | Invasive | 1 | 1 | A | 407 | 3 |
| 316 | MGAS27368 | United States | CT | 2004 | Invasive | 1 | 1 | B | 407 | 12 |
| 317 | MGAS27369 | United States | CT | 2004 | Invasive | 1 | 1 | B | 407 | 6 |
| 318 | MGAS27370 | United States | CT | 2004 | Invasive | 1 | 1 | B | 407 | 3 |
| 319 | MGAS27372 | United States | CT | 2000 | Invasive | 1 | 1 | A | NF | 11 |
| 320 | MGAS27373 | United States | CT | 2000 | Invasive | 1 | 1 | B | 407 | 5 |
| 321 | MGAS27375 | United States | CO | 2000 | Invasive | 1 | 1 | A | 407 | 3 |
| 322 | MGAS27377 | United States | MN | 2004 | Invasive | 1 | 1 | A | 407 | 13 |
| 323 | MGAS27378 | United States | MN | 2004 | Invasive | 1 | 1 | A | 407 | 10 |
| 324 | MGAS27379 | United States | MD | 2004 | Invasive | 1 | 1 | B | 407 | 3 |
| 325 | MGAS27380 | United States | MN | 2004 | Invasive | 1 | 1 | B | 407 | 2 |
| 326 | MGAS27381 | United States | TN | 2004 | Invasive | 1 | 1 | B | 407 | 3 |
| 327 | MGAS27382 | United States | CO | 2004 | Invasive | 1 | 1 | B | 407 | 3 |
| 328 | MGAS27383 | United States | TN | 2004 | Invasive | 1 | 1 | A | 407 | 3 |
| 329 | MGAS27384 | United States | OR | 2004 | Invasive | 1 | 1 | A | 407 | 13 |
| 330 | MGAS27385 | United States | CO | 2004 | Invasive | 1 | 1 | B | 407 | 6 |
| 331 | MGAS27394 | United States | GA | 2004 | Invasive | 1 | 1 | A | 407 | 48 |
| 332 | MGAS27395 | United States | MN | 2004 | Invasive | 1 | 1 | B | 407 | 3 |
| 333 | MGAS27396 | United States | MN | 2004 | Invasive | 1 | 1 | B | 407 | 6 |
| 334 | MGAS27398 | United States | CO | 2004 | Invasive | 1 | 1 | B | 407 | 3 |
| 335 | MGAS27401 | United States | MD | 2004 | Invasive | 1 | 1 | A | 407 | 5 |
| 336 | MGAS27403 | United States | MN | 2004 | Invasive | 1 | 1 | B | 407 | 3 |
| 337 | MGAS27404 | United States | MN | 2004 | Invasive | 1 | 1 | A | 407 | 3 |
| 338 | MGAS27405 | United States | CO | 2005 | Invasive | 1 | 1 | B | 407 | 3 |
| 339 | MGAS27406 | United States | CO | 2005 | Invasive | 1 | 1 | B | 407 | 12 |
| 340 | MGAS27409 | United States | CO | 2005 | Invasive | 1 | 1 | B | 407 | 3 |
| 341 | MGAS27414 | United States | CO | 2011 | Invasive | 1 | 1 | B | 407 | 3 |
| 342 | MGAS27416 | United States | MN | 2010 | Invasive | 1 | 1 | B | 407 | 12 |
| 343 | MGAS27417 | United States | MN | 2011 | Invasive | 1 | 1 | A | 407 | 16 |
| 344 | MGAS27424 | United States | CA | 2011 | Invasive | 1 | 1 | A | 407 | 5 |
| 345 | MGAS27427 | United States | NY | 2011 | Invasive | 1 | 1 | B | 407 | 12 |
| 346 | MGAS27438 | United States | MD | 2006 | Invasive | 1 | 1 | B | 407 | 3 |
| 347 | MGAS27439 | United States | TN | 2006 | Invasive | 1 | 1 | A | 407 | 61 |
| 348 | MGAS27440 | United States | GA | 2006 | Invasive | 1 | 1 | B | 407 | 9 |
| 349 | MGAS27441 | United States | CO | 2006 | Invasive | 1 | 1 | A | 407 | 3 |
| 350 | MGAS27442 | United States | GA | 2006 | Invasive | 1 | 1 | A | 407 | 20 |
| 351 | MGAS27444 | United States | MN | 2006 | Invasive | 1 | 1 | B | 407 | 3 |
| 352 | MGAS27445 | United States | GA | 2006 | Invasive | 1 | 1 | B | 407 | 3 |
| 353 | MGAS27446 | United States | NM | 2006 | Invasive | 1 | 1 | B | 407 | 28 |
| 354 | MGAS27448 | United States | MD | 2006 | Invasive | 1 | 1 | A | 407 | 2 |
| 355 | MGAS27454 | United States | NY | 2006 | Invasive | 1 | 1 | B | 407 | 9 |
| 356 | MGAS27455 | United States | MN | 2006 | Invasive | 1 | 1 | B | 407 | 59 |
| 357 | MGAS27456 | United States | MN | 2006 | Invasive | 1 | 1 | B | 407 | 9 |
| 358 | MGAS27773 | Iceland | -- | 2000 | Invasive | 1 | 1 | A | 407 | 9 |
| 359 | MGAS27774 | Iceland | -- | 2000 | Invasive | 1 | 1 | A | 407 | 9 |
| 360 | MGAS23530 | Italy | -- | 1997 | Pharyngitis | 2 | 2 | A | 101 | 2 |
| 361 | MGAS26583 | United States | MN | 2005 | Invasive | 2 | 2 | B | 101 | 2 |
| 362 | MGAS26594 | United States | CO | 2004 | Invasive | 2 | 2 | A | 101 | 2 |
| 363 | MGAS26597 | United States | MN | 2005 | Invasive | 2 | 2 | B | 101 | 2 |
| 364 | MGAS26609 | United States | CO | 2005 | Invasive | 2 | 2 | A | 101 | 2 |
| 365 | MGAS26659 | United States | MD | 2009 | Invasive | 2 | 2 | C | 101 | 42 |
| 366 | MGAS26663 | United States | GA | 2010 | Invasive | 2 | 2 | A | 101 | 25 |
| 367 | MGAS26682 | United States | MD | 2009 | Invasive | 2 | 2 | C | 101 | 8 |
| 368 | MGAS26698 | United States | CT | 2001 | Invasive | 2 | 2 | A | NF | 2 |
| 369 | MGAS26699 | United States | CT | 2001 | Invasive | 2 | 2 | A | 101 | 32 |
| 370 | MGAS26723 | United States | CT | 2002 | Invasive | 2 | 2 | A | 101 | 2 |
| 371 | MGAS26724 | United States | CT | 2002 | Invasive | 2 | 2 | A | 101 | 2 |
| 372 | MGAS26761 | United States | CO | 2002 | Invasive | 2 | 2 | A | 101 | 2 |
| 373 | MGAS26763 | United States | CT | 2002 | Invasive | 2 | 2 | A | 101 | 2 |
| 374 | MGAS26764 | United States | CO | 2002 | Invasive | 2 | 2 | A | 101 | 2 |
| 375 | MGAS26765 | United States | CO | 2001 | Invasive | 2 | 2 | A | 101 | 2 |
| 376 | MGAS26768 | United States | CT | 2002 | Invasive | 2 | 2 | A | 101 | 2 |
| 377 | MGAS26769 | United States | CT | 2002 | Invasive | 2 | 2 | A | 101 | 2 |
| 378 | MGAS26825 | United States | CO | 2001 | Invasive | 2 | 2 | A | 101 | 2 |
| 379 | MGAS26826 | United States | CO | 2001 | Invasive | 2 | 2 | A | 101 | 2 |
| 380 | MGAS26842 | United States | CO | 2003 | Invasive | 2 | 2 | A | 101 | 2 |
| 381 | MGAS26846 | United States | MD | 2003 | Invasive | 2 | 2 | C | 101 | 2 |
| 382 | MGAS26868 | United States | GA | 2003 | Invasive | 2 | 2 | C | 101 | 33 |
| 383 | MGAS26905 | United States | CO | 2003 | Invasive | 2 | 2 | A | 101 | 2 |
| 384 | MGAS26920 | United States | CT | 2003 | Invasive | 2 | 2 | A | 101 | 2 |
| 385 | MGAS26923 | United States | CT | 2003 | Invasive | 2 | 2 | A | 101 | 39 |
| 386 | MGAS26933 | United States | CO | 2003 | Invasive | 2 | 2 | A | 101 | 2 |
| 387 | MGAS26938 | United States | MN | 2010 | Invasive | 2 | 2 | B | 101 | 2 |
| 388 | MGAS26950 | United States | MD | 2010 | Invasive | 2 | 2 | A | 101 | 2 |
| 389 | MGAS26984 | United States | CA | 2007 | Invasive | 2 | 2 | A | 101 | 2 |
| 390 | MGAS27000 | United States | NY | 2007 | Invasive | 2 | 2 | C | 101 | 8 |
| 391 | MGAS27074 | United States | MN | 2009 | Invasive | 2 | 2 | B | 101 | 2 |
| 392 | MGAS27114 | United States | NM | 2012 | Invasive | 2 | 2 | A | NF | 25 |
| 393 | MGAS27340 | United States | CO | 2000 | Invasive | 2 | 2 | A | 101 | 2 |
| 394 | MGAS27341 | United States | CO | 2000 | Invasive | 2 | 2 | A | 101 | 2 |
| 395 | MGAS27342 | United States | CO | 2000 | Invasive | 2 | 2 | A | 101 | 2 |
| 396 | MGAS27343 | United States | CO | 2000 | Invasive | 2 | 2 | A | 101 | 2 |
| 397 | MGAS27352 | United States | CT | 2000 | Invasive | 2 | 2 | A | 101 | 2 |
| 398 | MGAS27362 | United States | CO | 2004 | Invasive | 2 | 2 | A | 101 | 2 |
| 399 | MGAS27365 | United States | CO | 2004 | Invasive | 2 | 2 | A | 101 | 2 |
| 400 | MGAS27374 | United States | CO | 2000 | Invasive | 2 | 2 | A | 101 | 2 |
| 401 | MGAS27376 | United States | CO | 2000 | Invasive | 2 | 2 | A | 101 | 2 |
| 402 | MGAS27386 | United States | CA | 2004 | Invasive | 2 | 2 | A | 101 | 10 |
| 403 | MGAS27397 | United States | CO | 2004 | Invasive | 2 | 2 | A | 101 | 2 |
| 404 | MGAS27407 | United States | CO | 2005 | Invasive | 2 | 2 | A | 101 | 2 |
| 405 | MGAS27408 | United States | CO | 2005 | Invasive | 2 | 2 | A | 101 | 2 |
| 406 | MGAS27458 | Finland | -- | 2003 | Invasive | 2 | 2 | A | 101 | 8 |
| 407 | MGAS27461 | Finland | -- | 2004 | Invasive | 2 | 2 | A | 101 | 22 |
| 408 | MGAS27464 | Finland | -- | 2004 | Invasive | 2 | 2 | A | 101 | 47 |
| 409 | MGAS27471 | Finland | -- | 2005 | Invasive | 2 | 2 | A | 101 | 45 |
| 410 | MGAS27474 | Finland | -- | 2006 | Invasive | 2 | 2 | A | 101 | 2 |
| 411 | MGAS27478 | Finland | -- | 2006 | Invasive | 2 | 2 | A | 101 | 31 |
| 412 | MGAS27484 | Finland | -- | 2007 | Invasive | 2 | 2 | A | 101 | 2 |
| 413 | MGAS27489 | Finland | -- | 2007 | Invasive | 2 | 2 | A | 101 | 8 |
| 414 | MGAS27491 | Finland | -- | 2007 | Invasive | 2 | 2 | A | 101 | 2 |
| 415 | MGAS27492 | Finland | -- | 2007 | Invasive | 2 | 2 | A | 101 | 2 |
| 416 | MGAS27506 | Finland | -- | 2009 | Invasive | 2 | 2 | A | 101 | 8 |
| 417 | MGAS27514 | Finland | -- | 2009 | Invasive | 2 | 2 | A | 101 | 8 |
| 418 | MGAS27516 | Finland | -- | 2009 | Invasive | 2 | 2 | A | 101 | 2 |
| 419 | MGAS27524 | Finland | -- | 2009 | Invasive | 2 | 2 | A | 101 | 8 |
| 420 | MGAS27545 | Finland | -- | 2010 | Invasive | 2 | 2 | A | 101 | 15 |
| 421 | MGAS27580 | Finland | -- | 2011 | Invasive | 2 | 2 | A | 101 | 1 |
| 422 | MGAS27611 | Finland | -- | 2012 | Invasive | 2 | 2 | A | 101 | 2 |
| 423 | MGAS27751 | Iceland | -- | 1997 | Invasive | 2 | 2 | A | 101 | 2 |
| 424 | MGAS27771 | Iceland | -- | 2000 | Invasive | 2 | 2 | A | 101 | 2 |
| 425 | MGAS27782 | Iceland | -- | 2001 | Invasive | 2 | 2 | A | 101 | 2 |
| 426 | MGAS27786 | Iceland | -- | 2001 | Invasive | 2 | 2 | A | 101 | 2 |
| 427 | MGAS27788 | Iceland | -- | 2001 | Invasive | 2 | 2 | A | 101 | 2 |
| 428 | MGAS27794 | Iceland | -- | 2002 | Invasive | 2 | 2 | A | 101 | 2 |
| 429 | MGAS27796 | Iceland | -- | 2002 | Invasive | 2 | 2 | A | 101 | 2 |
| 430 | MGAS27800 | Iceland | -- | 2002 | Invasive | 2 | 2 | A | 101 | 2 |
| 431 | MGAS27801 | Iceland | -- | 2003 | Invasive | 2 | 2 | A | 101 | 2 |
| 432 | MGAS27802 | Iceland | -- | 2003 | Invasive | 2 | 2 | A | 101 | 2 |
| 433 | MGAS27804 | Iceland | -- | 2003 | Invasive | 2 | 2 | A | NF | 2 |
| 434 | MGAS27806 | Iceland | -- | 2003 | Invasive | 2 | 2 | A | NF | 2 |
| 435 | MGAS27807 | Iceland | -- | 2003 | Invasive | 2 | 2 | A | 101 | 2 |
| 436 | MGAS27845 | Iceland | -- | 2007 | Invasive | 2 | 2 | A | 101 | 8 |
| 437 | MGAS27852 | Iceland | -- | 2008 | Invasive | 2 | 2 | A | 101 | 38 |
| 438 | MGAS26571 | United States | MD | 2009 | Invasive | 3 | 3 | -- | 101 | 2 |
| 439 | MGAS26572 | United States | MD | 2009 | Invasive | 3 | 3 | -- | 101 | 4 |
| 440 | MGAS26575 | United States | NY | 2010 | Invasive | 3 | 3 | -- | 101 | 1 |
| 441 | MGAS26577 | United States | NY | 2010 | Invasive | 3 | 3 | -- | 101 | 1 |
| 442 | MGAS26614 | United States | NM | 2005 | Invasive | 3 | 3 | -- | 101 | 2 |
| 443 | MGAS26616 | United States | NY | 2009 | Invasive | 3 | 3 | -- | 101 | 1 |
| 444 | MGAS26617 | United States | NY | 2009 | Invasive | 3 | 3 | -- | 101 | 1 |
| 445 | MGAS26619 | United States | TN | 2008 | Invasive | 3 | 3 | -- | 101 | 1 |
| 446 | MGAS26621 | United States | GA | 2009 | Invasive | 3 | 3 | -- | 101 | 1 |
| 447 | MGAS26622 | United States | MD | 2009 | Invasive | 3 | 3 | -- | 101 | 4 |
| 448 | MGAS26623 | United States | MD | 2009 | Invasive | 3 | 3 | -- | 101 | 4 |
| 449 | MGAS26624 | United States | MD | 2009 | Invasive | 3 | 3 | -- | 101 | 4 |
| 450 | MGAS26625 | United States | NY | 2009 | Invasive | 3 | 3 | -- | 101 | 4 |
| 451 | MGAS26626 | United States | NY | 2009 | Invasive | 3 | 3 | -- | 101 | 2 |
| 452 | MGAS26627 | United States | NY | 2009 | Invasive | 3 | 3 | -- | 101 | 1 |
| 453 | MGAS26628 | United States | TN | 2008 | Invasive | 3 | 3 | -- | 101 | 1 |
| 454 | MGAS26629 | United States | TN | 2008 | Invasive | 3 | 3 | -- | 101 | 1 |
| 455 | MGAS26630 | United States | TN | 2009 | Invasive | 3 | 3 | -- | 101 | 1 |
| 456 | MGAS26631 | United States | TN | 2009 | Invasive | 3 | 3 | -- | 101 | 2 |
| 457 | MGAS26632 | United States | TN | 2009 | Invasive | 3 | 3 | -- | 101 | 1 |
| 458 | MGAS26633 | United States | TN | 2009 | Invasive | 3 | 3 | -- | 101 | 2 |
| 459 | MGAS26634 | United States | TN | 2009 | Invasive | 3 | 3 | -- | 101 | 1 |
| 460 | MGAS26636 | United States | GA | 2009 | Invasive | 3 | 3 | -- | 101 | 1 |
| 461 | MGAS26638 | United States | NY | 2009 | Invasive | 3 | 3 | -- | 101 | 1 |
| 462 | MGAS26640 | United States | TN | 2009 | Invasive | 3 | 3 | -- | 101 | 1 |
| 463 | MGAS26641 | United States | TN | 2009 | Invasive | 3 | 3 | -- | 101 | 1 |
| 464 | MGAS26643 | United States | GA | 2009 | Invasive | 3 | 3 | -- | 101 | 14 |
| 465 | MGAS26644 | United States | MD | 2009 | Invasive | 3 | 3 | -- | 101 | 19 |
| 466 | MGAS26646 | United States | MD | 2009 | Invasive | 3 | 3 | -- | 101 | 4 |
| 467 | MGAS26647 | United States | MD | 2009 | Invasive | 3 | 3 | -- | 101 | 4 |
| 468 | MGAS26648 | United States | MD | 2009 | Invasive | 3 | 3 | -- | 101 | 14 |
| 469 | MGAS26649 | United States | MD | 2009 | Invasive | 3 | 3 | -- | 101 | 1 |
| 470 | MGAS26650 | United States | GA | 2010 | Invasive | 3 | 3 | -- | 101 | 15 |
| 471 | MGAS26651 | United States | NY | 2009 | Invasive | 3 | 3 | -- | 101 | 1 |
| 472 | MGAS26652 | United States | NM | 2009 | Invasive | 3 | 3 | -- | NF | 1 |
| 473 | MGAS26654 | United States | NY | 2009 | Invasive | 3 | 3 | -- | 101 | 1 |
| 474 | MGAS26655 | United States | CO | 2009 | Invasive | 3 | 3 | -- | 101 | 1 |
| 475 | MGAS26657 | United States | CO | 2009 | Invasive | 3 | 3 | -- | 101 | 1 |
| 476 | MGAS26660 | United States | MD | 2009 | Invasive | 3 | 3 | -- | 101 | 4 |
| 477 | MGAS26661 | United States | MD | 2009 | Invasive | 3 | 3 | -- | 101 | 4 |
| 478 | MGAS26664 | United States | CA | 2009 | Invasive | 3 | 3 | -- | 101 | 2 |
| 479 | MGAS26665 | United States | MN | 2009 | Invasive | 3 | 3 | -- | 101 | 1 |
| 480 | MGAS26670 | United States | MD | 2009 | Invasive | 3 | 3 | -- | 101 | 4 |
| 481 | MGAS26671 | United States | MD | 2009 | Invasive | 3 | 3 | -- | 101 | 4 |
| 482 | MGAS26672 | United States | MD | 2009 | Invasive | 3 | 3 | -- | 101 | 4 |
| 483 | MGAS26673 | United States | MD | 2009 | Invasive | 3 | 3 | -- | 101 | 4 |
| 484 | MGAS26674 | United States | MD | 2009 | Invasive | 3 | 3 | -- | 101 | 4 |
| 485 | MGAS26675 | United States | MD | 2009 | Invasive | 3 | 3 | -- | 101 | 2 |
| 486 | MGAS26676 | United States | TN | 2009 | Invasive | 3 | 3 | -- | 101 | 1 |
| 487 | MGAS26677 | United States | NY | 2009 | Invasive | 3 | 3 | -- | 101 | 1 |
| 488 | MGAS26678 | United States | NY | 2009 | Invasive | 3 | 3 | -- | 101 | 2 |
| 489 | MGAS26679 | United States | NY | 2009 | Invasive | 3 | 3 | -- | 101 | 1 |
| 490 | MGAS26680 | United States | GA | 2009 | Invasive | 3 | 3 | -- | 101 | 1 |
| 491 | MGAS26681 | United States | TN | 2009 | Invasive | 3 | 3 | -- | 101 | 1 |
| 492 | MGAS26683 | United States | MD | 2009 | Invasive | 3 | 3 | -- | 101 | 8 |
| 493 | MGAS26684 | United States | MD | 2009 | Invasive | 3 | 3 | -- | 101 | 4 |
| 494 | MGAS26686 | United States | NY | 2009 | Invasive | 3 | 3 | -- | 101 | 1 |
| 495 | MGAS26687 | United States | NY | 2009 | Invasive | 3 | 3 | -- | 101 | 1 |
| 496 | MGAS26688 | United States | OR | 2009 | Invasive | 3 | 3 | -- | 101 | 2 |
| 497 | MGAS26689 | United States | OR | 2009 | Invasive | 3 | 3 | -- | 101 | 2 |
| 498 | MGAS26690 | United States | CA | 2009 | Invasive | 3 | 3 | -- | 101 | 1 |
| 499 | MGAS26711 | United States | GA | 2010 | Invasive | 3 | 3 | -- | 101 | 7 |
| 500 | MGAS26712 | United States | CA | 2010 | Invasive | 3 | 3 | -- | 101 | 1 |
| 501 | MGAS26713 | United States | MD | 2010 | Invasive | 3 | 3 | -- | 101 | 4 |
| 502 | MGAS26714 | United States | GA | 2010 | Invasive | 3 | 3 | -- | 101 | 1 |
| 503 | MGAS26715 | United States | NY | 2010 | Invasive | 3 | 3 | -- | 101 | 1 |
| 504 | MGAS26716 | United States | NM | 2010 | Invasive | 3 | 3 | -- | 101 | 15 |
| 505 | MGAS26727 | United States | GA | 2010 | Invasive | 3 | 3 | -- | 101 | 1 |
| 506 | MGAS26728 | United States | GA | 2010 | Invasive | 3 | 3 | -- | 101 | 1 |
| 507 | MGAS26729 | United States | NY | 2010 | Invasive | 3 | 3 | -- | 101 | 1 |
| 508 | MGAS26730 | United States | NY | 2010 | Invasive | 3 | 3 | -- | 101 | 1 |
| 509 | MGAS26731 | United States | NY | 2010 | Invasive | 3 | 3 | -- | 101 | 17 |
| 510 | MGAS26733 | United States | MD | 2010 | Invasive | 3 | 3 | -- | 101 | 1 |
| 511 | MGAS26744 | United States | CA | 2010 | Invasive | 3 | 3 | -- | 101 | 1 |
| 512 | MGAS26745 | United States | CA | 2010 | Invasive | 3 | 3 | -- | 101 | 2 |
| 513 | MGAS26746 | United States | OR | 2010 | Invasive | 3 | 3 | -- | 101 | 4 |
| 514 | MGAS26775 | United States | CA | 2010 | Invasive | 3 | 3 | -- | 101 | 1 |
| 515 | MGAS26777 | United States | GA | 2010 | Invasive | 3 | 3 | -- | 101 | 1 |
| 516 | MGAS26778 | United States | NY | 2010 | Invasive | 3 | 3 | -- | 101 | 1 |
| 517 | MGAS26779 | United States | GA | 2010 | Invasive | 3 | 3 | -- | 101 | 1 |
| 518 | MGAS26781 | United States | MN | 2010 | Invasive | 3 | 3 | -- | 101 | 1 |
| 519 | MGAS26782 | United States | GA | 2010 | Invasive | 3 | 3 | -- | 101 | 1 |
| 520 | MGAS26783 | United States | NY | 2010 | Invasive | 3 | 3 | -- | 101 | 1 |
| 521 | MGAS26784 | United States | MD | 2010 | Invasive | 3 | 3 | -- | 101 | 4 |
| 522 | MGAS26786 | United States | CA | 2011 | Invasive | 3 | 3 | -- | 101 | 1 |
| 523 | MGAS26787 | United States | OR | 2011 | Invasive | 3 | 3 | -- | 101 | 1 |
| 524 | MGAS26788 | United States | GA | 2011 | Invasive | 3 | 3 | -- | 101 | 1 |
| 525 | MGAS26789 | United States | NM | 2011 | Invasive | 3 | 3 | -- | 101 | 1 |
| 526 | MGAS26790 | United States | NY | 2011 | Invasive | 3 | 3 | -- | 101 | 4 |
| 527 | MGAS26791 | United States | GA | 2011 | Invasive | 3 | 3 | -- | 101 | 1 |
| 528 | MGAS26792 | United States | MN | 2011 | Invasive | 3 | 3 | -- | 101 | 1 |
| 529 | MGAS26793 | United States | MN | 2011 | Invasive | 3 | 3 | -- | 101 | 1 |
| 530 | MGAS26794 | United States | GA | 2011 | Invasive | 3 | 3 | -- | 101 | 1 |
| 531 | MGAS26796 | United States | NY | 2011 | Invasive | 3 | 3 | -- | 101 | 18 |
| 532 | MGAS26797 | United States | NY | 2011 | Invasive | 3 | 3 | -- | 101 | 1 |
| 533 | MGAS26799 | United States | MN | 2011 | Invasive | 3 | 3 | -- | 101 | 22 |
| 534 | MGAS26800 | United States | MN | 2011 | Invasive | 3 | 3 | -- | 101 | 1 |
| 535 | MGAS26801 | United States | MD | 2011 | Invasive | 3 | 3 | -- | 101 | 1 |
| 536 | MGAS26802 | United States | MD | 2011 | Invasive | 3 | 3 | -- | 101 | 4 |
| 537 | MGAS26803 | United States | MD | 2011 | Invasive | 3 | 3 | -- | 101 | 1 |
| 538 | MGAS26804 | United States | MD | 2011 | Invasive | 3 | 3 | -- | 101 | 8 |
| 539 | MGAS26805 | United States | CT | 2011 | Invasive | 3 | 3 | -- | 101 | 1 |
| 540 | MGAS26806 | United States | CT | 2011 | Invasive | 3 | 3 | -- | 101 | 1 |
| 541 | MGAS26808 | United States | OR | 2011 | Invasive | 3 | 3 | -- | 101 | 2 |
| 542 | MGAS26809 | United States | TN | 2011 | Invasive | 3 | 3 | -- | 101 | 1 |
| 543 | MGAS26810 | United States | MD | 2011 | Invasive | 3 | 3 | -- | 101 | 4 |
| 544 | MGAS26811 | United States | MD | 2011 | Invasive | 3 | 3 | -- | 101 | 4 |
| 545 | MGAS26812 | United States | MD | 2011 | Invasive | 3 | 3 | -- | 101 | 4 |
| 546 | MGAS26813 | United States | MD | 2011 | Invasive | 3 | 3 | -- | 101 | 4 |
| 547 | MGAS26815 | United States | GA | 2011 | Invasive | 3 | 3 | -- | 101 | 1 |
| 548 | MGAS26816 | United States | CA | 2011 | Invasive | 3 | 3 | -- | 101 | 24 |
| 549 | MGAS26817 | United States | CA | 2011 | Invasive | 3 | 3 | -- | 101 | 7 |
| 550 | MGAS26818 | United States | GA | 2011 | Invasive | 3 | 3 | -- | 101 | 2 |
| 551 | MGAS26819 | United States | OR | 2011 | Invasive | 3 | 3 | -- | 101 | 1 |
| 552 | MGAS26820 | United States | GA | 2011 | Invasive | 3 | 3 | -- | 101 | 2 |
| 553 | MGAS26828 | United States | MN | 2011 | Invasive | 3 | 3 | -- | 101 | 1 |
| 554 | MGAS26831 | United States | CA | 2011 | Invasive | 3 | 3 | -- | 101 | 1 |
| 555 | MGAS26836 | United States | OR | 2011 | Invasive | 3 | 3 | -- | 101 | 1 |
| 556 | MGAS26837 | United States | TN | 2011 | Invasive | 3 | 3 | -- | 101 | 1 |
| 557 | MGAS26844 | United States | CA | 2003 | Invasive | 3 | 3 | -- | 101 | 4 |
| 558 | MGAS26845 | United States | NY | 2003 | Invasive | 3 | 3 | -- | 101 | 1 |
| 559 | MGAS26869 | United States | GA | 2010 | Invasive | 3 | 3 | -- | 101 | 1 |
| 560 | MGAS26870 | United States | TN | 2010 | Invasive | 3 | 3 | -- | 101 | 2 |
| 561 | MGAS26871 | United States | TN | 2010 | Invasive | 3 | 3 | -- | 101 | 1 |
| 562 | MGAS26872 | United States | TN | 2010 | Invasive | 3 | 3 | -- | 101 | 1 |
| 563 | MGAS26874 | United States | GA | 2010 | Invasive | 3 | 3 | -- | 101 | 1 |
| 564 | MGAS26899 | United States | CA | 2003 | Invasive | 3 | 3 | -- | 101 | 1 |
| 565 | MGAS26915 | United States | NY | 2010 | Invasive | 3 | 3 | -- | 101 | 1 |
| 566 | MGAS26916 | United States | MD | 2010 | Invasive | 3 | 3 | -- | 101 | 4 |
| 567 | MGAS26944 | United States | OR | 2010 | Invasive | 3 | 3 | -- | 101 | 1 |
| 568 | MGAS26945 | United States | TN | 2010 | Invasive | 3 | 3 | -- | 101 | 1 |
| 569 | MGAS26946 | United States | TN | 2010 | Invasive | 3 | 3 | -- | 101 | 1 |
| 570 | MGAS26947 | United States | TN | 2010 | Invasive | 3 | 3 | -- | 101 | 1 |
| 571 | MGAS26948 | United States | TN | 2010 | Invasive | 3 | 3 | -- | 101 | 1 |
| 572 | MGAS26949 | United States | GA | 2010 | Invasive | 3 | 3 | -- | 101 | 1 |
| 573 | MGAS26951 | United States | MD | 2010 | Invasive | 3 | 3 | -- | 101 | 1 |
| 574 | MGAS26952 | United States | MD | 2010 | Invasive | 3 | 3 | -- | 101 | 4 |
| 575 | MGAS26955 | United States | CO | 2010 | Invasive | 3 | 3 | -- | 101 | 1 |
| 576 | MGAS26962 | United States | CA | 2006 | Invasive | 3 | 3 | -- | 101 | 1 |
| 577 | MGAS26969 | United States | NM | 2010 | Invasive | 3 | 3 | -- | 101 | 1 |
| 578 | MGAS26974 | United States | NY | 2007 | Invasive | 3 | 3 | -- | 101 | 1 |
| 579 | MGAS26980 | United States | NM | 2007 | Invasive | 3 | 3 | -- | 101 | 1 |
| 580 | MGAS26981 | United States | TN | 2007 | Invasive | 3 | 3 | -- | 101 | 2 |
| 581 | MGAS26985 | United States | MD | 2007 | Invasive | 3 | 3 | -- | NF | 4 |
| 582 | MGAS26986 | United States | TN | 2007 | Invasive | 3 | 3 | -- | 101 | 2 |
| 583 | MGAS26988 | United States | TN | 2007 | Invasive | 3 | 3 | -- | 101 | 2 |
| 584 | MGAS26990 | United States | NM | 2007 | Invasive | 3 | 3 | -- | 101 | 2 |
| 585 | MGAS26991 | United States | NM | 2007 | Invasive | 3 | 3 | -- | 101 | 1 |
| 586 | MGAS26997 | United States | MD | 2007 | Invasive | 3 | 3 | -- | 101 | 4 |
| 587 | MGAS26998 | United States | MD | 2007 | Invasive | 3 | 3 | -- | 101 | 4 |
| 588 | MGAS27004 | United States | MN | 2007 | Invasive | 3 | 3 | -- | 101 | 1 |
| 589 | MGAS27005 | United States | MN | 2007 | Invasive | 3 | 3 | -- | 101 | 1 |
| 590 | MGAS27006 | United States | TN | 2007 | Invasive | 3 | 3 | -- | 101 | 1 |
| 591 | MGAS27011 | United States | MD | 2007 | Invasive | 3 | 3 | -- | 101 | 4 |
| 592 | MGAS27012 | United States | MD | 2007 | Invasive | 3 | 3 | -- | 101 | 4 |
| 593 | MGAS27013 | United States | MD | 2008 | Invasive | 3 | 3 | -- | 101 | 1 |
| 594 | MGAS27015 | United States | MD | 2008 | Invasive | 3 | 3 | -- | 101 | 1 |
| 595 | MGAS27017 | United States | GA | 2008 | Invasive | 3 | 3 | -- | 101 | 2 |
| 596 | MGAS27018 | United States | CA | 2008 | Invasive | 3 | 3 | -- | NF | 4 |
| 597 | MGAS27019 | United States | TN | 2008 | Invasive | 3 | 3 | -- | 101 | 1 |
| 598 | MGAS27020 | United States | NY | 2008 | Invasive | 3 | 3 | -- | 101 | 1 |
| 599 | MGAS27021 | United States | NY | 2008 | Invasive | 3 | 3 | -- | 101 | 1 |
| 600 | MGAS27024 | United States | CA | 2008 | Invasive | 3 | 3 | -- | 101 | 1 |
| 601 | MGAS27025 | United States | CA | 2008 | Invasive | 3 | 3 | -- | 101 | 1 |
| 602 | MGAS27026 | United States | GA | 2008 | Invasive | 3 | 3 | -- | 101 | 2 |
| 603 | MGAS27028 | United States | OR | 2008 | Invasive | 3 | 3 | -- | 101 | 1 |
| 604 | MGAS27030 | United States | GA | 2008 | Invasive | 3 | 3 | -- | 101 | 1 |
| 605 | MGAS27038 | United States | OR | 2008 | Invasive | 3 | 3 | -- | 101 | 1 |
| 606 | MGAS27041 | United States | MD | 2008 | Invasive | 3 | 3 | -- | 101 | 4 |
| 607 | MGAS27042 | United States | NY | 2008 | Invasive | 3 | 3 | -- | 101 | 2 |
| 608 | MGAS27044 | United States | MD | 2008 | Invasive | 3 | 3 | -- | 101 | 4 |
| 609 | MGAS27045 | United States | MD | 2008 | Invasive | 3 | 3 | -- | 101 | 56 |
| 610 | MGAS27047 | United States | NY | 2008 | Invasive | 3 | 3 | -- | 101 | 4 |
| 611 | MGAS27049 | United States | GA | 2008 | Invasive | 3 | 3 | -- | 101 | 1 |
| 612 | MGAS27051 | United States | CO | 2008 | Invasive | 3 | 3 | -- | 101 | 1 |
| 613 | MGAS27053 | United States | NY | 2008 | Invasive | 3 | 3 | -- | 101 | 1 |
| 614 | MGAS27054 | United States | MD | 2008 | Invasive | 3 | 3 | -- | 101 | 4 |
| 615 | MGAS27055 | United States | TN | 2008 | Invasive | 3 | 3 | -- | 101 | 1 |
| 616 | MGAS27057 | United States | TN | 2008 | Invasive | 3 | 3 | -- | 101 | 1 |
| 617 | MGAS27060 | United States | CO | 2008 | Invasive | 3 | 3 | -- | 101 | 1 |
| 618 | MGAS27061 | United States | GA | 2008 | Invasive | 3 | 3 | -- | 101 | 1 |
| 619 | MGAS27062 | United States | NM | 2008 | Invasive | 3 | 3 | -- | 101 | 1 |
| 620 | MGAS27063 | United States | TN | 2008 | Invasive | 3 | 3 | -- | 101 | 1 |
| 621 | MGAS27064 | United States | GA | 2009 | Invasive | 3 | 3 | -- | NF | 18 |
| 622 | MGAS27070 | United States | NM | 2008 | Invasive | 3 | 3 | -- | 101 | 1 |
| 623 | MGAS27071 | United States | NY | 2008 | Invasive | 3 | 3 | -- | 101 | 1 |
| 624 | MGAS27072 | United States | NY | 2008 | Invasive | 3 | 3 | -- | 101 | 1 |
| 625 | MGAS27073 | United States | MN | 2008 | Invasive | 3 | 3 | -- | 101 | 1 |
| 626 | MGAS27075 | United States | MD | 2008 | Invasive | 3 | 3 | -- | 101 | 4 |
| 627 | MGAS27076 | United States | MD | 2008 | Invasive | 3 | 3 | -- | NF | 8 |
| 628 | MGAS27077 | United States | MD | 2009 | Invasive | 3 | 3 | -- | 101 | 4 |
| 629 | MGAS27078 | United States | NY | 2011 | Invasive | 3 | 3 | -- | 101 | 18 |
| 630 | MGAS27079 | United States | MN | 2011 | Invasive | 3 | 3 | -- | 101 | 1 |
| 631 | MGAS27080 | United States | MN | 2011 | Invasive | 3 | 3 | -- | 101 | 1 |
| 632 | MGAS27081 | United States | GA | 2011 | Invasive | 3 | 3 | -- | 101 | 2 |
| 633 | MGAS27082 | United States | OR | 2011 | Invasive | 3 | 3 | -- | 101 | 2 |
| 634 | MGAS27083 | United States | CA | 2011 | Invasive | 3 | 3 | -- | 101 | 1 |
| 635 | MGAS27084 | United States | CA | 2011 | Invasive | 3 | 3 | -- | 101 | 1 |
| 636 | MGAS27085 | United States | GA | 2011 | Invasive | 3 | 3 | -- | 101 | 1 |
| 637 | MGAS27086 | United States | TN | 2011 | Invasive | 3 | 3 | -- | 101 | 1 |
| 638 | MGAS27087 | United States | OR | 2011 | Invasive | 3 | 3 | -- | 101 | 1 |
| 639 | MGAS27089 | United States | NY | 2011 | Invasive | 3 | 3 | -- | 101 | 18 |
| 640 | MGAS27090 | United States | GA | 2011 | Invasive | 3 | 3 | -- | 101 | 2 |
| 641 | MGAS27092 | United States | NM | 2011 | Invasive | 3 | 3 | -- | 101 | 1 |
| 642 | MGAS27093 | United States | NM | 2011 | Invasive | 3 | 3 | -- | 101 | 1 |
| 643 | MGAS27094 | United States | NM | 2011 | Invasive | 3 | 3 | -- | 101 | 2 |
| 644 | MGAS27095 | United States | GA | 2011 | Invasive | 3 | 3 | -- | 101 | 17 |
| 645 | MGAS27096 | United States | MN | 2011 | Invasive | 3 | 3 | -- | 101 | 1 |
| 646 | MGAS27097 | United States | GA | 2012 | Invasive | 3 | 3 | -- | 101 | 1 |
| 647 | MGAS27098 | United States | TN | 2011 | Invasive | 3 | 3 | -- | 101 | 2 |
| 648 | MGAS27099 | United States | TN | 2011 | Invasive | 3 | 3 | -- | 101 | 1 |
| 649 | MGAS27100 | United States | NM | 2011 | Invasive | 3 | 3 | -- | 101 | 1 |
| 650 | MGAS27101 | United States | MD | 2011 | Invasive | 3 | 3 | -- | 101 | 4 |
| 651 | MGAS27102 | United States | MN | 2011 | Invasive | 3 | 3 | -- | 101 | 1 |
| 652 | MGAS27103 | United States | MN | 2012 | Invasive | 3 | 3 | -- | 101 | 1 |
| 653 | MGAS27104 | United States | NY | 2012 | Invasive | 3 | 3 | -- | 101 | 1 |
| 654 | MGAS27105 | United States | NY | 2012 | Invasive | 3 | 3 | -- | 101 | 1 |
| 655 | MGAS27106 | United States | CO | 2011 | Invasive | 3 | 3 | -- | NF | 2 |
| 656 | MGAS27107 | United States | NM | 2011 | Invasive | 3 | 3 | -- | 101 | 1 |
| 657 | MGAS27108 | United States | MD | 2011 | Invasive | 3 | 3 | -- | 101 | 1 |
| 658 | MGAS27109 | United States | MD | 2011 | Invasive | 3 | 3 | -- | 101 | 8 |
| 659 | MGAS27110 | United States | OR | 2012 | Invasive | 3 | 3 | -- | 101 | 1 |
| 660 | MGAS27111 | United States | OR | 2012 | Invasive | 3 | 3 | -- | 101 | 1 |
| 661 | MGAS27112 | United States | OR | 2012 | Invasive | 3 | 3 | -- | 101 | 2 |
| 662 | MGAS27113 | United States | NM | 2012 | Invasive | 3 | 3 | -- | 101 | 1 |
| 663 | MGAS27115 | United States | TN | 2011 | Invasive | 3 | 3 | -- | 101 | 1 |
| 664 | MGAS27116 | United States | TN | 2011 | Invasive | 3 | 3 | -- | 101 | 2 |
| 665 | MGAS27117 | United States | TN | 2012 | Invasive | 3 | 3 | -- | 101 | 1 |
| 666 | MGAS27119 | United States | CA | 2012 | Invasive | 3 | 3 | -- | 101 | 1 |
| 667 | MGAS27120 | United States | CA | 2012 | Invasive | 3 | 3 | -- | 101 | 1 |
| 668 | MGAS27121 | United States | CA | 2012 | Invasive | 3 | 3 | -- | 101 | 1 |
| 669 | MGAS27122 | United States | GA | 2012 | Invasive | 3 | 3 | -- | 101 | 1 |
| 670 | MGAS27123 | United States | GA | 2012 | Invasive | 3 | 3 | -- | 654 | 1 |
| 671 | MGAS27125 | United States | OR | 2012 | Invasive | 3 | 3 | -- | 101 | 1 |
| 672 | MGAS27126 | United States | GA | 2012 | Invasive | 3 | 3 | -- | 101 | 17 |
| 673 | MGAS27127 | United States | GA | 2012 | Invasive | 3 | 3 | -- | 101 | 1 |
| 674 | MGAS27128 | United States | CO | 2012 | Invasive | 3 | 3 | -- | 101 | 1 |
| 675 | MGAS27129 | United States | NY | 2012 | Invasive | 3 | 3 | -- | 101 | 1 |
| 676 | MGAS27130 | United States | GA | 2012 | Invasive | 3 | 3 | -- | 101 | 1 |
| 677 | MGAS27131 | United States | GA | 2012 | Invasive | 3 | 3 | -- | 101 | 1 |
| 678 | MGAS27133 | United States | NM | 2012 | Invasive | 3 | 3 | -- | 101 | 1 |
| 679 | MGAS27134 | United States | TN | 2012 | Invasive | 3 | 3 | -- | 101 | 1 |
| 680 | MGAS27135 | United States | TN | 2012 | Invasive | 3 | 3 | -- | 101 | 1 |
| 681 | MGAS27136 | United States | NY | 2012 | Invasive | 3 | 3 | -- | 101 | 1 |
| 682 | MGAS27137 | United States | NY | 2012 | Invasive | 3 | 3 | -- | 101 | 1 |
| 683 | MGAS27138 | United States | NY | 2012 | Invasive | 3 | 3 | -- | 101 | 1 |
| 684 | MGAS27139 | United States | MD | 2012 | Invasive | 3 | 3 | -- | 101 | 24 |
| 685 | MGAS27140 | United States | MD | 2012 | Invasive | 3 | 3 | -- | 101 | 1 |
| 686 | MGAS27142 | United States | MD | 2012 | Invasive | 3 | 3 | -- | NF | 4 |
| 687 | MGAS27143 | United States | MD | 2012 | Invasive | 3 | 3 | -- | 101 | 4 |
| 688 | MGAS27144 | United States | GA | 2012 | Invasive | 3 | 3 | -- | 101 | 2 |
| 689 | MGAS27145 | United States | GA | 2012 | Invasive | 3 | 3 | -- | 101 | 1 |
| 690 | MGAS27146 | United States | CA | 2012 | Invasive | 3 | 3 | -- | 101 | 43 |
| 691 | MGAS27147 | United States | CA | 2012 | Invasive | 3 | 3 | -- | 101 | 14 |
| 692 | MGAS27148 | United States | MN | 2012 | Invasive | 3 | 3 | -- | 101 | 1 |
| 693 | MGAS27149 | United States | MN | 2012 | Invasive | 3 | 3 | -- | NF | 1 |
| 694 | MGAS27150 | United States | GA | 2012 | Invasive | 3 | 3 | -- | NF | 1 |
| 695 | MGAS27151 | United States | GA | 2012 | Invasive | 3 | 3 | -- | 101 | 1 |
| 696 | MGAS27152 | United States | OR | 2012 | Invasive | 3 | 3 | -- | 101 | 1 |
| 697 | MGAS27153 | United States | NY | 2012 | Invasive | 3 | 3 | -- | 101 | 1 |
| 698 | MGAS27154 | United States | NY | 2012 | Invasive | 3 | 3 | -- | 101 | 1 |
| 699 | MGAS27155 | United States | GA | 2012 | Invasive | 3 | 3 | -- | 101 | 1 |
| 700 | MGAS27156 | United States | CO | 2012 | Invasive | 3 | 3 | -- | 101 | 1 |
| 701 | MGAS27157 | United States | CO | 2012 | Invasive | 3 | 3 | -- | 101 | 1 |
| 702 | MGAS27158 | United States | GA | 2012 | Invasive | 3 | 3 | -- | 101 | 1 |
| 703 | MGAS27159 | United States | GA | 2012 | Invasive | 3 | 3 | -- | NF | 4 |
| 704 | MGAS27160 | United States | TN | 2012 | Invasive | 3 | 3 | -- | 101 | 1 |
| 705 | MGAS27161 | United States | TN | 2012 | Invasive | 3 | 3 | -- | 101 | 1 |
| 706 | MGAS27162 | United States | OR | 2012 | Invasive | 3 | 3 | -- | 101 | 1 |
| 707 | MGAS27163 | United States | OR | 2012 | Invasive | 3 | 3 | -- | 101 | 1 |
| 708 | MGAS27164 | United States | NY | 2012 | Invasive | 3 | 3 | -- | 101 | 1 |
| 709 | MGAS27165 | United States | NY | 2012 | Invasive | 3 | 3 | -- | 101 | 1 |
| 710 | MGAS27166 | United States | NY | 2012 | Invasive | 3 | 3 | -- | 101 | 1 |
| 711 | MGAS27167 | United States | NY | 2012 | Invasive | 3 | 3 | -- | 101 | 1 |
| 712 | MGAS27168 | United States | GA | 2012 | Invasive | 3 | 3 | -- | 101 | 1 |
| 713 | MGAS27169 | United States | GA | 2012 | Invasive | 3 | 3 | -- | 101 | 17 |
| 714 | MGAS27170 | United States | MD | 2012 | Invasive | 3 | 3 | -- | 101 | 4 |
| 715 | MGAS27172 | United States | NM | 2012 | Invasive | 3 | 3 | -- | 101 | 2 |
| 716 | MGAS27173 | United States | GA | 2012 | Invasive | 3 | 3 | -- | 101 | 1 |
| 717 | MGAS27176 | United States | CO | 2012 | Invasive | 3 | 3 | -- | 101 | 2 |
| 718 | MGAS27177 | United States | MN | 2012 | Invasive | 3 | 3 | -- | 101 | 1 |
| 719 | MGAS27178 | United States | NM | 2012 | Invasive | 3 | 3 | -- | 101 | 1 |
| 720 | MGAS27179 | United States | CA | 2012 | Invasive | 3 | 3 | -- | 101 | 8 |
| 721 | MGAS27180 | United States | OR | 2012 | Invasive | 3 | 3 | -- | 101 | 1 |
| 722 | MGAS27181 | United States | OR | 2012 | Invasive | 3 | 3 | -- | 101 | 1 |
| 723 | MGAS27182 | United States | TN | 2012 | Invasive | 3 | 3 | -- | 101 | 1 |
| 724 | MGAS27183 | United States | TN | 2012 | Invasive | 3 | 3 | -- | 101 | 2 |
| 725 | MGAS27184 | United States | TN | 2012 | Invasive | 3 | 3 | -- | 101 | 1 |
| 726 | MGAS27185 | United States | TN | 2012 | Invasive | 3 | 3 | -- | 101 | 1 |
| 727 | MGAS27186 | United States | TN | 2012 | Invasive | 3 | 3 | -- | 101 | 2 |
| 728 | MGAS27187 | United States | TN | 2012 | Invasive | 3 | 3 | -- | 101 | 2 |
| 729 | MGAS27188 | United States | TN | 2012 | Invasive | 3 | 3 | -- | NF | 1 |
| 730 | MGAS27189 | United States | TN | 2012 | Invasive | 3 | 3 | -- | 101 | 2 |
| 731 | MGAS27190 | United States | NY | 2012 | Invasive | 3 | 3 | -- | 101 | 1 |
| 732 | MGAS27191 | United States | MN | 2012 | Invasive | 3 | 3 | -- | 101 | 7 |
| 733 | MGAS27192 | United States | GA | 2012 | Invasive | 3 | 3 | -- | NF | 1 |
| 734 | MGAS27193 | United States | CO | 2012 | Invasive | 3 | 3 | -- | 101 | 1 |
| 735 | MGAS27194 | United States | CA | 2012 | Invasive | 3 | 3 | -- | NF | 1 |
| 736 | MGAS27195 | United States | CA | 2012 | Invasive | 3 | 3 | -- | 101 | 1 |
| 737 | MGAS27196 | United States | CA | 2012 | Invasive | 3 | 3 | -- | 101 | 2 |
| 738 | MGAS27197 | United States | CA | 2012 | Invasive | 3 | 3 | -- | 101 | 7 |
| 739 | MGAS27198 | United States | TN | 2012 | Invasive | 3 | 3 | -- | 101 | 7 |
| 740 | MGAS27199 | United States | TN | 2012 | Invasive | 3 | 3 | -- | 101 | 1 |
| 741 | MGAS27200 | United States | TN | 2012 | Invasive | 3 | 3 | -- | 101 | 1 |
| 742 | MGAS27201 | United States | NM | 2012 | Invasive | 3 | 3 | -- | 101 | 2 |
| 743 | MGAS27202 | United States | NM | 2012 | Invasive | 3 | 3 | -- | NF | 2 |
| 744 | MGAS27203 | United States | NM | 2012 | Invasive | 3 | 3 | -- | 101 | 1 |
| 745 | MGAS27204 | United States | NM | 2012 | Invasive | 3 | 3 | -- | 101 | 60 |
| 746 | MGAS27205 | United States | MD | 2012 | Invasive | 3 | 3 | -- | 101 | 4 |
| 747 | MGAS27206 | United States | GA | 2012 | Invasive | 3 | 3 | -- | 101 | 1 |
| 748 | MGAS27207 | United States | MN | 2012 | Invasive | 3 | 3 | -- | 101 | 1 |
| 749 | MGAS27208 | United States | MN | 2012 | Invasive | 3 | 3 | -- | 101 | 1 |
| 750 | MGAS27209 | United States | MN | 2012 | Invasive | 3 | 3 | -- | 101 | 1 |
| 751 | MGAS27210 | United States | MN | 2012 | Invasive | 3 | 3 | -- | 101 | 7 |
| 752 | MGAS27211 | United States | MN | 2012 | Invasive | 3 | 3 | -- | 101 | 1 |
| 753 | MGAS27212 | United States | MN | 2012 | Invasive | 3 | 3 | -- | 101 | 1 |
| 754 | MGAS27213 | United States | GA | 2012 | Invasive | 3 | 3 | -- | 101 | 1 |
| 755 | MGAS27214 | United States | GA | 2012 | Invasive | 3 | 3 | -- | 101 | 1 |
| 756 | MGAS27216 | United States | NM | 2012 | Invasive | 3 | 3 | -- | 101 | 1 |
| 757 | MGAS27218 | United States | MD | 2012 | Invasive | 3 | 3 | -- | 101 | 4 |
| 758 | MGAS27219 | United States | GA | 2013 | Invasive | 3 | 3 | -- | 101 | 2 |
| 759 | MGAS27220 | United States | CA | 2012 | Invasive | 3 | 3 | -- | 101 | 14 |
| 760 | MGAS27221 | United States | GA | 2013 | Invasive | 3 | 3 | -- | 101 | 2 |
| 761 | MGAS27222 | United States | OR | 2012 | Invasive | 3 | 3 | -- | 101 | 1 |
| 762 | MGAS27223 | United States | TN | 2012 | Invasive | 3 | 3 | -- | 101 | 1 |
| 763 | MGAS27225 | United States | TN | 2012 | Invasive | 3 | 3 | -- | 101 | 1 |
| 764 | MGAS27226 | United States | TN | 2012 | Invasive | 3 | 3 | -- | 101 | 1 |
| 765 | MGAS27227 | United States | GA | 2013 | Invasive | 3 | 3 | -- | 101 | 7 |
| 766 | MGAS27228 | United States | GA | 2013 | Invasive | 3 | 3 | -- | 101 | 1 |
| 767 | MGAS27229 | United States | GA | 2013 | Invasive | 3 | 3 | -- | NF | 1 |
| 768 | MGAS27231 | United States | MN | 2013 | Invasive | 3 | 3 | -- | 101 | 1 |
| 769 | MGAS27232 | United States | MN | 2013 | Invasive | 3 | 3 | -- | 101 | 1 |
| 770 | MGAS27233 | United States | NM | 2012 | Invasive | 3 | 3 | -- | 101 | 1 |
| 771 | MGAS27234 | United States | NM | 2012 | Invasive | 3 | 3 | -- | 101 | 1 |
| 772 | MGAS27235 | United States | NM | 2012 | Invasive | 3 | 3 | -- | 101 | 17 |
| 773 | MGAS27236 | United States | OR | 2013 | Invasive | 3 | 3 | -- | 101 | 1 |
| 774 | MGAS27237 | United States | OR | 2013 | Invasive | 3 | 3 | -- | 101 | 2 |
| 775 | MGAS27238 | United States | OR | 2013 | Invasive | 3 | 3 | -- | 101 | 1 |
| 776 | MGAS27239 | United States | NY | 2013 | Invasive | 3 | 3 | -- | 101 | 1 |
| 777 | MGAS27240 | United States | GA | 2013 | Invasive | 3 | 3 | -- | 101 | 1 |
| 778 | MGAS27241 | United States | CA | 2013 | Invasive | 3 | 3 | -- | 101 | 14 |
| 779 | MGAS27242 | United States | CA | 2013 | Invasive | 3 | 3 | -- | 101 | 2 |
| 780 | MGAS27243 | United States | MD | 2013 | Invasive | 3 | 3 | -- | 101 | 4 |
| 781 | MGAS27244 | United States | MD | 2013 | Invasive | 3 | 3 | -- | 101 | 1 |
| 782 | MGAS27245 | United States | TN | 2013 | Invasive | 3 | 3 | -- | 101 | 1 |
| 783 | MGAS27246 | United States | TN | 2013 | Invasive | 3 | 3 | -- | 101 | 1 |
| 784 | MGAS27247 | United States | TN | 2013 | Invasive | 3 | 3 | -- | 101 | 19 |
| 785 | MGAS27248 | United States | TN | 2013 | Invasive | 3 | 3 | -- | 101 | 1 |
| 786 | MGAS27249 | United States | TN | 2013 | Invasive | 3 | 3 | -- | 101 | 1 |
| 787 | MGAS27250 | United States | TN | 2013 | Invasive | 3 | 3 | -- | 101 | 1 |
| 788 | MGAS27251 | United States | TN | 2013 | Invasive | 3 | 3 | -- | 101 | 1 |
| 789 | MGAS27252 | United States | TN | 2013 | Invasive | 3 | 3 | -- | 101 | 1 |
| 790 | MGAS27253 | United States | TN | 2012 | Invasive | 3 | 3 | -- | 101 | 2 |
| 791 | MGAS27254 | United States | TN | 2012 | Invasive | 3 | 3 | -- | 101 | 1 |
| 792 | MGAS27255 | United States | TN | 2012 | Invasive | 3 | 3 | -- | 101 | 1 |
| 793 | MGAS27256 | United States | GA | 2013 | Invasive | 3 | 3 | -- | 101 | 1 |
| 794 | MGAS27257 | United States | NM | 2013 | Invasive | 3 | 3 | -- | 101 | 1 |
| 795 | MGAS27258 | United States | OR | 2013 | Invasive | 3 | 3 | -- | 101 | 1 |
| 796 | MGAS27259 | United States | CO | 2013 | Invasive | 3 | 3 | -- | 101 | 7 |
| 797 | MGAS27260 | United States | NM | 2013 | Invasive | 3 | 3 | -- | 101 | 2 |
| 798 | MGAS27261 | United States | NM | 2013 | Invasive | 3 | 3 | -- | 101 | 1 |
| 799 | MGAS27262 | United States | NY | 2013 | Invasive | 3 | 3 | -- | 101 | 1 |
| 800 | MGAS27264 | United States | OR | 2013 | Invasive | 3 | 3 | -- | 101 | 2 |
| 801 | MGAS27265 | United States | OR | 2013 | Invasive | 3 | 3 | -- | 101 | 1 |
| 802 | MGAS27266 | United States | MN | 2013 | Invasive | 3 | 3 | -- | 101 | 7 |
| 803 | MGAS27267 | United States | MN | 2013 | Invasive | 3 | 3 | -- | 101 | 1 |
| 804 | MGAS27270 | United States | MN | 2013 | Invasive | 3 | 3 | -- | 101 | 1 |
| 805 | MGAS27271 | United States | GA | 2013 | Invasive | 3 | 3 | -- | 101 | 2 |
| 806 | MGAS27272 | United States | CA | 2013 | Invasive | 3 | 3 | -- | 101 | 2 |
| 807 | MGAS27273 | United States | CA | 2013 | Invasive | 3 | 3 | -- | 101 | 1 |
| 808 | MGAS27274 | United States | CA | 2013 | Invasive | 3 | 3 | -- | 101 | 1 |
| 809 | MGAS27275 | United States | NY | 2013 | Invasive | 3 | 3 | -- | 101 | 1 |
| 810 | MGAS27276 | United States | MD | 2013 | Invasive | 3 | 3 | -- | 101 | 4 |
| 811 | MGAS27277 | United States | MD | 2013 | Invasive | 3 | 3 | -- | 101 | 1 |
| 812 | MGAS27278 | United States | GA | 2013 | Invasive | 3 | 3 | -- | 101 | 1 |
| 813 | MGAS27279 | United States | TN | 2013 | Invasive | 3 | 3 | -- | 101 | 7 |
| 814 | MGAS27280 | United States | TN | 2013 | Invasive | 3 | 3 | -- | 101 | 1 |
| 815 | MGAS27281 | United States | TN | 2013 | Invasive | 3 | 3 | -- | 101 | 1 |
| 816 | MGAS27282 | United States | MN | 2013 | Invasive | 3 | 3 | -- | 101 | 7 |
| 817 | MGAS27283 | United States | MN | 2013 | Invasive | 3 | 3 | -- | 101 | 1 |
| 818 | MGAS27284 | United States | MN | 2013 | Invasive | 3 | 3 | -- | 101 | 1 |
| 819 | MGAS27285 | United States | MN | 2013 | Invasive | 3 | 3 | -- | 101 | 1 |
| 820 | MGAS27286 | United States | MN | 2013 | Invasive | 3 | 3 | -- | 101 | 1 |
| 821 | MGAS27287 | United States | GA | 2013 | Invasive | 3 | 3 | -- | 101 | 1 |
| 822 | MGAS27288 | United States | NM | 2013 | Invasive | 3 | 3 | -- | 101 | 1 |
| 823 | MGAS27289 | United States | NM | 2013 | Invasive | 3 | 3 | -- | 101 | 2 |
| 824 | MGAS27290 | United States | NY | 2013 | Invasive | 3 | 3 | -- | 580 | 1 |
| 825 | MGAS27291 | United States | NY | 2013 | Invasive | 3 | 3 | -- | 580 | 1 |
| 826 | MGAS27292 | United States | NY | 2013 | Invasive | 3 | 3 | -- | 101 | 1 |
| 827 | MGAS27293 | United States | NY | 2013 | Invasive | 3 | 3 | -- | 101 | 7 |
| 828 | MGAS27294 | United States | OR | 2013 | Invasive | 3 | 3 | -- | NF | 1 |
| 829 | MGAS27295 | United States | GA | 2013 | Invasive | 3 | 3 | -- | 101 | 1 |
| 830 | MGAS27296 | United States | GA | 2013 | Invasive | 3 | 3 | -- | 101 | 1 |
| 831 | MGAS27297 | United States | MD | 2013 | Invasive | 3 | 3 | -- | 101 | 4 |
| 832 | MGAS27298 | United States | MD | 2013 | Invasive | 3 | 3 | -- | 101 | 19 |
| 833 | MGAS27299 | United States | OR | 2013 | Invasive | 3 | 3 | -- | 101 | 1 |
| 834 | MGAS27300 | United States | TN | 2013 | Invasive | 3 | 3 | -- | 101 | 1 |
| 835 | MGAS27301 | United States | TN | 2013 | Invasive | 3 | 3 | -- | 101 | 4 |
| 836 | MGAS27302 | United States | TN | 2013 | Invasive | 3 | 3 | -- | 101 | 1 |
| 837 | MGAS27303 | United States | TN | 2013 | Invasive | 3 | 3 | -- | 101 | 1 |
| 838 | MGAS27304 | United States | TN | 2013 | Invasive | 3 | 3 | -- | 101 | 1 |
| 839 | MGAS27306 | United States | CO | 2013 | Invasive | 3 | 3 | -- | 101 | 1 |
| 840 | MGAS27307 | United States | GA | 2013 | Invasive | 3 | 3 | -- | 101 | 1 |
| 841 | MGAS27308 | United States | GA | 2013 | Invasive | 3 | 3 | -- | 101 | 1 |
| 842 | MGAS27309 | United States | CA | 2013 | Invasive | 3 | 3 | -- | 101 | 1 |
| 843 | MGAS27310 | United States | NM | 2013 | Invasive | 3 | 3 | -- | 101 | 1 |
| 844 | MGAS27311 | United States | GA | 2013 | Invasive | 3 | 3 | -- | 101 | 2 |
| 845 | MGAS27312 | United States | MN | 2013 | Invasive | 3 | 3 | -- | 101 | 1 |
| 846 | MGAS27313 | United States | MN | 2013 | Invasive | 3 | 3 | -- | 101 | 1 |
| 847 | MGAS27314 | United States | MN | 2013 | Invasive | 3 | 3 | -- | 101 | 2 |
| 848 | MGAS27315 | United States | NY | 2013 | Invasive | 3 | 3 | -- | 580 | 1 |
| 849 | MGAS27316 | United States | GA | 2013 | Invasive | 3 | 3 | -- | 101 | 1 |
| 850 | MGAS27317 | United States | CO | 2013 | Invasive | 3 | 3 | -- | 101 | 2 |
| 851 | MGAS27318 | United States | NY | 2013 | Invasive | 3 | 3 | -- | 101 | 1 |
| 852 | MGAS27320 | United States | TN | 2013 | Invasive | 3 | 3 | -- | 101 | 1 |
| 853 | MGAS27321 | United States | TN | 2013 | Invasive | 3 | 3 | -- | 101 | 1 |
| 854 | MGAS27322 | United States | TN | 2013 | Invasive | 3 | 3 | -- | 101 | 1 |
| 855 | MGAS27323 | United States | CA | 2013 | Invasive | 3 | 3 | -- | 101 | 1 |
| 856 | MGAS27324 | United States | NY | 2013 | Invasive | 3 | 3 | -- | 101 | 1 |
| 857 | MGAS27325 | United States | MD | 2013 | Invasive | 3 | 3 | -- | 101 | 1 |
| 858 | MGAS27326 | United States | MD | 2013 | Invasive | 3 | 3 | -- | 101 | 4 |
| 859 | MGAS27327 | United States | MN | 2013 | Invasive | 3 | 3 | -- | 101 | 7 |
| 860 | MGAS27328 | United States | TN | 2013 | Invasive | 3 | 3 | -- | 101 | 7 |
| 861 | MGAS27329 | United States | CO | 2013 | Invasive | 3 | 3 | -- | 101 | 1 |
| 862 | MGAS27330 | United States | NY | 2013 | Invasive | 3 | 3 | -- | 101 | 1 |
| 863 | MGAS27331 | United States | NM | 2013 | Invasive | 3 | 3 | -- | 101 | 1 |
| 864 | MGAS27332 | United States | NM | 2013 | Invasive | 3 | 3 | -- | 101 | 2 |
| 865 | MGAS27333 | United States | CA | 2013 | Invasive | 3 | 3 | -- | 101 | 2 |
| 866 | MGAS27334 | United States | CA | 2013 | Invasive | 3 | 3 | -- | 101 | 1 |
| 867 | MGAS27388 | United States | TN | 2009 | Invasive | 3 | 3 | -- | 101 | 1 |
| 868 | MGAS27389 | United States | TN | 2010 | Invasive | 3 | 3 | -- | NF | 1 |
| 869 | MGAS27390 | United States | TN | 2010 | Invasive | 3 | 3 | -- | 101 | 1 |
| 870 | MGAS27391 | United States | TN | 2010 | Invasive | 3 | 3 | -- | 101 | 1 |
| 871 | MGAS27392 | United States | OR | 2004 | Invasive | 3 | 3 | -- | 101 | 4 |
| 872 | MGAS27399 | United States | CA | 2004 | Invasive | 3 | 3 | -- | 101 | 1 |
| 873 | MGAS27400 | United States | CA | 2004 | Invasive | 3 | 3 | -- | 101 | 14 |
| 874 | MGAS27410 | United States | TN | 2010 | Invasive | 3 | 3 | -- | 101 | 1 |
| 875 | MGAS27411 | United States | TN | 2010 | Invasive | 3 | 3 | -- | 101 | 1 |
| 876 | MGAS27412 | United States | CA | 2011 | Invasive | 3 | 3 | -- | 101 | 1 |
| 877 | MGAS27413 | United States | GA | 2011 | Invasive | 3 | 3 | -- | 101 | 1 |
| 878 | MGAS27415 | United States | MN | 2010 | Invasive | 3 | 3 | -- | 101 | 1 |
| 879 | MGAS27418 | United States | MD | 2010 | Invasive | 3 | 3 | -- | 101 | 1 |
| 880 | MGAS27419 | United States | MD | 2010 | Invasive | 3 | 3 | -- | 101 | 4 |
| 881 | MGAS27420 | United States | MD | 2010 | Invasive | 3 | 3 | -- | 101 | 1 |
| 882 | MGAS27421 | United States | NY | 2011 | Invasive | 3 | 3 | -- | 101 | 1 |
| 883 | MGAS27422 | United States | GA | 2011 | Invasive | 3 | 3 | -- | NF | 1 |
| 884 | MGAS27423 | United States | CA | 2011 | Invasive | 3 | 3 | -- | NF | 7 |
| 885 | MGAS27425 | United States | CA | 2011 | Invasive | 3 | 3 | -- | 101 | 8 |
| 886 | MGAS27426 | United States | OR | 2011 | Invasive | 3 | 3 | -- | 101 | 19 |
| 887 | MGAS27428 | United States | MD | 2011 | Invasive | 3 | 3 | -- | 101 | 1 |
| 888 | MGAS27429 | United States | MD | 2011 | Invasive | 3 | 3 | -- | 101 | 1 |
| 889 | MGAS27430 | United States | MD | 2011 | Invasive | 3 | 3 | -- | 101 | 4 |
| 890 | MGAS27431 | United States | GA | 2011 | Invasive | 3 | 3 | -- | 101 | 1 |
| 891 | MGAS27432 | United States | GA | 2011 | Invasive | 3 | 3 | -- | 101 | 2 |
| 892 | MGAS27433 | United States | TN | 2011 | Invasive | 3 | 3 | -- | NF | 1 |
| 893 | MGAS27434 | United States | GA | 2010 | Invasive | 3 | 3 | -- | 101 | 1 |
| 894 | MGAS27435 | United States | GA | 2010 | Invasive | 3 | 3 | -- | 101 | 1 |
| 895 | MGAS27443 | United States | CA | 2006 | Invasive | 3 | 3 | -- | 101 | 1 |
| 896 | MGAS27447 | United States | NM | 2006 | Invasive | 3 | 3 | -- | 101 | 2 |
| 897 | MGAS27449 | United States | CA | 2010 | Invasive | 3 | 3 | -- | 101 | 1 |
| 898 | MGAS27451 | United States | NY | 2010 | Invasive | 3 | 3 | -- | 101 | 1 |
| 899 | MGAS27452 | United States | GA | 2010 | Invasive | 3 | 3 | -- | 101 | 1 |
| 900 | MGAS27453 | United States | GA | 2010 | Invasive | 3 | 3 | -- | 101 | 7 |
| 901 | MGAS27457 | Finland | -- | 2003 | Invasive | 3 | 3 | -- | 101 | 1 |
| 902 | MGAS27459 | Finland | -- | 2004 | Invasive | 3 | 3 | -- | 101 | 1 |
| 903 | MGAS27460 | Finland | -- | 2004 | Invasive | 3 | 3 | -- | 101 | 1 |
| 904 | MGAS27462 | Finland | -- | 2004 | Invasive | 3 | 3 | -- | 101 | 1 |
| 905 | MGAS27463 | Finland | -- | 2004 | Invasive | 3 | 3 | -- | 101 | 1 |
| 906 | MGAS27465 | Finland | -- | 2004 | Invasive | 3 | 3 | -- | 101 | 1 |
| 907 | MGAS27466 | Finland | -- | 2004 | Invasive | 3 | 3 | -- | 101 | 1 |
| 908 | MGAS27467 | Finland | -- | 2004 | Invasive | 3 | 3 | -- | NF | 1 |
| 909 | MGAS27468 | Finland | -- | 2004 | Invasive | 3 | 3 | -- | 101 | 1 |
| 910 | MGAS27469 | Finland | -- | 2005 | Invasive | 3 | 3 | -- | 101 | 2 |
| 911 | MGAS27470 | Finland | -- | 2005 | Invasive | 3 | 3 | -- | 101 | 1 |
| 912 | MGAS27472 | Finland | -- | 2005 | Invasive | 3 | 3 | -- | 101 | 19 |
| 913 | MGAS27473 | Finland | -- | 2006 | Invasive | 3 | 3 | -- | 101 | 1 |
| 914 | MGAS27475 | Finland | -- | 2006 | Invasive | 3 | 3 | -- | 101 | 1 |
| 915 | MGAS27476 | Finland | -- | 2006 | Invasive | 3 | 3 | -- | 101 | 1 |
| 916 | MGAS27477 | Finland | -- | 2006 | Invasive | 3 | 3 | -- | 101 | 2 |
| 917 | MGAS27479 | Finland | -- | 2006 | Invasive | 3 | 3 | -- | 101 | 1 |
| 918 | MGAS27480 | Finland | -- | 2006 | Invasive | 3 | 3 | -- | 101 | 1 |
| 919 | MGAS27481 | Finland | -- | 2006 | Invasive | 3 | 3 | -- | 101 | 1 |
| 920 | MGAS27482 | Finland | -- | 2006 | Invasive | 3 | 3 | -- | 101 | 1 |
| 921 | MGAS27483 | Finland | -- | 2006 | Invasive | 3 | 3 | -- | 101 | 1 |
| 922 | MGAS27485 | Finland | -- | 2007 | Invasive | 3 | 3 | -- | 101 | 1 |
| 923 | MGAS27486 | Finland | -- | 2007 | Invasive | 3 | 3 | -- | 101 | 1 |
| 924 | MGAS27487 | Finland | -- | 2007 | Invasive | 3 | 3 | -- | 101 | 1 |
| 925 | MGAS27488 | Finland | -- | 2007 | Invasive | 3 | 3 | -- | 101 | 1 |
| 926 | MGAS27490 | Finland | -- | 2007 | Invasive | 3 | 3 | -- | 101 | 1 |
| 927 | MGAS27493 | Finland | -- | 2007 | Invasive | 3 | 3 | -- | 101 | 1 |
| 928 | MGAS27494 | Finland | -- | 2007 | Invasive | 3 | 3 | -- | 101 | 2 |
| 929 | MGAS27495 | Finland | -- | 2007 | Invasive | 3 | 3 | -- | 101 | 7 |
| 930 | MGAS27496 | Finland | -- | 2008 | Invasive | 3 | 3 | -- | 101 | 2 |
| 931 | MGAS27497 | Finland | -- | 2008 | Invasive | 3 | 3 | -- | 101 | 1 |
| 932 | MGAS27498 | Finland | -- | 2008 | Invasive | 3 | 3 | -- | 101 | 1 |
| 933 | MGAS27499 | Finland | -- | 2008 | Invasive | 3 | 3 | -- | 101 | 1 |
| 934 | MGAS27500 | Finland | -- | 2008 | Invasive | 3 | 3 | -- | 101 | 1 |
| 935 | MGAS27501 | Finland | -- | 2008 | Invasive | 3 | 3 | -- | 101 | 1 |
| 936 | MGAS27502 | Finland | -- | 2008 | Invasive | 3 | 3 | -- | 101 | 1 |
| 937 | MGAS27503 | Finland | -- | 2008 | Invasive | 3 | 3 | -- | 101 | 1 |
| 938 | MGAS27504 | Finland | -- | 2008 | Invasive | 3 | 3 | -- | 101 | 2 |
| 939 | MGAS27505 | Finland | -- | 2008 | Invasive | 3 | 3 | -- | 101 | 1 |
| 940 | MGAS27507 | Finland | -- | 2009 | Invasive | 3 | 3 | -- | 101 | 1 |
| 941 | MGAS27509 | Finland | -- | 2009 | Invasive | 3 | 3 | -- | 101 | 1 |
| 942 | MGAS27510 | Finland | -- | 2009 | Invasive | 3 | 3 | -- | 101 | 1 |
| 943 | MGAS27511 | Finland | -- | 2009 | Invasive | 3 | 3 | -- | 101 | 1 |
| 944 | MGAS27512 | Finland | -- | 2009 | Invasive | 3 | 3 | -- | 101 | 1 |
| 945 | MGAS27513 | Finland | -- | 2009 | Invasive | 3 | 3 | -- | 101 | 1 |
| 946 | MGAS27515 | Finland | -- | 2009 | Invasive | 3 | 3 | -- | 101 | 30 |
| 947 | MGAS27517 | Finland | -- | 2009 | Invasive | 3 | 3 | -- | 101 | 1 |
| 948 | MGAS27518 | Finland | -- | 2009 | Invasive | 3 | 3 | -- | 101 | 4 |
| 949 | MGAS27521 | Finland | -- | 2009 | Invasive | 3 | 3 | -- | 101 | 1 |
| 950 | MGAS27522 | Finland | -- | 2009 | Invasive | 3 | 3 | -- | NF | 2 |
| 951 | MGAS27523 | Finland | -- | 2009 | Invasive | 3 | 3 | -- | 101 | 57 |
| 952 | MGAS27525 | Finland | -- | 2009 | Invasive | 3 | 3 | -- | 101 | 2 |
| 953 | MGAS27526 | Finland | -- | 2009 | Invasive | 3 | 3 | -- | 101 | 7 |
| 954 | MGAS27527 | Finland | -- | 2009 | Invasive | 3 | 3 | -- | 101 | 2 |
| 955 | MGAS27528 | Finland | -- | 2009 | Invasive | 3 | 3 | -- | 101 | 2 |
| 956 | MGAS27529 | Finland | -- | 2009 | Invasive | 3 | 3 | -- | 101 | 1 |
| 957 | MGAS27530 | Finland | -- | 2009 | Invasive | 3 | 3 | -- | 101 | 1 |
| 958 | MGAS27531 | Finland | -- | 2009 | Invasive | 3 | 3 | -- | 101 | 7 |
| 959 | MGAS27532 | Finland | -- | 2009 | Invasive | 3 | 3 | -- | 101 | 1 |
| 960 | MGAS27533 | Finland | -- | 2009 | Invasive | 3 | 3 | -- | 101 | 1 |
| 961 | MGAS27534 | Finland | -- | 2009 | Invasive | 3 | 3 | -- | 101 | 1 |
| 962 | MGAS27535 | Finland | -- | 2009 | Invasive | 3 | 3 | -- | 101 | 1 |
| 963 | MGAS27536 | Finland | -- | 2010 | Invasive | 3 | 3 | -- | 101 | 1 |
| 964 | MGAS27538 | Finland | -- | 2010 | Invasive | 3 | 3 | -- | 101 | 4 |
| 965 | MGAS27539 | Finland | -- | 2010 | Invasive | 3 | 3 | -- | 101 | 2 |
| 966 | MGAS27540 | Finland | -- | 2010 | Invasive | 3 | 3 | -- | 101 | 1 |
| 967 | MGAS27541 | Finland | -- | 2010 | Invasive | 3 | 3 | -- | 101 | 1 |
| 968 | MGAS27542 | Finland | -- | 2010 | Invasive | 3 | 3 | -- | 101 | 1 |
| 969 | MGAS27544 | Finland | -- | 2010 | Invasive | 3 | 3 | -- | 101 | 1 |
| 970 | MGAS27546 | Finland | -- | 2010 | Invasive | 3 | 3 | -- | 101 | 2 |
| 971 | MGAS27547 | Finland | -- | 2010 | Invasive | 3 | 3 | -- | 101 | 2 |
| 972 | MGAS27548 | Finland | -- | 2010 | Invasive | 3 | 3 | -- | 101 | 2 |
| 973 | MGAS27549 | Finland | -- | 2010 | Invasive | 3 | 3 | -- | 101 | 2 |
| 974 | MGAS27550 | Finland | -- | 2010 | Invasive | 3 | 3 | -- | 101 | 2 |
| 975 | MGAS27551 | Finland | -- | 2010 | Invasive | 3 | 3 | -- | 101 | 1 |
| 976 | MGAS27553 | Finland | -- | 2010 | Invasive | 3 | 3 | -- | 101 | 1 |
| 977 | MGAS27554 | Finland | -- | 2010 | Invasive | 3 | 3 | -- | 101 | 1 |
| 978 | MGAS27555 | Finland | -- | 2010 | Invasive | 3 | 3 | -- | 101 | 1 |
| 979 | MGAS27557 | Finland | -- | 2010 | Invasive | 3 | 3 | -- | 101 | 2 |
| 980 | MGAS27558 | Finland | -- | 2010 | Invasive | 3 | 3 | -- | 101 | 1 |
| 981 | MGAS27559 | Finland | -- | 2010 | Invasive | 3 | 3 | -- | 101 | 2 |
| 982 | MGAS27560 | Finland | -- | 2010 | Invasive | 3 | 3 | -- | NF | 2 |
| 983 | MGAS27562 | Finland | -- | 2010 | Invasive | 3 | 3 | -- | 101 | 1 |
| 984 | MGAS27563 | Finland | -- | 2011 | Invasive | 3 | 3 | -- | 101 | 1 |
| 985 | MGAS27564 | Finland | -- | 2011 | Invasive | 3 | 3 | -- | 101 | 1 |
| 986 | MGAS27565 | Finland | -- | 2011 | Invasive | 3 | 3 | -- | 101 | 1 |
| 987 | MGAS27566 | Finland | -- | 2011 | Invasive | 3 | 3 | -- | 101 | 1 |
| 988 | MGAS27567 | Finland | -- | 2011 | Invasive | 3 | 3 | -- | 101 | 1 |
| 989 | MGAS27568 | Finland | -- | 2011 | Invasive | 3 | 3 | -- | 101 | 1 |
| 990 | MGAS27569 | Finland | -- | 2011 | Invasive | 3 | 3 | -- | 101 | 1 |
| 991 | MGAS27570 | Finland | -- | 2011 | Invasive | 3 | 3 | -- | 101 | 1 |
| 992 | MGAS27571 | Finland | -- | 2011 | Invasive | 3 | 3 | -- | 101 | 1 |
| 993 | MGAS27572 | Finland | -- | 2011 | Invasive | 3 | 3 | -- | 101 | 2 |
| 994 | MGAS27573 | Finland | -- | 2011 | Invasive | 3 | 3 | -- | 101 | 1 |
| 995 | MGAS27575 | Finland | -- | 2011 | Invasive | 3 | 3 | -- | 101 | 1 |
| 996 | MGAS27576 | Finland | -- | 2011 | Invasive | 3 | 3 | -- | 101 | 1 |
| 997 | MGAS27578 | Finland | -- | 2011 | Invasive | 3 | 3 | -- | 101 | 1 |
| 998 | MGAS27579 | Finland | -- | 2011 | Invasive | 3 | 3 | -- | 101 | 2 |
| 999 | MGAS27581 | Finland | -- | 2011 | Invasive | 3 | 3 | -- | 101 | 7 |
| 1000 | MGAS27582 | Finland | -- | 2011 | Invasive | 3 | 3 | -- | 101 | 2 |
| 1001 | MGAS27583 | Finland | -- | 2011 | Invasive | 3 | 3 | -- | 101 | 1 |
| 1002 | MGAS27584 | Finland | -- | 2011 | Invasive | 3 | 3 | -- | 101 | 1 |
| 1003 | MGAS27585 | Finland | -- | 2011 | Invasive | 3 | 3 | -- | 101 | 2 |
| 1004 | MGAS27586 | Finland | -- | 2011 | Invasive | 3 | 3 | -- | 101 | 1 |
| 1005 | MGAS27587 | Finland | -- | 2011 | Invasive | 3 | 3 | -- | 101 | 2 |
| 1006 | MGAS27588 | Finland | -- | 2011 | Invasive | 3 | 3 | -- | 101 | 1 |
| 1007 | MGAS27589 | Finland | -- | 2011 | Invasive | 3 | 3 | -- | 101 | 1 |
| 1008 | MGAS27590 | Finland | -- | 2011 | Invasive | 3 | 3 | -- | 101 | 2 |
| 1009 | MGAS27591 | Finland | -- | 2011 | Invasive | 3 | 3 | -- | 101 | 1 |
| 1010 | MGAS27592 | Finland | -- | 2011 | Invasive | 3 | 3 | -- | 101 | 7 |
| 1011 | MGAS27593 | Finland | -- | 2012 | Invasive | 3 | 3 | -- | 101 | 1 |
| 1012 | MGAS27594 | Finland | -- | 2012 | Invasive | 3 | 3 | -- | 101 | 1 |
| 1013 | MGAS27595 | Finland | -- | 2012 | Invasive | 3 | 3 | -- | 101 | 1 |
| 1014 | MGAS27596 | Finland | -- | 2012 | Invasive | 3 | 3 | -- | 101 | 7 |
| 1015 | MGAS27597 | Finland | -- | 2012 | Invasive | 3 | 3 | -- | 101 | 1 |
| 1016 | MGAS27598 | Finland | -- | 2012 | Invasive | 3 | 3 | -- | 101 | 7 |
| 1017 | MGAS27600 | Finland | -- | 2012 | Invasive | 3 | 3 | -- | 101 | 8 |
| 1018 | MGAS27601 | Finland | -- | 2012 | Invasive | 3 | 3 | -- | 101 | 2 |
| 1019 | MGAS27602 | Finland | -- | 2012 | Invasive | 3 | 3 | -- | 101 | 1 |
| 1020 | MGAS27603 | Finland | -- | 2012 | Invasive | 3 | 3 | -- | 101 | 2 |
| 1021 | MGAS27604 | Finland | -- | 2012 | Invasive | 3 | 3 | -- | NF | 2 |
| 1022 | MGAS27605 | Finland | -- | 2012 | Invasive | 3 | 3 | -- | NF | 2 |
| 1023 | MGAS27606 | Finland | -- | 2012 | Invasive | 3 | 3 | -- | 101 | 2 |
| 1024 | MGAS27607 | Finland | -- | 2012 | Invasive | 3 | 3 | -- | 101 | 1 |
| 1025 | MGAS27608 | Finland | -- | 2012 | Invasive | 3 | 3 | -- | NF | 1 |
| 1026 | MGAS27610 | Finland | -- | 2012 | Invasive | 3 | 3 | -- | 101 | 1 |
| 1027 | MGAS27612 | Finland | -- | 2012 | Invasive | 3 | 3 | -- | 101 | 2 |
| 1028 | MGAS27613 | Finland | -- | 2012 | Invasive | 3 | 3 | -- | 101 | 1 |
| 1029 | MGAS27614 | Finland | -- | 2012 | Invasive | 3 | 3 | -- | 101 | 2 |
| 1030 | MGAS27615 | Finland | -- | 2012 | Invasive | 3 | 3 | -- | 101 | 2 |
| 1031 | MGAS27616 | Finland | -- | 2012 | Invasive | 3 | 3 | -- | 101 | 2 |
| 1032 | MGAS27617 | Finland | -- | 2012 | Invasive | 3 | 3 | -- | 101 | 1 |
| 1033 | MGAS27618 | Finland | -- | 2012 | Invasive | 3 | 3 | -- | NF | 2 |
| 1034 | MGAS27619 | Finland | -- | 2012 | Invasive | 3 | 3 | -- | 101 | 2 |
| 1035 | MGAS27620 | Finland | -- | 2012 | Invasive | 3 | 3 | -- | 101 | 1 |
| 1036 | MGAS27621 | Finland | -- | 2012 | Invasive | 3 | 3 | -- | 101 | 1 |
| 1037 | MGAS27622 | Finland | -- | 2012 | Invasive | 3 | 3 | -- | 101 | 2 |
| 1038 | MGAS27623 | Finland | -- | 2012 | Invasive | 3 | 3 | -- | 101 | 1 |
| 1039 | MGAS27624 | Finland | -- | 2012 | Invasive | 3 | 3 | -- | 101 | 2 |
| 1040 | MGAS27625 | Finland | -- | 2012 | Invasive | 3 | 3 | -- | 101 | 2 |
| 1041 | MGAS27626 | Finland | -- | 2012 | Invasive | 3 | 3 | -- | 101 | 1 |
| 1042 | MGAS27627 | Finland | -- | 2012 | Invasive | 3 | 3 | -- | 101 | 1 |
| 1043 | MGAS27628 | Finland | -- | 2012 | Invasive | 3 | 3 | -- | 101 | 1 |
| 1044 | MGAS27629 | Finland | -- | 2012 | Invasive | 3 | 3 | -- | 101 | 2 |
| 1045 | MGAS27630 | Finland | -- | 2012 | Invasive | 3 | 3 | -- | 101 | 1 |
| 1046 | MGAS27632 | Finland | -- | 2012 | Invasive | 3 | 3 | -- | 101 | 1 |
| 1047 | MGAS27634 | Finland | -- | 2012 | Invasive | 3 | 3 | -- | 101 | 1 |
| 1048 | MGAS27636 | Finland | -- | 2012 | Invasive | 3 | 3 | -- | 101 | 2 |
| 1049 | MGAS27637 | Finland | -- | 2012 | Invasive | 3 | 3 | -- | 101 | 1 |
| 1050 | MGAS27638 | Finland | -- | 2012 | Invasive | 3 | 3 | -- | 101 | 1 |
| 1051 | MGAS27639 | Finland | -- | 2012 | Invasive | 3 | 3 | -- | 101 | 1 |
| 1052 | MGAS27640 | Finland | -- | 2012 | Invasive | 3 | 3 | -- | 101 | 1 |
| 1053 | MGAS27641 | Finland | -- | 2012 | Invasive | 3 | 3 | -- | 101 | 2 |
| 1054 | MGAS27642 | Finland | -- | 2012 | Invasive | 3 | 3 | -- | 101 | 2 |
| 1055 | MGAS27643 | Finland | -- | 2012 | Invasive | 3 | 3 | -- | 101 | 1 |
| 1056 | MGAS27644 | Finland | -- | 2012 | Invasive | 3 | 3 | -- | 101 | 4 |
| 1057 | MGAS27646 | Finland | -- | 2012 | Invasive | 3 | 3 | -- | 101 | 2 |
| 1058 | MGAS27647 | Finland | -- | 2012 | Invasive | 3 | 3 | -- | NF | 1 |
| 1059 | MGAS27648 | Finland | -- | 2012 | Invasive | 3 | 3 | -- | 101 | 2 |
| 1060 | MGAS27649 | Finland | -- | 2012 | Invasive | 3 | 3 | -- | 101 | 1 |
| 1061 | MGAS27650 | Finland | -- | 2013 | Invasive | 3 | 3 | -- | 101 | 1 |
| 1062 | MGAS27651 | Finland | -- | 2013 | Invasive | 3 | 3 | -- | 101 | 1 |
| 1063 | MGAS27652 | Finland | -- | 2013 | Invasive | 3 | 3 | -- | 101 | 1 |
| 1064 | MGAS27653 | Finland | -- | 2013 | Invasive | 3 | 3 | -- | 101 | 2 |
| 1065 | MGAS27654 | Finland | -- | 2013 | Invasive | 3 | 3 | -- | 101 | 1 |
| 1066 | MGAS27655 | Finland | -- | 2013 | Invasive | 3 | 3 | -- | 101 | 2 |
| 1067 | MGAS27656 | Finland | -- | 2013 | Invasive | 3 | 3 | -- | 101 | 1 |
| 1068 | MGAS27657 | Finland | -- | 2013 | Invasive | 3 | 3 | -- | NF | 1 |
| 1069 | MGAS27658 | Finland | -- | 2013 | Invasive | 3 | 3 | -- | 101 | 1 |
| 1070 | MGAS27659 | Finland | -- | 2013 | Invasive | 3 | 3 | -- | 101 | 1 |
| 1071 | MGAS27661 | Finland | -- | 2013 | Invasive | 3 | 3 | -- | 101 | 1 |
| 1072 | MGAS27662 | Finland | -- | 2013 | Invasive | 3 | 3 | -- | 101 | 1 |
| 1073 | MGAS27663 | Finland | -- | 2013 | Invasive | 3 | 3 | -- | 101 | 1 |
| 1074 | MGAS27664 | Finland | -- | 2013 | Invasive | 3 | 3 | -- | 101 | 1 |
| 1075 | MGAS27665 | Finland | -- | 2013 | Invasive | 3 | 3 | -- | 101 | 7 |
| 1076 | MGAS27666 | Finland | -- | 2013 | Invasive | 3 | 3 | -- | 101 | 2 |
| 1077 | MGAS27667 | Finland | -- | 2013 | Invasive | 3 | 3 | -- | 101 | 1 |
| 1078 | MGAS27668 | Finland | -- | 2013 | Invasive | 3 | 3 | -- | 101 | 1 |
| 1079 | MGAS27669 | Finland | -- | 2013 | Invasive | 3 | 3 | -- | 101 | 1 |
| 1080 | MGAS27670 | Finland | -- | 2013 | Invasive | 3 | 3 | -- | 101 | 2 |
| 1081 | MGAS27671 | Finland | -- | 2013 | Invasive | 3 | 3 | -- | 101 | 1 |
| 1082 | MGAS27672 | Finland | -- | 2013 | Invasive | 3 | 3 | -- | 101 | 1 |
| 1083 | MGAS27673 | Finland | -- | 2013 | Invasive | 3 | 3 | -- | 101 | 1 |
| 1084 | MGAS27674 | Finland | -- | 2013 | Invasive | 3 | 3 | -- | 101 | 1 |
| 1085 | MGAS27676 | Finland | -- | 2013 | Invasive | 3 | 3 | -- | 101 | 1 |
| 1086 | MGAS27677 | Finland | -- | 2013 | Invasive | 3 | 3 | -- | 101 | 1 |
| 1087 | MGAS27678 | Finland | -- | 2013 | Invasive | 3 | 3 | -- | 101 | 1 |
| 1088 | MGAS27679 | Finland | -- | 2013 | Invasive | 3 | 3 | -- | 101 | 1 |
| 1089 | MGAS27680 | Finland | -- | 2013 | Invasive | 3 | 3 | -- | 101 | 2 |
| 1090 | MGAS27681 | Finland | -- | 2013 | Invasive | 3 | 3 | -- | 101 | 1 |
| 1091 | MGAS27682 | Finland | -- | 2013 | Invasive | 3 | 3 | -- | 101 | 1 |
| 1092 | MGAS27684 | Finland | -- | 2013 | Invasive | 3 | 3 | -- | 101 | 1 |
| 1093 | MGAS27685 | Finland | -- | 2013 | Invasive | 3 | 3 | -- | 101 | 1 |
| 1094 | MGAS27686 | Finland | -- | 2013 | Invasive | 3 | 3 | -- | 101 | 1 |
| 1095 | MGAS27687 | Finland | -- | 2013 | Invasive | 3 | 3 | -- | 101 | 2 |
| 1096 | MGAS27688 | Finland | -- | 2013 | Invasive | 3 | 3 | -- | 101 | 2 |
| 1097 | MGAS27690 | Finland | -- | 2013 | Invasive | 3 | 3 | -- | 101 | 1 |
| 1098 | MGAS27691 | Finland | -- | 2013 | Invasive | 3 | 3 | -- | NF | 1 |
| 1099 | MGAS27693 | Finland | -- | 2014 | Invasive | 3 | 3 | -- | 101 | 2 |
| 1100 | MGAS27694 | Finland | -- | 2014 | Invasive | 3 | 3 | -- | NF | 1 |
| 1101 | MGAS27695 | Finland | -- | 2014 | Invasive | 3 | 3 | -- | 101 | 1 |
| 1102 | MGAS27696 | Finland | -- | 2014 | Invasive | 3 | 3 | -- | 101 | 2 |
| 1103 | MGAS27698 | Finland | -- | 2014 | Invasive | 3 | 3 | -- | 101 | 1 |
| 1104 | MGAS27699 | Finland | -- | 2014 | Invasive | 3 | 3 | -- | 101 | 1 |
| 1105 | MGAS27701 | Finland | -- | 2014 | Invasive | 3 | 3 | -- | 101 | 1 |
| 1106 | MGAS27702 | Finland | -- | 2014 | Invasive | 3 | 3 | -- | 101 | 2 |
| 1107 | MGAS27704 | Finland | -- | 2014 | Invasive | 3 | 3 | -- | 101 | 1 |
| 1108 | MGAS27705 | Finland | -- | 2014 | Invasive | 3 | 3 | -- | NF | 1 |
| 1109 | MGAS27706 | Finland | -- | 2014 | Invasive | 3 | 3 | -- | 101 | 2 |
| 1110 | MGAS27707 | Finland | -- | 2014 | Invasive | 3 | 3 | -- | 101 | 2 |
| 1111 | MGAS27708 | Finland | -- | 2014 | Invasive | 3 | 3 | -- | 101 | 1 |
| 1112 | MGAS27709 | Finland | -- | 2014 | Invasive | 3 | 3 | -- | NF | 1 |
| 1113 | MGAS27711 | Finland | -- | 2014 | Invasive | 3 | 3 | -- | 101 | 1 |
| 1114 | MGAS27842 | Iceland | -- | 2007 | Invasive | 3 | 3 | -- | 101 | 1 |
| 1115 | MGAS27847 | Iceland | -- | 2008 | Invasive | 3 | 3 | -- | 101 | 1 |
| 1116 | MGAS27849 | Iceland | -- | 2008 | Invasive | 3 | 3 | -- | 101 | 1 |
| 1117 | MGAS27856 | Iceland | -- | 2009 | Invasive | 3 | 3 | -- | 101 | 1 |
| 1118 | MGAS27863 | Iceland | -- | 2010 | Invasive | 3 | 3 | -- | 101 | 1 |
| 1119 | MGAS27871 | Iceland | -- | 2011 | Invasive | 3 | 3 | -- | NF | 1 |
| 1120 | MGAS27878 | Iceland | -- | 2011 | Invasive | 3 | 3 | -- | 101 | 2 |
| 1121 | MGAS27881 | Iceland | -- | 2011 | Invasive | 3 | 3 | -- | 101 | 8 |
| 1122 | MGAS27899 | Iceland | -- | 2013 | Invasive | 3 | 3 | -- | 101 | 2 |
| 1123 | MGAS27910 | Iceland | -- | 2013 | Invasive | 3 | 3 | -- | 101 | 7 |
| 1124 | MGAS28981 | Finland | -- | 2014 | Invasive | 3 | 3 | -- | 101 | 1 |
| 1125 | MGAS28982 | Finland | -- | 2014 | Invasive | 3 | 3 | -- | 101 | 1 |
| 1126 | MGAS28983 | Finland | -- | 2014 | Invasive | 3 | 3 | -- | 101 | 1 |
| 1127 | MGAS28984 | Finland | -- | 2014 | Invasive | 3 | 3 | -- | 101 | 1 |
| 1128 | MGAS28986 | Finland | -- | 2014 | Invasive | 3 | 3 | -- | 101 | 2 |
| 1129 | MGAS28988 | Finland | -- | 2014 | Invasive | 3 | 3 | -- | 101 | 1 |
| 1130 | MGAS28990 | Finland | -- | 2014 | Invasive | 3 | 3 | -- | 101 | 2 |
| 1131 | MGAS28991 | Finland | -- | 2014 | Invasive | 3 | 3 | -- | 101 | 1 |
| 1132 | MGAS28992 | Finland | -- | 2014 | Invasive | 3 | 3 | -- | 101 | 2 |
| 1133 | MGAS28994 | Finland | -- | 2014 | Invasive | 3 | 3 | -- | 101 | 1 |
| 1134 | MGAS28997 | Finland | -- | 2014 | Invasive | 3 | 3 | -- | 101 | 1 |
| 1135 | MGAS28998 | Finland | -- | 2014 | Invasive | 3 | 3 | -- | 101 | 1 |
| 1136 | MGAS28999 | Finland | -- | 2014 | Invasive | 3 | 3 | -- | 101 | 2 |
| 1137 | MGAS29003 | Finland | -- | 2014 | Invasive | 3 | 3 | -- | 101 | 14 |
| 1138 | MGAS29004 | Finland | -- | 2014 | Invasive | 3 | 3 | -- | 101 | 4 |
| 1139 | MGAS29005 | Finland | -- | 2014 | Invasive | 3 | 3 | -- | 101 | 1 |
| 1140 | NGAS025 | Canada | ON | 2011 | Invasive | 3 | 3 | -- | 101 | 1 |
| 1141 | NGAS026 | Canada | ON | 2011 | Invasive | 3 | 3 | -- | 101 | 1 |
| 1142 | NGAS070 | Canada | ON | 2011 | Invasive | 3 | 3 | -- | 101 | 2 |
| 1143 | NGAS088 | Canada | ON | 2011 | Invasive | 3 | 3 | -- | 101 | 1 |
| 1144 | NGAS197 | Canada | ON | 2011 | Invasive | 3 | 3 | -- | 101 | 1 |
| 1145 | NGAS233 | Canada | ON | 2011 | Invasive | 3 | 3 | -- | 101 | 1 |
| 1146 | NGAS282 | Canada | ON | 2011 | Invasive | 3 | 3 | -- | 101 | 1 |
| 1147 | NGAS516 | Canada | ON | 2012 | Invasive | 3 | 3 | -- | 101 | 1 |
| 1148 | NGAS524 | Canada | ON | 2012 | Invasive | 3 | 3 | -- | 101 | 1 |
| 1149 | NGAS549 | Canada | ON | 2012 | Invasive | 3 | 3 | -- | 101 | 18 |
| 1150 | NGAS595 | Canada | ON | 2012 | Invasive | 3 | 3 | -- | 101 | 2 |
| 1151 | NGAS660 | Canada | ON | 2013 | Invasive | 3 | 3 | -- | 101 | 2 |
| 1152 | NGAS663 | Canada | ON | 2013 | Invasive | 3 | 3 | -- | 101 | 1 |
| 1153 | NGAS672 | Canada | ON | 2013 | Invasive | 3 | 3 | -- | 101 | 2 |
| 1154 | NGAS673 | Canada | ON | 2013 | Invasive | 3 | 3 | -- | 101 | 2 |
| 1155 | NGAS805 | Canada | ON | 2014 | Invasive | 3 | 3 | -- | 101 | 2 |
| 1156 | NGAS807 | Canada | ON | 2014 | Invasive | 3 | 3 | -- | 101 | 2 |
| 1157 | NGAS808 | Canada | ON | 2014 | Invasive | 3 | 3 | -- | 101 | 2 |
| 1158 | NGAS846 | Canada | ON | 2014 | Invasive | 3 | 3 | -- | 101 | 2 |
| 1159 | NGAS847 | Canada | ON | 2014 | Invasive | 3 | 3 | -- | 101 | 2 |
| 1160 | NGAS848 | Canada | ON | 2014 | Invasive | 3 | 3 | -- | 101 | 2 |
| 1161 | MGAS27508 | Finland | -- | 2009 | Invasive | 3D | 3 | -- | 101 | 2 |
| 1162 | MGAS27520 | Finland | -- | 2009 | Invasive | 3D | 3 | -- | 101 | 2 |
| 1163 | MGAS27543 | Finland | -- | 2010 | Invasive | 3D | 3 | -- | 101 | 2 |
| 1164 | MGAS27552 | Finland | -- | 2010 | Invasive | 3D | 3 | -- | 101 | 2 |
| 1165 | MGAS27556 | Finland | -- | 2010 | Invasive | 3D | 3 | -- | 101 | 2 |
| 1166 | MGAS27561 | Finland | -- | 2010 | Invasive | 3D | 3 | -- | 101 | 2 |
| 1167 | MGAS27574 | Finland | -- | 2011 | Invasive | 3D | 3 | -- | 101 | 2 |
| 1168 | MGAS27577 | Finland | -- | 2011 | Invasive | 3D | 3 | -- | NF | 2 |
| 1169 | MGAS27599 | Finland | -- | 2012 | Invasive | 3D | 3 | -- | 101 | 2 |
| 1170 | MGAS27631 | Finland | -- | 2012 | Invasive | 3D | 3 | -- | 101 | 2 |
| 1171 | MGAS27633 | Finland | -- | 2012 | Invasive | 3D | 3 | -- | 101 | 2 |
| 1172 | MGAS27635 | Finland | -- | 2012 | Invasive | 3D | 3 | -- | 101 | 2 |
| 1173 | MGAS27660 | Finland | -- | 2013 | Invasive | 3D | 3 | -- | 101 | 2 |
| 1174 | MGAS27675 | Finland | -- | 2013 | Invasive | 3D | 3 | -- | 101 | 2 |
| 1175 | MGAS27683 | Finland | -- | 2013 | Invasive | 3D | 3 | -- | NF | 2 |
| 1176 | MGAS27689 | Finland | -- | 2013 | Invasive | 3D | 3 | -- | 101 | 2 |
| 1177 | MGAS27692 | Finland | -- | 2013 | Invasive | 3D | 3 | -- | NF | 2 |
| 1178 | MGAS27697 | Finland | -- | 2014 | Invasive | 3D | 3 | -- | 101 | 2 |
| 1179 | MGAS27700 | Finland | -- | 2014 | Invasive | 3D | 3 | -- | 101 | 2 |
| 1180 | MGAS27703 | Finland | -- | 2014 | Invasive | 3D | 3 | -- | 101 | 2 |
| 1181 | MGAS27710 | Finland | -- | 2014 | Invasive | 3D | 3 | -- | 101 | 2 |
| 1182 | MGAS27712 | Finland | -- | 2014 | Invasive | 3D | 3 | -- | 101 | 2 |
| 1183 | MGAS27713 | Finland | -- | 2014 | Invasive | 3D | 3 | -- | 101 | 2 |
| 1184 | MGAS27714 | Finland | -- | 2014 | Invasive | 3D | 3 | -- | 101 | 2 |
| 1185 | MGAS28980 | Finland | -- | 2014 | Invasive | 3D | 3 | -- | 101 | 2 |
| 1186 | MGAS28985 | Finland | -- | 2014 | Invasive | 3D | 3 | -- | 101 | 2 |
| 1187 | MGAS28987 | Finland | -- | 2014 | Invasive | 3D | 3 | -- | 101 | 2 |
| 1188 | MGAS28989 | Finland | -- | 2014 | Invasive | 3D | 3 | -- | 101 | 2 |
| 1189 | MGAS28993 | Finland | -- | 2014 | Invasive | 3D | 3 | -- | 101 | 2 |
| 1190 | MGAS28995 | Finland | -- | 2014 | Invasive | 3D | 3 | -- | 101 | 2 |
| 1191 | MGAS28996 | Finland | -- | 2014 | Invasive | 3D | 3 | -- | 101 | 2 |
| 1192 | MGAS29000 | Finland | -- | 2014 | Invasive | 3D | 3 | -- | 101 | 2 |
| 1193 | MGAS29002 | Finland | -- | 2014 | Invasive | 3D | 3 | -- | 101 | 2 |
| 1194 | MGAS26564 | United States | CA | 1995 | Invasive | outlier | 2 | ? | NF | 35 |
| 1195 | MGAS26692 | United States | MD | 2001 | Invasive | outlier | 2 | ? | 142 | 23 |
| 1196 | MGAS26703 | United States | MD | 2002 | Invasive | outlier | 2 | ? | 142 | 23 |
| 1197 | MGAS27393 | United States | CA | 2004 | Invasive | outlier | 1 | ? | NF | 36 |
| 1198 | MGAS27450 | United States | CA | 2010 | Invasive | outlier | 1 | ? | 380 | 37 |
| 1199 | MGAS27537 | Finland | -- | 2010 | Invasive | outlier | 1 | ? | 380 | 52 |
| 1200 | MGAS27609 | Finland | -- | 2012 | Invasive | outlier | 1 | ? | 380 | 34 |

^a^ *nga-ifs-slo* promoter variants 1 and 2 are weakly expressing and variant 3 is strongly expressing.

^b^ NF is “not found”, either a specific allele or the 7-locus combination sequence type was not found in the *S. pyogenes* MLST database.
