# Supplementary material for: Transcriptome Remodeling Contributes to Epidemic Disease Caused by the Human Pathogen Streptococcus pyogenes
Source: mBio. 2016 May 31;7(3):e00403-16. doi: 10.1128/mBio.00403-16 (PMC4895104; doi:10.1128/mBio.00403-16)
Supplement: Table S2 — Streptococcus pyogenes complete genome sequences. [file mbo003162837st2.docx]

**Supplemental Table S2. *Streptococcus pyogenes* Complete Genome Sequences (as of 6/10/2015)**

| **No.** | **M-type** | **Strain** | **NCBI Accession** | **Length (nts)** | **%G+C** | **Genes** | **CDSs** | **Release Date** |
| --- | --- | --- | --- | --- | --- | --- | --- | --- |
| 1 | 1^a^ | SF370^a^ | AE004092 | 1,852,433 | 38.5 | 1810 | 1696 | 4/10/01 |
| 2 | 1^a^ | MGAS5005^a^ | CP000017 | 1,838,562 | 38.5 | 1950 | 1865 | 8/5/05 |
| 3 | 1^a^ | M1-476^a^ | AP012491 | 1,831,128 | 38.5 | 1644 | 1571 | 7/14/12 |
| 4 | 1^a^ | A20^a^ | CP003901 | 1,837,281 | 38.5 | 1915 | 1828 | 10/22/12 |
| 5 | 1 | AP1 | CP007537 | 1,908,294 | 38.5 | 1940 | 1836 | 5/7/15 |
| 6 | 1 | NCTC8198 | LN831034 | 1,914,862 | 38.5 | 1959 | 1891 | 5/2/15 |
| 7 | 1 | MTB313 | AP014572 | 1,745,332 | 38.5 | 1843 | 1758 | 6/4/15 |
| 8 | 1 | MTB314 | AP014585 | 1,744,827 | 38.5 | 1740 | 1658 | 6/4/15 |
| 9 | 1 | 5448 | CP008776 | 1,829,516 | 38.5 | 1814 | 1723 | 6/8/15 |
| 10 | 2^a^ | MGAS10270^a^ | CP000260 | 1,928,252 | 38.4 | 2068 | 1987 | 1/31/14 |
| 11 | 3^a^ | MGAS315^a^ | AE014074 | 1,900,521 | 38.6 | 1951 | 1865 | 7/18/02 |
| 12 | 3^a^ | SSI-1^a^ | BA000034 | 1,894,275 | 38.6 | 1931 | 1859 | 3/4/03 |
| 13 | 3^a^ | STAB902^a^ | CP007041 | 1,892,124 | 38.5 | 1918 | 1809 | 7/22/14 |
| 14 | 4^a^ | MGAS10750^a^ | CP000262 | 1,937,111 | 38.3 | 2059 | 1978 | 5/4/06 |
| 15 | 5^a^ | Manfredo^a^ | AM295007 | 1,841,271 | 38.6 | 1906 | 1745 | 2/5/07 |
| 16 | 6^a^ | MGAS10394^a^ | CP000003 | 1,899,877 | 38.7 | 1971 | 1886 | 8/3/04 |
| 17 | 6 | D471 | CP011415 | 1,811,968 | 38.6 | 1793 | 1671 | 6/2/15 |
| 18 | 6 | JRS4 | CP011414 | 1,811,968 | 38.6 | 1793 | 1671 | 6/2/15 |
| 19 | 12^a^ | MGAS9429^a^ | CP000259 | 1,836,467 | 38.5 | 1962 | 1877 | 5/4/06 |
| 20 | 12^a^ | MGAS2096^a^ | CP000261 | 1,860,355 | 38.7 | 1979 | 1898 | 5/4/06 |
| 21 | 12^a^ | HKU16^a^ | AFRY01000001 | 1,908,100 | 38.5 | 1871 | 1771 | 5/18/12 |
| 22 | 12^a^ | HKU360^a^ | CP009612 | 1,944,537 | 38.5 | 1946 | 1846 | 11/5/14 |
| 23 | 14^a^ | HSC5^a^ | CP006366 | 1,818,351 | 38.5 | 1856 | 1744 | 7/11/13 |
| 24 | 18^a^ | MGAS8232^a^ | AE009949 | 1,895,017 | 38.5 | 1924 | 1839 | 1/31/02 |
| 25 | 23^a^ | M23ND^a^ | CP008695 | 1,846,477 | 38.6 | 1925 | 1842 | 9/22/14 |
| 26 | 28^a^ | MGAS6180^a^ | CP000056 | 1,897,573 | 38.4 | 1977 | 1894 | 8/4/05 |
| 27 | 28 | M28PF1 | CP011535 | 1,896,976 | 38.4 | 1881 | 1765 | 6/5/15 |
| 28 | 44^a^ | STAB901^a^ | CP007024 | 1,795,609 | 38.5 | 1812 | 1358 | 7/22/14 |
| 29 | 44^a^ | 1E1^a^ | CP007241 | 1,796,152 | 38.5 | 1781 | 1652 | 11/5/14 |
| 30 | 49^a^ | NZ131^a^ | CP000829 | 1,815,785 | 38.6 | 1788 | 1700 | 10/16/08 |
| 31 | 53^a^ | Alab49^a^ | CP003068 | 1,827,308 | 38.6 | 1866 | 1773 | 10/7/11 |
| 32 | 59^a^ | MGAS15252^a^ | CP003116 | 1,750,832 | 38.5 | 1757 | 1662 | 3/1/12 |
| 33 | 59^a^ | MGAS1882^a^ | CP003121 | 1,781,029 | 38.5 | 1792 | 1691 | 3/1/12 |
| 34 | 82 | NGAS596 | CP007561 | 1,791,306 | 38.5 | 1745 | 1626 | 6/3/15 |
| 35 | 83^a^ | 7F7\|STAB1101^a^ | CP007240 | 1,709,790 | 38.6 | 1657 | 1550 | 10/20/14 |
| 36 | 83 | NGAS327 | CP007562 | 1,702,054 | 38.6 | 1648 | 1546 | 6/3/15 |
| 37 | 83 | STAB1102 | CP007023 | 1,709,442 | 38.6 | 1697 | 1582 | 10/1/14 |
| 38 | 87 | NGAS743 | CP007560 | 1,915,554 | 38.5 | 1927 | 1807 | 6/3/15 |
| 39 | 89^a^ | MGAS11027^a^ | CP013838 | 1,786,874 | 38.6 | 1774 | 1677 |  |
| 40 | 89^a^ | MGAS23530^a^ | CP013839 | 1,709,394 | 38.5 | 1685 | 1593 |  |
| 41 | 89^a^ | MGAS27061^a^ | CP013840 | 1,741,348 | 38.5 | 1717 | 1626 |  |
| 42 | ?^a^ | ATCC-19615^a^ | CP008926 | 1,844,804 | 38.5 | 1866 | 1775 | 8/22/14 |

^a^ Indicates the 18 serotypes types and 30 strains included in construction of the GAS-30 pseudo-pangenome.
