# Supplementary material for: Transcriptome Remodeling Contributes to Epidemic Disease Caused by the Human Pathogen Streptococcus pyogenes
Source: mBio. 2016 May 31;7(3):e00403-16. doi: 10.1128/mBio.00403-16 (PMC4895104; doi:10.1128/mBio.00403-16)
Supplement: Table S3 — RNAseq transcriptome analyses. [file mbo003162837st3.docx]

**Table S3. RNAseq Transcriptome Analyses**

**Section 1: Genes differentially expressed between clade 1 strain MGAS11027 and clade 2 strain MGAS23530**

|  |  |  |  | **C2/C1**  **Fold Change** | |  |
| --- | --- | --- | --- | --- | --- | --- |
| **No.** | **Locus Tag^a^** | **Gene** | **Product/Function** | **ME** | **ES** | **RB** |
| 1 | 38 |  | hypothetical protein | 1.50 | -1.54 |  |
| 2 | 39 |  | ribose-phosphate pyrophosphokinase | -1.55 |  |  |
| 3 | 41 | *plsX* | phosphate:acyl-ACP acyltransferase protein PlsX |  | 1.89 |  |
| 4 | 42 | *acp* | acyl carrier protein Acp |  | 2.39 |  |
| 5 | 43 | *purC* | phosphoribosylaminoimidazole-succinocarboxamide synthase protein PurC | -1.63 |  |  |
| 6 | 44 | *purL* | phosphoribosylformylglycinamidine synthase protein PurL | -1.85 |  |  |
| 7 | 45 |  | amidophosphoribosyltransferase protein PurF | -1.85 |  |  |
| 8 | 46 | *purM* | phosphoribosylformylglycinamidine cyclo-ligase protein PurM | -1.84 |  |  |
| 9 | 47 | *purN* | phosphoribosylglycinamide formyltransferase protein PurN | -1.73 |  |  |
| 10 | 48 | *purH* | IMP cyclohydrolase protein PurH | -2.24 |  |  |
| 11 | 49 |  | putative amidase/autolysin/surface antigen | -1.66 |  |  |
| 12 | 61 | *adh2* | alcohol dehydrogenase/acetaldehyde dehydrogenase protein Adh2 | 3.82 | -1.72 |  |
| 13 | 62 | *adh1* | alcohol dehydrogenase protein Adh1 | 3.02 | -3.20 |  |
| 14 | 64 | *rpsJ* | SSU ribosomal protein (S10p) RpsJ |  | 2.19 |  |
| 15 | 65 | *rplC* | LSU ribosomal protein (L3p) RplC |  | 2.78 |  |
| 16 | 66 | *rplD* | LSU ribosomal protein (L4p) RplD |  | 2.99 |  |
| 17 | 67 | *rplW* | LSU ribosomal protein (L23p) RplW |  | 3.26 |  |
| 18 | 68 | *rplB* | LSU ribosomal protein (L2p) RplB |  | 3.16 |  |
| 19 | 69 | *rpsS* | SSU ribosomal protein (S19p) RpsS |  | 3.49 |  |
| 20 | 70 | *RplV* | LSU ribosomal protein (L22p) RplV |  | 3.12 |  |
| 21 | 71 | *rpsC* | SSU ribosomal protein (S3p) RpsC |  | 3.02 |  |
| 22 | 72 | *rplP* | LSU ribosomal protein (L16p) RplP |  | 3.84 |  |
| 23 | 73 | *rpmC* | LSU ribosomal protein (L29p) RpmC |  | 4.01 |  |
| 24 | 74 | *rpsQ* | SSU ribosomal protein (S17p) RpsQ |  | 3.65 |  |
| 25 | 75 | *rplN* | LSU ribosomal protein (L14p) RplN |  | 3.01 |  |
| 26 | 76 | *rplX* | LSU ribosomal protein (L24p) RplX |  | 3.51 |  |
| 27 | 77 | *rplE* | LSU ribosomal protein (L5p) RplE |  | 2.27 |  |
| 28 | 78 | *rpsN.1* | SSU ribosomal protein (S14p) RpsN |  | 2.52 |  |
| 29 | 79 | *rpsH* | SSU ribosomal protein (S8p) RpsH |  | 3.10 |  |
| 30 | 80 | *rplF* | LSU ribosomal protein (L6p) RplF |  | 3.28 |  |
| 31 | 81 | *rplR* | LSU ribosomal protein (L18p) RplR |  | 2.39 |  |
| 32 | 82 | *rpsE* | SSU ribosomal protein (S5p) RpsE |  | 2.08 |  |
| 33 | 83 | *rpmD* | LSU ribosomal protein (L30p) RpmD |  | 2.39 |  |
| 34 | 84 | *rplO* | LSU ribosomal protein (L15p) RplO |  | 3.12 |  |
| 35 | 85 | *secY* | preprotein translocase subunit SecY |  | 3.32 |  |
| 36 | 86 | *adk* | adenylate kinase protein Adk |  | 1.78 |  |
| 37 | 87 | *infA* | translation initiation factor 1 protein InfA | 1.64 | 1.80 |  |
| 38 | 110 |  | hypothetical protein | 2.48 |  |  |
| 39 | 111 |  | kinase |  | -1.52 |  |
| 40 | 113 | *adcR* | transcriptional repressor protein AdcR |  | -1.68 |  |
| 41 | 114 | *adcC* | zinc ABC transporter ATP-binding protein AdcC |  | -1.77 |  |
| 42 | 122 | *comYA* | competence protein ComYA | 1.65 |  | RB01 |
| 43 | 130 | *ackA* | acetate kinase protein AckA |  | -2.23 | RB01 |
| 44 | 134 |  | hypothetical protein |  | -2.33 | RB01 |
| 45 | 137 |  | tRNA binding domain containing protein |  | 1.73 | RB01 |
| 46 | 139 |  | deoxyribonucleotide kinase | -2.16 | -2.01 | RB01 |
| 47 | 143 |  | hypothetical protein |  | -2.05 | RB01 |
| 48 | 144 | *prtF1* | fibronectin-binding protein I PrtF1/SfbI | 1.55 |  | RB01 |
| 49 | 145 | *srtC* | sortase protein SrtC |  | -1.65 | RB01 |
| 50 | 146 | *cpa* | fimbrial minor sturctural protein Cpa/FctA | 1.51 | -1.82 | RB01 |
| 51 | 147 |  | signal peptidase I protein SipA/LepA |  | -2.89 | RB01 |
| 52 | 148 | *tee* | T antigen/fimbrial major structural protein A | 1.91 |  | RB01 |
| 53 | 149 | *srtB* | sortase B protein SrtB |  | -1.68 | RB01 |
| 54 | 151 | *msmR* | FCT region positive transcriptional regulatory protein MsmR |  | 2.28 | RB01 |
| 55 | 152 | *prtF2* | fibronectin-binding protein 2 PrtF2/FbaB | 1.58 | 1.75 | RB01 |
| 56 | 153 |  | hypothetical protein | -1.75 | -1.53 | RB01 |
| 57 | 154 | *atoE* | short chain fatty acids transport protein AtoE | 2.03 |  | RB01 |
| 58 | 155 |  | LysR family transcriptional regulator | 2.00 |  | RB01 |
| 59 | 156 | *yiqL* | acetyl-CoA acetyltransferase YiqL | -2.67 |  | RB01 |
| 60 | 157 | *atoD.1* | butyrate-acetoacetate CoA-transferase alpha subunit AtoD | -3.03 |  | RB01 |
| 61 | 158 | *atoA.1* | butyrate-acetoacetate CoA-transferase beta subunit AtoA | -2.86 |  | RB01 |
| 62 | 160 |  | hypothetical protein |  | 1.80 | RB01 |
| 63 | 162 |  | hypothetical protein | 1.62 |  | RB01 |
| 64 | 163 | *ntpI* | V-type Na+-ATPase subunit (I) NtpI | 1.59 |  | RB01 |
| 65 | 167 | *ntpF* | V-type Na+-ATPase synthase subunit (F) NtpF | 1.62 |  | RB01 |
| 66 | 168 | *ntpA* | V-type Na+-ATPase synthase subunit (A) NtpA | 1.63 |  | RB01 |
| 67 | 170 | *ntpD* | V-type Na+-ATPase synthase subunit (D) NtpD | 1.53 |  | RB01 |
| 68 | 171 |  | putative toxic anion resistance protein | -1.56 |  | RB01 |
| 69 | 172 |  | hypothetical protein |  | 1.60 | RB01 |
| 70 | 173 | *purA* | adenylosuccinate synthetase protein PurA |  | 1.62 | RB01 |
| 71 | 176 | *nga* | nicotine adenine dinucleotide glycohydrolase protein Nga | 1.96 |  | RB01 |
| 72 | 177 | *ifs* | immunity factor protein Ifs | 1.72 | -1.56 | RB01 |
| 73 | 178 | *slo* | streptolysin O precursor protein Slo | 2.14 |  | RB01 |
| 74 | 182 |  | hypothetical secreted protein | 69.96 | 77.21 | RB01 |
| 75 | 183 | *metB* | cystathionine beta-lyase protein MetB |  | -1.56 | RB01 |
| 76 | 192 |  | transcriptional antiterminator | 1.78 |  |  |
| 77 | 202 |  | hypothetical protein | -1.59 |  |  |
| 78 | 203 |  | BioY family protein | -1.60 |  |  |
| 79 | 204 |  | putative metal-dependent hydrolase |  | 1.65 |  |
| 80 | 205 |  | putative tRNA-specific adenosine deaminase |  | 1.80 |  |
| 81 | 207 | *speG* | pyrogenic exotoxin protein (G) SpeG | 1.52 |  |  |
| 82 | 208 | *pgi* | glucose-6-phosphate isomerase protein Pgi |  | 1.77 |  |
| 83 | 209 |  | RofA-like transcriptional regulator |  | -2.87 |  |
| 84 | 230 | *rpmH* | LSU ribosomal protein (L34p) RpmH |  | 1.53 |  |
| 85 | 231 | *nanE* | N-acetylmannosamine-6-phosphate epimerase protein NanE |  | -12.45 |  |
| 86 | 232 |  | N-acetylneuraminate-binding lipoprotein |  | -13.19 |  |
| 87 | 233 |  | N-acetylneuraminate transport system permease protein 1 |  | -11.05 |  |
| 88 | 234 |  | N-acetylneuraminate transport system permease protein 2 |  | -9.11 |  |
| 89 | 235 |  | hypothetical protein |  | -15.60 |  |
| 90 | 236 |  | hypothetical protein |  | -7.28 |  |
| 91 | 237 | *nanA* | N-acetylneuraminate lyase protein NanA |  | -3.40 |  |
| 92 | 238 |  | N-acetylmannosamine kinase |  | -7.98 |  |
| 93 | 239 |  | sialic acid utilization regulator |  | -2.32 |  |
| 94 | 248 | *purR* | transcription regulatory protein PurR | 1.51 |  |  |
| 95 | 250 | *rpsL* | SSU ribosomal protein (S12p) RpsL |  | 1.74 |  |
| 96 | 256 |  | hypothetical protein |  | 1.99 |  |
| 97 | 257 |  | undecaprenyl-diphosphatase |  | 1.65 |  |
| 98 | 260 | *sufC* | iron-sulfur cluster assembly ATPase protein (C) SufC |  | -1.63 |  |
| 99 | 261 | *sufD* | iron-sulfur cluster assembly protein (D) SufD |  | -1.63 |  |
| 100 | 262 | *sufS* | cysteine desulfurase protein SufS |  | -1.50 |  |
| 101 | 266 | *dacA* | D-alanyl-D-alanine carboxypeptidase protein DacA |  | -1.63 |  |
| 102 | 267 | *oppA* | oligopeptide-binding lipoprotein OppA |  | -1.81 |  |
| 103 | 268 | *oppB* | oligopeptide transport system permease protein OppB |  | -1.59 |  |
| 104 | 269 | *oppC* | oligopeptide transport system permease protein OppC |  | -1.65 |  |
| 105 | 281 |  | HAD family phosphohydrolase |  | -1.82 |  |
| 106 | 291 | *atmA* | cysteine ABC transport system substrate-binding lipoprotein AtmA | 1.64 |  |  |
| 107 | 292 | *atmB* | methionine ABC transport system substrate-binding lipoprotein AtmB |  | 1.80 |  |
| 108 | 293 | *atmD* | methionine ABC transporter ATP-binding protein AtmD |  | 1.72 |  |
| 109 | 294 | *atmE* | methionine ABC transport system permease protein AtmE | 1.69 |  |  |
| 110 | 296 |  | dicarboxylate/amino acid:cation (Na+ or H+) symporter |  | 1.58 |  |
| 111 | 300 |  | LemA-like protein |  | 1.57 |  |
| 112 | 301 | *hptX* | heat shock protein HtpX |  | 1.59 |  |
| 113 | 305 | *nrdR* | transcriptional regulatory protein NrdR |  | -1.57 |  |
| 114 | 314 | *greA* | transcription elongation factor GreA |  | -1.82 |  |
| 115 | 316 |  | acylphosphatase |  | 1.94 | RB02 |
| 116 | 317 |  | TrmH family RNA methyltransferase |  | 1.52 |  |
| 117 | 318 |  | HD family hydrolase | 1.55 |  |  |
| 118 | 319 |  | YccA-like protein |  | 1.62 |  |
| 119 | 341 | *fhuD* | ferrichrome-binding lipoprotein precursor FhuD |  | 1.53 |  |
| 120 | 342 | *fhuC* | ferrichrome transport ATP-binding protein FhuC |  | 1.98 |  |
| 121 | 345 | *upp* | uracil phosphoribosyltransferase protein Upp | -1.62 |  |  |
| 122 | 346 | *clpP* | ATP-dependent Clp protease proteolytic subunit ClpP |  | 1.67 |  |
| 123 | 358 | *lctO* | lactate 2-monooxygenase protein LctO | 1.65 | 2.08 |  |
| 124 | 364 |  | hypothetical protein | -1.65 |  |  |
| 125 | 365 | *nrdF.1* | ribonucleotide reductase beta subunit NrdF | -1.60 |  |  |
| 126 | 366 | *nrdI.1* | ribonucleotide reductase stimulatory protein NrdI | -1.50 |  |  |
| 127 | 367 | *nrdE* | ribonucleotide reductase alpha subunit NrdE | -1.69 |  |  |
| 128 | 368 |  | VIP2-like ADP-ribosyltransferase toxin |  | -1.59 |  |
| 129 | 370 |  | hypothetical protein | -2.33 | -1.50 |  |
| 130 | 372 |  | transposase |  | 1.54 |  |
| 131 | 373 |  | transposase |  | 1.55 |  |
| 132 | 375 |  | hypothetical protein | -3.49 | -1.94 |  |
| 133 | 376 |  | hypothetical protein | -13.34 | -24.77 |  |
| 134 | 378 |  | hypothetical protein |  | 1.52 |  |
| 135 | 379 |  | hypothetical protein | 1.83 | -1.79 |  |
| 136 | 380 |  | hypothetical protein | 1.66 | -1.86 |  |
| 137 | 382 |  | hypothetical protein |  | 1.97 |  |
| 138 | 383 |  | integrase |  | 2.23 |  |
| 139 | 384 | *fabG.1* | 3-Ketoacyl-ACP reductase protein FabG |  | 1.74 |  |
| 140 | 385 |  | NAD-dependent oxidoreductase |  | 1.86 |  |
| 141 | 392 | *mtsR* | iron-dependent transcriptional regulatory protein MtsR |  | 1.59 |  |
| 142 | 398 |  | hypothetical protein |  | -2.18 |  |
| 143 | 400 | *rplK* | 50S ribosomal protein L11, LSU ribosomal protein L11P, RplK |  | 1.64 |  |
| 144 | 401 | *rplA* | 50S ribosomal protein L1, LSU ribosomal protein L1P, RplA |  | 1.87 |  |
| 145 | 403 | *frr* | ribosome recycling factor protein Frr |  | -1.69 |  |
| 146 | 407 |  | putative surface immunogenic protein | 1.69 |  |  |
| 147 | 408 |  | myosin-crossreactive streptococcal antigen |  | 1.85 |  |
| 148 | 416 |  | putative bacteriocin | 1.83 |  |  |
| 149 | 424 |  | hypothetical protein |  | 1.77 |  |
| 150 | 425 |  | hypothetical protein |  | 2.16 |  |
| 151 | 426 |  | Rgg-like transcriptional regulator | 1.87 | 2.60 |  |
| 152 | 427 | *fpg* | formamidopyrimidine-DNA glycosylase protein Fpg |  | 1.99 |  |
| 153 | 428 |  | dephospho-CoA kinase |  | 1.91 |  |
| 154 | 432 | *secG* | preprotein translocase subunit SecG |  | -2.14 |  |
| 155 | 433 | *vacB* | 3'-to-5' exoribonuclease protein VacB |  | -1.74 |  |
| 156 | 434 | *smpB* | tmRNA-binding protein SmpB |  | -1.81 |  |
| 157 | 435 |  | glutaminyl-peptide cyclotransferase |  | -1.78 |  |
| 158 | 440 | *gloA* | lactoylglutathione lyase protein GloA |  | 2.41 |  |
| 159 | 441 |  | oxygen-insensitive NAD(P)H nitroreductase/dihydropteridine reductase |  | 2.24 |  |
| 160 | 442 | *pepP.1* | Xaa-Pro dipeptidase protein PepP |  | 2.56 |  |
| 161 | 443 | *ccpA* | catabolite control protein CcpA |  | -2.75 |  |
| 162 | 446 |  | threonyl-tRNA synthetase protein ThrS |  | 2.58 |  |
| 163 | 447 |  | ABC transport system ATP-binding protein |  | -2.97 |  |
| 164 | 448 |  | ABC transport system permease |  | -1.86 |  |
| 165 | 449 |  | ABC transport system permease |  | -1.59 |  |
| 166 | 451 |  | 3-ketoacyl-CoA thiolase/acetyl-CoA acetyltransferase |  | 2.42 |  |
| 167 | 452 |  | acyl-CoA synthetases/AMP-acid ligases |  | 2.45 |  |
| 168 | 458 | *smc* | chromosome partition protein Smc |  | 1.61 |  |
| 169 | 459 |  | Rgg-like transcriptional regulator | -1.50 | -1.74 |  |
| 170 | 463 |  | hypothetical protein | 1.61 |  |  |
| 171 | 464 |  | S-adenosylmethionine synthetase | 1.51 | 1.75 |  |
| 172 | 465 |  | hypothetical protein | 1.53 | 1.84 |  |
| 173 | 472 |  | hypothetical protein |  | 3.71 |  |
| 174 | 473 |  | hypothetical protein |  | 2.89 |  |
| 175 | 474 | *relB* | plasmid stabilization system antitoxin protein RelB |  | 2.19 |  |
| 176 | 475 | *relE* | plasmid stabilization system toxin protein RelE |  | 2.48 |  |
| 177 | 476 |  | hypothetical protein |  | 2.32 |  |
| 178 | 483 | *licT* | beta-glucoside bgl operon antiterminator protein LicT | 2.66 |  |  |
| 179 | 484 |  | PTS system beta-glucoside-specific IIB-IIC-IIA components | 2.77 |  |  |
| 180 | 485 | *bglB.1* | 6-phospho-beta-glucosidase protein BglB | 2.06 |  |  |
| 181 | 486 |  | hypothetical protein | -1.55 | 1.66 |  |
| 182 | 490 |  | putative transcription elongation metalloprotease |  | 2.13 |  |
| 183 | 491 |  | putative stress-responsive transcriptional regulator | -1.53 |  |  |
| 184 | 496 |  | hypothetical protein |  | -1.59 |  |
| 185 | 507 |  | phage lysin glycosyl hydrolase |  | -1.53 |  |
| 186 | 509 |  | hypothetical protein |  | 1.53 |  |
| 187 | 510 |  | glutathione peroxidase |  | 1.77 |  |
| 188 | 511 | *pepF.1* | oligoendopeptidase protein PepF |  | 1.51 |  |
| 189 | 516 | *tpiA* | triosephosphate isomerase protein TpiA |  | 2.68 |  |
| 190 | 517 | *murN* | peptidoglycan lipid II-Ala--L-alanine ligase protein MurN |  | 1.64 |  |
| 191 | 518 | *murM* | peptidoglycan lipid II--L-alanine ligase protein MurM |  | 1.51 |  |
| 192 | 522 |  | hypothetical protein |  | 1.51 |  |
| 193 | 525 |  | oligohyaluronate lyase |  | -1.71 |  |
| 194 | 526 |  | PTS system hyaluronate-oligosaccharide-specific IID component | 2.29 |  |  |
| 195 | 527 |  | PTS system hyaluronate-oligosaccharide-specific IIC component | 2.32 |  |  |
| 196 | 528 |  | PTS system hyaluronate-oligosaccharide-specific IIB component | 2.14 |  |  |
| 197 | 529 |  | unsaturated glucuronyl hydrolase | 2.74 |  |  |
| 198 | 530 |  | PTS system hyaluronate-oligosaccharide-specific IIA component | 2.57 |  |  |
| 199 | 531 |  | 5-keto-D-gluconate 5-reductase | 2.09 | 1.90 |  |
| 200 | 532 |  | putative 4-deoxy-L-threo-5- hexosulose-uronate ketol-isomerase | 2.06 | 2.00 |  |
| 201 | 533 | *kdgK* | 2-dehydro-3-deoxygluconate kinase protein KdgK | 1.57 | 1.94 |  |
| 202 | 534 | *kdgA* | 4-Hydroxy-2-oxoglutarate aldolase protein KgdA |  | 2.02 |  |
| 203 | 542 | *dinG* | DinG family ATP-dependent helicase |  | -1.54 |  |
| 204 | 543 | *aspC* | aspartate aminotransferase protein AspC |  | -1.60 |  |
| 205 | 548 | *pepD.1* | dipeptidase protein PepD | -2.29 | -6.11 |  |
| 206 | 549 | *acdA* | metal-binding protein AcdA | -2.26 | -4.17 |  |
| 207 | 552 | *rpmE* | LSU ribosomal protein (L31p) RpmE |  | -2.33 |  |
| 208 | 553 |  | exopolyphosphatase-related protein |  | -1.70 |  |
| 209 | 555 |  | chorismate mutase |  | -1.83 |  |
| 210 | 556 |  | chloride channel protein | -1.72 | -1.68 |  |
| 211 | 557 | *rplS* | LSU ribosomal protein (L19p) RplS |  | 1.97 |  |
| 212 | 559 |  | HAD-like hydrolase |  | -1.87 |  |
| 213 | 560 | *gyrB* | DNA gyrase subunit (B) GyrB |  | -1.74 |  |
| 214 | 561 | *ezrA* | septation ring formation regulatory protein EzrA |  | -1.61 |  |
| 215 | 563 | *eno* | enolase protein Eno |  | 2.42 |  |
| 216 | 564 | *sagA* | streptolysin S precursor SagA |  | -2.72 |  |
| 217 | 565 | *sagB* | streptolysin S biosynthesis protein (B) SagB |  | -3.53 |  |
| 218 | 566 | *sagC* | streptolysin S biosynthesis protein (C) SagC |  | -3.84 |  |
| 219 | 567 | *sagD* | streptolysin S biosynthesis protein (D) SagD |  | -3.63 |  |
| 220 | 568 | *sagE* | streptolysin S self-immunity protein SagE |  | -4.47 |  |
| 221 | 569 | *sagF* | streptolysin S export protein SagF |  | -4.10 |  |
| 222 | 570 | *sagG* | streptolysin S export protein SagG |  | -4.67 |  |
| 223 | 571 | *sagH* | streptolysin S export permease protein SagH |  | -4.74 |  |
| 224 | 572 | *sagI* | streptolysin S export permease protein SagI |  | -4.03 |  |
| 225 | 573 |  | putative extracellular nuclease |  | -3.20 |  |
| 226 | 578 | *atpB* | ATP synthase A subunit AtpB |  | 1.59 |  |
| 227 | 591 |  | OsmC-like protein |  | -1.99 |  |
| 228 | 592 |  | hypothetical protein |  | -2.27 |  |
| 229 | 593 |  | ABC transport system permease protein |  | -1.89 |  |
| 230 | 594 |  | ABC transport system ATP-binding protein |  | -1.72 |  |
| 231 | 601 | *dnaG* | DNA primase protein DnaG |  | -2.06 |  |
| 232 | 602 | *rpoD* | RNA polymerase sigma factor RpoD |  | -2.07 |  |
| 233 | 603 |  | metal-sulfur cluster biosynthetic enzyme |  | -2.47 |  |
| 234 | 604 | *rmlD* | dTDP-4-dehydrorhamnose reductase protein RmlD |  | -1.58 |  |
| 235 | 618 |  | ferredoxin | 1.92 |  |  |
| 236 | 625 |  | methyltransferase |  | -1.58 |  |
| 237 | 634 |  | putative poly-gamma-glutatmate biosynthesis protein CapA |  | 1.85 |  |
| 238 | 645 | *carB* | carbamoyl-phosphate synthase large subunit CarB |  | -1.65 |  |
| 239 | 646 |  | RND family transporter membrane fusion protein | 2.65 |  |  |
| 240 | 647 |  | ABC transport system ATP-binding protein | 2.27 |  |  |
| 241 | 648 |  | ABC transport system ATP-binding/permease protein | 2.18 | -1.62 |  |
| 242 | 649 |  | glycerophosphoryl diester phosphodiesterase | 2.00 | -2.44 |  |
| 243 | 650 | *rpsP* | SSU ribosomal protein (S16p) RpsP |  | -1.76 |  |
| 244 | 651 |  | RNA-binding protein |  | -1.95 |  |
| 245 | 656 | *rimM* | 16S rRNA processing protein RimM |  | 1.60 |  |
| 246 | 657 | *trmD* | tRNA (Guanine37-N1)-methyltransferase protein TrmD |  | 1.96 |  |
| 247 | 658 | *trxB.1* | thioredoxin reductase protein TrxB | 1.76 | 2.61 |  |
| 248 | 659 |  | putative toxin regulator | 2.01 |  |  |
| 249 | 660 | *apbA* | 2-dehydropantoate 2-reductase protein ApbA | 1.57 |  |  |
| 250 | 661 | *fruR* | fructose operon transcriptional repressor protein FruR | 1.93 | 3.11 |  |
| 251 | 662 | *fruK* | 1-phosphofructokinase protein FruK | 2.22 | 3.21 |  |
| 252 | 663 | *fruA* | PTS system fructose-specific IIA-IIB-IIC component FruA |  | 1.56 |  |
| 253 | 665 |  | peptidoglycan hydrolase autolysin2 | 1.88 |  |  |
| 254 | 667 |  | SpeC-like protein | 1.62 |  |  |
| 255 | 668 | *ideS* | immunoglobulin G-endopeptidase protein IdeS/Mac1 | 1.58 |  |  |
| 256 | 669 | *ndk* | nucleoside diphosphate kinase protein Ndk | 1.70 |  |  |
| 257 | 676 | *ushA* | 5'-nucleotidase |  | -3.77 |  |
| 258 | 692 | *clpL* | ATP-dependent Clp proteinase protein ClpL | -1.66 | 3.07 |  |
| 259 | 697 | *deoD* | purine nucleoside phosphorylase protein DeoD |  | -1.62 |  |
| 260 | 700 |  | hypothetical protein |  | 1.65 |  |
| 261 | 709 | *parE* | topoisomerase IV subunit (B) ParE |  | 1.72 |  |
| 262 | 710 | *parC* | topoisomerase IV subunit (A) ParC |  | 2.13 |  |
| 263 | 711 | *bcaT* | branched-chain amino acid aminotransferase protein BcaT |  | 2.60 |  |
| 264 | 719 |  | hypothetical protein | -1.56 |  |  |
| 265 | 733 |  | hypothetical protein |  | 1.76 |  |
| 266 | 734 | *dadA* | glycine/D-amino acid oxidase protein DadA |  | 1.55 |  |
| 267 | 742 |  | ABC transporter substrate-binding protein |  | 1.52 |  |
| 268 | 744 |  | ABC transport system permease protein | 1.59 |  |  |
| 269 | 745 |  | ABC transport system ATP-binding protein | 1.79 | 1.78 |  |
| 270 | 768 | *csn1* | CRISPR-associated protein Csn1 |  | -1.56 |  |
| 271 | 769 | *cas1.1* | CRISPR-associated protein Cas1 |  | -1.74 |  |
| 272 | 775 | *msrB* | peptide methionine sulfoxide reductase protein MsrB | -1.50 |  |  |
| 273 | 777 | *manX* | PTS system IIA component ManX |  | -4.44 |  |
| 274 | 778 |  | PTS system IIB component |  | -4.24 |  |
| 275 | 779 | *manY* | PTS system IIC component ManY |  | -4.52 |  |
| 276 | 780 | *manZ* | PTS system mannose/fructose family IID component ManZ |  | -4.74 |  |
| 277 | 781 |  | TCS signal transduction sensor kinase |  | -2.72 |  |
| 278 | 782 |  | TCS signal transduction response regulator |  | -2.41 |  |
| 279 | 783 |  | ferric iron ABC transport system substrate-binding lipoprotein |  | -2.03 |  |
| 280 | 784 |  | succinate-semialdehyde dehydrogenase |  | -4.15 |  |
| 281 | 785 | *uvrC* | excinuclease ABC subunit (C) UvrC |  | -1.73 |  |
| 282 | 787 | *pepV* | Xaa-His dipeptidase protein PepV | 1.66 | 1.71 | RB03-04 |
| 283 | 788 | *trmE* | tRNA modification GTPase TrmE |  | 1.55 | RB03-04 |
| 284 | 789 | *rplJ* | LSU ribosomal protein (L10p) RplJ |  | 3.43 | RB03-04 |
| 285 | 790 | *rplL* | LSU ribosomal protein (L23e) RplL |  | 2.28 | RB03-04 |
| 286 | 793 | *srtI* | streptin immunity protein SrtI | 1.57 | -1.54 | RB03-04 |
| 287 | 794 | *srtR* | lantibiotic sysnthesis TCS signal transduction response regulator SrtR | 1.71 |  | RB03-04 |
| 288 | 795 | *srtK* | lantibiotic sysnthesis TCS signal transduction sensor kinase SrtK | 1.72 |  | RB03-04 |
| 289 | 805 | *dacC* | D-alanyl-D-alanine serine type carboxypeptidase DacC | 2.40 | 1.72 | RB03-04 |
| 290 | 806 |  | polysaccharide deacetylase | -1.63 |  | RB03-04 |
| 291 | 807 | *folC.2* | dihydrofolate synthase/folylpolyglutamate synthase protein FolC |  | 1.75 | RB03-04 |
| 292 | 808 | *folE* | GTP cyclohydrolase I protein FolE |  | 1.92 | RB03-04 |
| 293 | 809 | *folP* | dihydropteroate synthase protein FolP |  | 1.61 | RB03-04 |
| 294 | 812 | *murB* | UDP-N-acetylenolpyruvoylglucosamine reductase protein MurB |  | -1.65 | RB03-04 |
| 295 | 817 | *dpiA* | TCS signal transduction response regulator DpiA |  | -2.01 |  |
| 296 | 818 |  | maleic enzyme |  | -1.82 |  |
| 297 | 819 | *dpiB* | TCS signal transduction sensor histidine kinase DpiB |  | -3.01 |  |
| 298 | 820 |  | dehydrogenase |  | -2.49 |  |
| 299 | 821 | *aphA* | acid phosphatase/phosphotransferase protein AphA | 1.77 | -3.76 |  |
| 300 | 825 | *radC* | DNA repair protein RadC | -1.57 |  |  |
| 301 | 826 |  | glutamine amidotransferase |  | -1.61 |  |
| 302 | 836 |  | Short chain dehydrogenase |  | -2.73 |  |
| 303 | 837 |  | hypothetical protein |  | -2.60 |  |
| 304 | 838 |  | proline glycine betaine ABC transport system permease protein |  | -2.38 |  |
| 305 | 839 |  | glycine betaine ABC transport system permease protein |  | -2.56 |  |
| 306 | 841 | *xpt* | xanthine phosphoribosyltransferase protein Xpt | -1.66 |  |  |
| 307 | 842 | *pbuX* | xanthine permease protein PbuX | -1.51 |  |  |
| 308 | 852 |  | hypothetical protein |  | 1.53 |  |
| 309 | 856 | *ldh* | L-lactate dehydrogenase protein Ldh |  | 1.81 |  |
| 310 | 860 |  | hypothetical protein | 1.70 |  |  |
| 311 | 872 |  | hypothetical protein |  | -1.64 |  |
| 312 | 873 |  | hypothetical protein |  | -1.53 |  |
| 313 | 875 | *oadA.1* | oxaloacetate decarboxylase alpha chain, OadA | 2.54 | 2.61 |  |
| 314 | 876 |  | hypothetical protein | 4.79 | 3.59 |  |
| 315 | 877 |  | biotin carboxyl carrier protein of oxaloacetate decarboxylase |  | -1.75 |  |
| 316 | 884 | *oadB.2* | oxaloacetate decarboxylase beta chain, OadB | -1.72 | 3.55 |  |
| 317 | 885 |  | hypothetical protein |  | 4.47 |  |
| 318 | 886 | *citD* | citrate lyase gamma chain, CitD |  | 7.90 |  |
| 319 | 887 | *citE* | citrate lyase beta chain, CitE |  | 4.80 |  |
| 320 | 888 | *citF* | citrate lyase alpha chain, CitF |  | 4.79 |  |
| 321 | 889 | *citX* | apo-citrate lyase phosphoribosyl-dephospho-CoA transferase, CitX |  | 3.87 |  |
| 322 | 890 | *oadA.2* | oxaloacetate decarboxylase alpha chain, OadA |  | 4.56 |  |
| 323 | 894 |  | transcriptional regulator | -1.62 | -1.55 |  |
| 324 | 908 | *lplA.2* | lipoate-protein ligase A, LplA |  | -1.66 |  |
| 325 | 909 |  | ATPase associated with chromosome architecture/replication |  | -1.96 |  |
| 326 | 910 |  | hypothetical protein |  | -2.35 |  |
| 327 | 911 | *gcvH* | glycine cleavage system H protein, GcvH |  | -2.64 |  |
| 328 | 912 |  | luciferase-like monooxygenase |  | -2.54 |  |
| 329 | 913 |  | NADH:flavin oxidoreductase |  | -3.57 |  |
| 330 | 914 | *lplA.3* | lipoate-protein ligase A, LplA |  | -3.57 |  |
| 331 | 919 |  | putative nucleoside ABC transporter permease protein |  | -2.57 |  |
| 332 | 920 |  | putative nucleoside ABC transporter permease protein |  | -2.51 |  |
| 333 | 921 |  | putative nucleoside ABC transporter ATP-binding protein |  | -2.52 |  |
| 334 | 922 |  | putative nucleoside ABC transporter substrate-binding lipoprotein |  | -2.22 |  |
| 335 | 923 | *cdd* | cytidine deaminase, Cdd |  | -1.99 |  |
| 336 | 924 | *rsmC* | 16S rRNA methyltransferase, RsmC |  | -3.45 |  |
| 337 | 925 | *panK* | pantothenate kinase, PanK |  | -5.33 |  |
| 338 | 926 | *rpsT* | SSU ribosomal protein S20p, RpsT |  | -1.93 |  |
| 339 | 937 |  | inositol-1-monophosphatase | -1.62 | -2.03 | RB05 |
| 340 | 938 |  | hypothetical protein |  | -1.93 | RB05 |
| 341 | 939 | *SpxA* | transcriptional regulator, SpxA | -1.85 | -2.55 | RB05 |
| 342 | 941 | *truB* | tRNA pseudouridine synthase B, TruB | 1.80 | 1.78 | RB05 |
| 343 | 942 |  | hypothetical protein |  | 1.56 | RB05 |
| 344 | 943 |  | hypothetical protein |  | -1.73 | RB05 |
| 345 | 944 |  | type I restriction-modification system specificity subunit |  | -1.78 | RB05 |
| 346 | 945 |  | ABC transporter permease protein | -1.85 | -2.14 | RB05 |
| 347 | 946 |  | ABC transporter, ATP-binding protein | -1.64 | -2.23 | RB05 |
| 348 | 947 |  | transcriptional regulator |  | -2.05 | RB05 |
| 349 | 949 |  | transcriptional regulator |  | -1.86 | RB05 |
| 350 | 950 |  | hypothetical protein | 2.77 |  | RB05 |
| 351 | 951 |  | general stress response protein | 3.18 |  | RB05 |
| 352 | 952 |  | general stress protein | 2.68 |  | RB05 |
| 353 | 953 |  | hypothetical protein | 2.88 |  | RB05 |
| 354 | 954 |  | hypothetical protein | 3.16 |  | RB05 |
| 355 | 955 |  | hypothetical protein | 3.08 |  | RB05 |
| 356 | 957 | *alsT* | Na+/alanine symporter, AlsT | 1.86 |  | RB05 |
| 357 | 958 |  | hypothetical protein | 2.70 | -1.58 | RB05 |
| 358 | 959 |  | Cobalt-zinc-cadmium resistance/efflux protein, CzcD-like | 1.95 |  | RB05 |
| 359 | 960 | *cfa* | CAMP factor, Cfa | -2.04 | -1.60 | RB05 |
| 360 | 961 |  | ABC-type amino acid transport substrate-binding lipoprotein | -1.59 | -1.60 | RB05 |
| 361 | 964 | *pnhA* | alkylphosphonate utilization operon protein, PhnA | -1.54 |  | RB05 |
| 362 | 967 | *pyk* | pyruvate kinase Pyk |  | 1.86 | RB05 |
| 363 | 968 | *pfkA* | 6-phosphofructokinase, PfkA |  | 1.67 | RB05 |
| 364 | 969 | *dnaE* | DNA polymerase III alpha subunit, DnaE |  | 1.63 | RB05 |
| 365 | 975 |  | hypothetical protein |  | -1.64 |  |
| 366 | 976 | *glgP* | maltodextrin phosphorylase protein GlgP |  | -2.20 |  |
| 367 | 977 | *malQ* | 4-alpha-glucanotransferase (amylomaltase) protein MalQ |  | -2.06 |  |
| 368 | 978 | *malR* | maltose operon transcriptional repressor MalR |  | -1.65 |  |
| 369 | 979 | *malE* | maltose/maltodextrin ABC transporter substrate-binding lipoprotein MalE |  | -3.69 |  |
| 370 | 980 | *malF* | maltose/maltodextrin ABC transporter permease protein MalF |  | -3.90 |  |
| 371 | 981 | *malG* | maltose/maltodextrin ABC transporter permease protein MalG |  | -3.51 |  |
| 372 | 989 |  | transposase | -2.24 | -1.61 |  |
| 373 | 997 | *glnP* | glutamine ABC transporter permease protein GlnP |  | 1.80 |  |
| 374 | 998 | *glnQ.2* | glutamate transport ATP-binding protein GlnQ |  | 1.76 |  |
| 375 | 999 | *celB.1* | PTS system cellobiose specific IIC component CelB |  | -5.62 |  |
| 376 | 1000 |  | hypothetical protein |  | -4.40 |  |
| 377 | 1001 | *celC.1* | PTS system sugar cellobiose specific IIA component CelC |  | -4.14 |  |
| 378 | 1002 | *celA.1* | PTS system cellobiose-specific IIB component CelA |  | -3.93 |  |
| 379 | 1003 |  | transcriptional antiterminator of lichenan operon, BglG family |  | -3.12 |  |
| 380 | 1004 |  | hypothetical protein |  | -2.21 |  |
| 381 | 1005 | *bglB.2* | beta-glucosidase protein BglB |  | -1.79 |  |
| 382 | 1006 |  | PnuC-like ribosyl nicotinamide transporter |  | -1.85 |  |
| 383 | 1010 |  | IS861 transposase orfA | 2.41 |  |  |
| 384 | 1011 |  | IS861 transposase orfB | 1.84 |  |  |
| 385 | 1013 |  | putative thioesterase |  | -1.64 |  |
| 386 | 1024 |  | CBS domain containing putative transcriptional regulator |  | -1.55 |  |
| 387 | 1025 |  | RimL-like ribosomal protein acetyltransferase |  | -1.94 |  |
| 388 | 1026 | *graB* | protein G-related alpha 2 macroglobulin-binding protein GraB |  | 1.71 |  |
| 389 | 1028 | *metK* | S-adenosylmethionine synthetase protein MetK |  | 1.59 | RB06 |
| 390 | 1034 |  | putative proteinase |  | 1.55 |  |
| 391 | 1035 |  | hypothetical protein |  | 1.55 |  |
| 392 | 1036 | *udk* | uridine kinase protein Udk |  | 1.64 |  |
| 393 | 1039 | *gapN* | NADP-dependent glyceraldehyde-3-phosphate dehydrogenase GapN |  | 1.74 |  |
| 394 | 1042 | *nrdH* | glutaredoxin-like protein NrdH | -2.00 |  |  |
| 395 | 1043 | *nrdA* | ribonucleotide reductase alpha subunit NrdA | -1.91 | 1.76 |  |
| 396 | 1045 | *nrdF.2* | ribonucleotide reductase beta subunit NrdF | -1.71 |  |  |
| 397 | 1047 |  | transposase | 1.80 | 1.89 |  |
| 398 | 1052 | *alaS* | alanyl-tRNA synthetase protein AlaS |  | 1.51 |  |
| 399 | 1057 | *coiA* | competence protein CoiA | -1.60 | -1.58 |  |
| 400 | 1058 | *rsuA.3* | ribosomal SSU pseudouridine synthase A protein RsuA | -1.54 |  |  |
| 401 | 1059 | *nagB* | glucosamine-6-phosphate deaminase protein NagB | -1.77 |  |  |
| 402 | 1061 |  | hypothetical protein |  | 1.54 |  |
| 403 | 1062 |  | hypothetical protein | -3.27 | -2.08 |  |
| 404 | 1063 |  | hypothetical protein | -2.77 | -2.33 |  |
| 405 | 1064 |  | hypothetical protein | -3.14 | -2.57 |  |
| 406 | 1072 | *kup* | potassium uptake protein Kup | -1.64 |  |  |
| 407 | 1080 | *focA* | formate/nitrite family transport protein FocA | 1.52 | -2.83 |  |
| 408 | 1082 |  | putative transcriptional regulator |  | 2.03 |  |
| 409 | 1083 | *gpmA* | phosphoglycerate mutase protein GpmA |  | 2.07 |  |
| 410 | 1084 | *pyrD* | dihydroorotate dehydrogenase catalytic subunit PyrD | -1.57 |  |  |
| 411 | 1088 |  | hypothetical protein |  | -1.54 |  |
| 412 | 1093 | *argR.1* | arginine repressor ArgR | 1.68 |  |  |
| 413 | 1099 |  | phosphoglucomutase |  | -2.11 |  |
| 414 | 1100 | *phrB* | deoxyribodipyrimidine photolyase protein PhrB |  | 2.10 |  |
| 415 | 1102 |  | amino acid ABC transport system permease |  | -1.63 |  |
| 416 | 1103 |  | hypothetical protein |  | 1.62 |  |
| 417 | 1104 | *clpE* | ATP-dependent Clp protease ATP-binding subunit ClpE |  | 2.60 |  |
| 418 | 1105 | *mutT* | 7,8-dihydro-8-oxoguanine-triphosphatase/mutator protein MutT |  | 1.93 |  |
| 419 | 1107 | *ileS* | isoleucyl-tRNA synthetase protein IleS |  | 2.87 |  |
| 420 | 1114 | *ftsA* | cell division protein FtsA | 2.00 |  |  |
| 421 | 1118 |  | hypothetical protein |  | 1.93 |  |
| 422 | 1119 | *bipA* | GTP-binding protein BipA |  | 1.50 |  |
| 423 | 1122 |  | hypothetical protein | 1.75 |  |  |
| 424 | 1123 | *dps* | non-specific DNA-binding protein Dps/ferroxidase |  | 1.53 |  |
| 425 | 1124 |  | putative peptidase |  | 1.65 |  |
| 426 | 1125 | *rmlN* | ribosomal RNA large subunit methyltransferase (N) RmlN |  | 1.89 |  |
| 427 | 1126 |  | hypothetical protein |  | 1.76 |  |
| 428 | 1127 |  | PurR-like transcriptional regulator |  | 1.52 |  |
| 429 | 1132 | *arcC* | carbamate kinase protein ArcC | 1.56 | -6.33 |  |
| 430 | 1133 |  | Xaa-His dipeptidase protein PepV-like | 1.52 | -5.73 |  |
| 431 | 1134 | *arcD* | arginine/ornithine antiporter ArcD |  | -5.20 |  |
| 432 | 1135 | *arcB* | ornithine carbamoyltransferase protein ArcB |  | -3.73 |  |
| 433 | 1136 |  | acetyltransferase |  | -3.20 |  |
| 434 | 1137 | *arcA* | arginine deiminase protein ArcA |  | -3.54 |  |
| 435 | 1138 | *arcR* | transcriptional regulatory protein ArcR |  | -2.53 |  |
| 436 | 1139 | *argR.2* | arginine pathway regulatory protein ArgR |  | -3.15 |  |
| 437 | 1144 | *msrA/B* | peptide methionine sulfoxide reductase protein MsrA/B | -1.75 | -3.08 |  |
| 438 | 1145 | *tlpA* | thiol:disulfide interchange protein tlpA | -1.84 | -3.30 |  |
| 439 | 1146 | *ccdA* | cytochrome c-type biogenesis protein CcdA | -1.87 | -2.49 |  |
| 440 | 1147 | *cas2.2* | CRISPR-associated endoribonuclease Cas2 | 3.07 | 4.07 |  |
| 441 | 1148 | *cas1.2* | CRISPR-associated endonuclease Cas1 | 3.27 | 3.71 |  |
| 442 | 1149 | *cas4* | CRISPR-associated RecB family exonuclease Cas4 | 3.08 | 4.25 |  |
| 443 | 1150 | *cas7* | CRISPR-associated protein Cas7/Csd2 | 2.67 | 4.24 |  |
| 444 | 1151 | *cas8* | CRISPR/Cas system-associated protein Cas8c/Csd1 | 3.03 | 3.61 |  |
| 445 | 1152 | *cas5* | CRISPR/Cas system-associated protein Cas5 | 2.58 | 3.71 |  |
| 446 | 1153 | *cas3* | CRISPR/Cas system-associated helicase Cas3 | 2.56 | 3.41 |  |
| 447 | 1155 |  | hypothetical protein | 1.52 | 2.51 |  |
| 448 | 1156 |  | hypothetical protein | 2.28 | 3.50 |  |
| 449 | 1168 | *lacZ* | beta-galactosidase protein LacZ |  | -3.55 |  |
| 450 | 1169 |  | two component system response regulator TrxR |  | -4.25 |  |
| 451 | 1170 |  | two component system sensor kinase TrxS |  | -4.65 |  |
| 452 | 1171 |  | hypothetical protein |  | -4.30 |  |
| 453 | 1172 |  | sugar ABC transport system substrate-binding lipoprotein |  | -6.62 |  |
| 454 | 1173 |  | sugar ABC transport system permease |  | -6.03 |  |
| 455 | 1174 |  | sugar ABC transport system permease |  | -5.19 |  |
| 456 | 1175 |  | transcriptional regulator/sugar kinase |  | -3.72 |  |
| 457 | 1176 |  | hypothetical protein |  | -3.79 |  |
| 458 | 1177 | *bglB.3* | beta-glucosidase protein BglB |  | -4.16 |  |
| 459 | 1178 |  | beta-N-acetylglucosaminidase |  | -2.61 |  |
| 460 | 1179 |  | LacI-like tanscriptional regulator |  | -1.58 |  |
| 461 | 1180 |  | hypothetical protein |  | -1.99 |  |
| 462 | 1181 |  | alpha-mannosidase |  | -4.66 |  |
| 463 | 1184 | *recX* | DNA recombination regulatory protein recX |  | 1.52 |  |
| 464 | 1185 |  | hypothetical protein |  | -1.92 |  |
| 465 | 1204 | *cysM* | cysteine synthase protein CysK/CysM |  | 1.74 |  |
| 466 | 1205 |  | ribosomal protein S1 domain containing protein |  | 1.63 |  |
| 467 | 1206 |  | HAD-like hydrolase/cyclophilin type peptidyl-prolyl cis-trans isomerase |  | 1.51 |  |
| 468 | 1215 | *rpoZ* | DNA-directed RNA polymerase omega subunit RpoZ |  | 1.52 |  |
| 469 | 1229 | *recU* | recombination protein RecU |  | -1.63 |  |
| 470 | 1232 | *nadE* | NAD synthetase protein nadE |  | 1.51 |  |
| 471 | 1237 |  | ABC-type polar amino acid transport system ATPase component |  | 1.69 |  |
| 472 | 1240 | *mraY* | phospho-N-acetylmuramoyl-pentapeptide- translocase protein MraY |  | 1.54 |  |
| 473 | 1244 |  | hypothetical protein | 1.75 |  |  |
| 474 | 1245 |  | hypothetical protein | 1.62 |  |  |
| 475 | 1248 |  | hypothetical protein |  | -1.95 |  |
| 476 | 1249 |  | ABC transport system ATP-binding component |  | -2.27 |  |
| 477 | 1250 |  | putative bacteriocin immunity protein |  | -1.89 |  |
| 478 | 1252 | *tal* | transaldolaseprotein Tal |  | -6.28 |  |
| 479 | 1253 |  | putative transcriptional regulatory protein |  | -7.17 |  |
| 480 | 1254 |  | NAD(FAD)-dependent dehydrogenase |  | -6.82 |  |
| 481 | 1255 | *glpF.1* | glycerol uptake facilitator protein GlpF |  | -1.99 |  |
| 482 | 1256 | *glpA* | glycerol-3-phosphate dehydrogenase protein GlpA |  | -1.88 |  |
| 483 | 1257 | *glpK* | glycerol kinase protein GlpK | 1.51 |  |  |
| 484 | 1259 |  | hypothetical protein |  | 2.78 |  |
| 485 | 1260 |  | hypothetical protein |  | 2.34 |  |
| 486 | 1261 | *glyS* | glycyl-tRNA synthetase beta subunit GlyS |  | 1.74 |  |
| 487 | 1262 | *glyQ* | glycyl-tRNA synthetase alpha subunit GlyQ |  | 1.68 |  |
| 488 | 1267 |  | hypothetical protein |  | 1.51 |  |
| 489 | 1270 |  | HAD superfamily hydrolase |  | -2.55 |  |
| 490 | 1271 |  | hypothetical protein |  | -4.14 |  |
| 491 | 1272 | *lacD.1* | tagatose 1,6-diphosphate aldolase protein LacD |  | -7.08 |  |
| 492 | 1273 | *lacC.1* | tagatose-6-phosphate kinase/1-phosphofructokinase protein LacC |  | -5.60 |  |
| 493 | 1274 | *lacB.1* | galactose-6-phosphate isomerase protein LacB |  | -4.83 |  |
| 494 | 1275 | *lacA.1* | galactose-6-phosphate isomerase protein LacA |  | -4.23 |  |
| 495 | 1276 |  | PTS system EIIC component |  | -3.69 |  |
| 496 | 1277 |  | PTS system galactose-specific IIB component |  | -2.98 |  |
| 497 | 1278 |  | PTS system galactose-specific IIA component |  | -2.77 |  |
| 498 | 1279 | *lacR.1* | lactose phosphotransferase system repressor protein LacR |  | -1.59 |  |
| 499 | 1282 | *copA* | opper-translocating P-type ATPase protein CopA |  | -1.68 |  |
| 500 | 1283 | *copY* | copper transport operon transcriptional repressor protein CopY |  | -1.55 |  |
| 501 | 1284 |  | esterase/lipase | -1.67 | -2.05 |  |
| 502 | 1290 |  | hypothetical protein |  | -1.61 |  |
| 503 | 1292 | *trmB* | tRNA (guanine46-N7-)-methyltransferase protein TrmB |  | -1.56 |  |
| 504 | 1303 | *manL* | PTS system mannose-specific IIB & IIA component protein ManL |  | -3.14 |  |
| 505 | 1304 | *manM* | PTS system mannose-specific IIC component ManM |  | -2.60 |  |
| 506 | 1305 | *manN* | PTS system mannose-specific IID component manN |  | -2.19 |  |
| 507 | 1307 | *serS* | seryl-tRNA synthetase |  | -1.80 |  |
| 508 | 1308 | *accA* | acetyl-coenzyme A carboxyl transferase alpha subunit AccA |  | 4.40 |  |
| 509 | 1309 | *accD* | acetyl-coenzyme A carboxyl transferase beta subunit AccD |  | 4.45 |  |
| 510 | 1310 | *accC* | biotin carboxylase of acetyl-CoA carboxylase protein AccC |  | 4.82 |  |
| 511 | 1311 | *fabZ* | beta-hydroxyacyl-acyl carrier protein FabZ |  | 4.63 |  |
| 512 | 1312 | *accB* | biotin carboxyl carrier protein AccB |  | 5.29 |  |
| 513 | 1313 | *fabF* | 3-oxoacyl-[acyl-carrier-protein] synthase protein FabF |  | 4.14 |  |
| 514 | 1314 | *fabG.2* | 3-ketoacyl-(acyl-carrier-protein) reductase protein FabG |  | 4.72 |  |
| 515 | 1315 | *fabD* | malonyl CoA-acyl carrier protein transacylase FabD |  | 5.31 |  |
| 516 | 1316 | *fabK* | enoyl-[acyl-carrier-protein] reductase FabK |  | 5.03 |  |
| 517 | 1317 | *acpP* | acyl carrier protein AcpP |  | 1.95 |  |
| 518 | 1318 | *fabH* | 3-oxoacyl-[acyl-carrier-protein] synthase protein FabH |  | 2.15 |  |
| 519 | 1319 | *fabT* | transcriptional regulatory protein FabT |  | 1.69 |  |
| 520 | 1320 | *phaB* | enoyl-CoA hydratase protein PhaB |  | 1.97 |  |
| 521 | 1327 |  | phosphatase | 1.69 |  |  |
| 522 | 1329 |  | hypothetical protein | 1.61 |  |  |
| 523 | 1333 |  | pyruvate phosphate dikinase | -22.34 | -33.50 |  |
| 524 | 1334 |  | hypothetical protein | -19.37 | -54.08 |  |
| 525 | 1335 |  | nicotinamidase | -28.71 | -50.20 |  |
| 526 | 1336 | *codY* | GTP-sensing transcriptional pleiotropic repressor protein CodY |  | -1.62 |  |
| 527 | 1337 |  | protein/aminotransferase |  | 1.65 |  |
| 528 | 1341 |  | hypothetical protein | -1.54 |  |  |
| 529 | 1342 |  | putative ABC transport system ATP-binding protein | -1.64 |  |  |
| 530 | 1349 |  | ABC transport system ATP-binding protein |  | -1.98 |  |
| 531 | 1350 | *htsC* | heme ABC transport system ATP-binding protein htsC |  | -1.63 |  |
| 532 | 1351 | *htsB* | heme ABC transport system permease protein HtsB |  | -1.79 |  |
| 533 | 1352 | *htsA* | ABC transport system heme-binding lipoprotein HtsA |  | -1.63 |  |
| 534 | 1353 | *shp* | heme binding protein Shp | 1.63 | -1.78 |  |
| 535 | 1354 | *shr* | hemoprotein receptor Shr |  | -2.21 |  |
| 536 | 1362 | *endoS* | endo-beta-N-acetylglucosaminidase protein EndoS |  | -1.84 |  |
| 537 | 1363 |  | hypothetical protein |  | -2.28 |  |
| 538 | 1367 | *nusB* | transcription termination protein NusB |  | -1.62 |  |
| 539 | 1368 |  | putative stress response protein |  | -1.64 |  |
| 540 | 1369 | *efp* | translation elongation factor (P) Efp |  | -1.83 |  |
| 541 | 1370 | *comEB* | dCMP deaminase/late competence protein ComEB | -2.40 | -3.14 |  |
| 542 | 1371 | *pepP.2* | Xaa-Pro aminopeptidase (P) PepP | -2.30 | -4.16 |  |
| 543 | 1375 | *rpsR* | SSU ribosomal protein (S18p) RpsR |  | 2.64 |  |
| 544 | 1376 | *ssb.2* | single-stranded DNA-binding protein Ssb |  | 2.15 |  |
| 545 | 1377 | *rpsF* | SSU ribosomal protein (S6p) RpsF |  | 1.98 |  |
| 546 | 1381 | *trx2* | thioredoxin |  | 2.02 |  |
| 547 | 1382 |  | PAP2 superfamily phosphatase |  | 1.74 |  |
| 548 | 1384 | *cvpA* | colicin V production protein CvpA |  | 1.52 |  |
| 549 | 1385 |  | hypothetical protein |  | 1.65 |  |
| 550 | 1389 |  | hypothetical protein |  | -1.82 |  |
| 551 | 1390 | *dinP* | DNA polymerase IV protein DinP |  | 1.93 |  |
| 552 | 1391 | *pfl* | pyruvate formate-lyase protein Pfl |  | -1.60 |  |
| 553 | 1394 |  | hypothetical protein |  | 2.16 |  |
| 554 | 1395 | *glpF.2* | Glycerol uptake facilitator protein GlpF |  | 2.60 |  |
| 555 | 1396 |  | NorA-like major facillitator transporter |  | 1.85 |  |
| 556 | 1397 |  | CrP/Fnr family transcriptional regulator |  | 1.87 |  |
| 557 | 1398 | *pepXP* | Xaa-Pro dipeptidyl-peptidase protein PepXP |  | 1.82 |  |
| 558 | 1399 |  | XRE family transcriptional regulator | -1.57 |  |  |
| 559 | 1401 |  | hypothetical protein |  | 1.85 |  |
| 560 | 1409 | *phnF* | PhnF-like transcriptional regulator |  | 2.36 |  |
| 561 | 1415 |  | mRNA degradation ribonuclease |  | -1.91 |  |
| 562 | 1419 | *pgk* | phosphoglycerate kinase protein Pgk |  | 1.56 |  |
| 563 | 1423 |  | putative stress response protein |  | -1.53 |  |
| 564 | 1425 | *gatY* | fructose-bisphosphate aldolase class II protein GatY/Fba |  | 2.86 |  |
| 565 | 1433 |  | hypothetical protein | 1.87 |  |  |
| 566 | 1445 | *glxK* | glycerate kinase protein GlxK |  | 1.86 |  |
| 567 | 1446 | *hsdR* | type I restriction-modification system restriction subunit HsdR |  | 1.86 |  |
| 568 | 1447 | *hsdS* | type I restriction-modification system specificity subunit HsdS |  | 1.70 |  |
| 569 | 1448 | *hsdM* | type I restriction-modification system DNA-methylase subunit HsdM |  | 1.63 |  |
| 570 | 1452 | *salY* | salivaricin biosysnthesis ABC transport system permease component SalY |  | -1.60 |  |
| 571 | 1453 | *salX* | salivaricin biosysnthesis ABC transport system ATPase component SalX | 1.66 | -2.85 |  |
| 572 | 1454 | *salT* | salivaricin biosysnthesis double-glycine type export protein SalT | 1.65 | -3.35 |  |
| 573 | 1455 | *salB* | salvaricin biosynthesis cyclase SalB | 1.66 | -3.93 |  |
| 574 | 1456 | *salA* | salivaricin lantibiotic SalA |  | -10.66 |  |
| 575 | 1457 | *lacG* | 6-phospho-beta-galactosidase protein LacG |  | -10.20 |  |
| 576 | 1458 | *lacE* | PTS system lactose-specific fused IIBC component LacE | 1.59 | -7.33 |  |
| 577 | 1459 | *lacF* | PTS system lactose-specific component LacF | 1.77 | -5.65 |  |
| 578 | 1460 | *lacD.2* | tagatose 1,6-diphosphate aldolase protein LacD | 2.03 | -4.71 |  |
| 579 | 1461 | *lacC.2* | tagatose-6-phosphate kinase protein LacC | 1.91 | -3.80 |  |
| 580 | 1462 | *lacB.2* | galactose-6-phosphate isomerase LacB subunit | 1.90 | -3.21 |  |
| 581 | 1463 | *lacA.2* | galactose-6-phosphate isomerase LacA subunit | 1.68 | -3.03 |  |
| 582 | 1464 | *lacR.2* | lactose phosphotransferase system repressor protein LacR |  | -3.64 |  |
| 583 | 1465 |  | hypothetical protein |  | 1.55 |  |
| 584 | 1466 |  | hypothetical protein |  | 1.91 |  |
| 585 | 1474 |  | TrmH family tRNA/rRNA methyltransferase |  | 1.50 |  |
| 586 | 1475 |  | hypothetical protein |  | 2.74 |  |
| 587 | 1476 |  | ribonuclease III family protein |  | 1.59 |  |
| 588 | 1477 | *cysS* | cysteinyl-tRNA synthetase protein CysS |  | 1.58 |  |
| 589 | 1478 |  | hypothetical protein |  | 1.72 |  |
| 590 | 1483 |  | transaldolase |  | -5.26 |  |
| 591 | 1484 | *sgaT.2* | ascorbate-specific PTS system enzyme IIC component sgaT |  | -4.86 |  |
| 592 | 1485 | *sgaB.2* | ascorbate PTS system IIB component SgaB | 1.73 | -4.06 |  |
| 593 | 1486 | *bglG* | transcription antiterminator BglG |  | -4.90 |  |
| 594 | 1487 | *rpsO* | SSU ribosomal protein (S15p) RpsO |  | 1.86 |  |
| 595 | 1491 |  | putative flavoprotein protein |  | 1.51 |  |
| 596 | 1500 | *pulA* | pullulanase protein PulA |  | -4.88 |  |
| 597 | 1501 | *dexB* | glucan 1,6-alpha-glucosidase protein DexB |  | -2.44 |  |
| 598 | 1502 | *msmK* | multiple sugar ABC transport system ATP-binding protein MsmK |  | -2.35 |  |
| 599 | 1504 | *ska* | streptokinase protein Ska | 4.31 | 2.61 |  |
| 600 | 1505 | *dtd* | D-tyrosyl-tRNA(Tyr) deacylase protein Dtd |  | 2.07 |  |
| 601 | 1506 | *relA* | GTP pyrophosphokinase (p)ppGpp synthetase protein RelA |  | 1.68 |  |
| 602 | 1507 | *sclA* | collagen-like surface protein SclA | 2.48 | 3.68 |  |
| 603 | 1509 |  | endonuclease/exonuclease/phosphatase family protein | 1.67 | -4.49 |  |
| 604 | 1510 |  | PTS system maltose and glucose-specific fused IIABC component | 1.56 | -4.95 |  |
| 605 | 1511 | *rsmE* | ribosomal RNA SSU methyltransferase protein (E) RsmE | 1.61 | 1.64 |  |
| 606 | 1512 | *prmA* | ribosomal protein L11 methyltransferase protein PrmA |  | 1.89 |  |
| 607 | 1513 |  | hypothetical protein |  | 1.67 |  |
| 608 | 1515 | *trpG* | para-aminobenzoate synthase amidotransferase component TrpG |  | 1.63 |  |
| 609 | 1519 |  | N-acyltransferase (NAT) superfamily protein |  | -2.00 |  |
| 610 | 1520 |  | DNA topology modulation protein |  | -1.72 |  |
| 611 | 1523 | *dppA* | dipeptide-binding ABC transport system substrate-binding protein DppA |  | 1.87 |  |
| 612 | 1524 | *dppB* | dipeptide ABC transport system permease protein DppB |  | 2.20 |  |
| 613 | 1525 | *dppC* | dipeptide ABC transport system permease protein DppC |  | 2.24 |  |
| 614 | 1526 | *dppD* | dipeptide ABC transport system ATP-binding protein DppD |  | 2.15 |  |
| 615 | 1527 | *dppF* | dipeptide ABC transport system ATP-binding protein DppF |  | 1.94 |  |
| 616 | 1528 |  | hypothetical protein |  | 1.91 |  |
| 617 | 1531 | *fba* | fibronectin-binding protein Fba | -1.66 | -2.07 |  |
| 618 | 1532 | *scpA* | C5a peptidase protein ScpA |  | -1.64 | RB07 |
| 619 | 1533 | *enn* | M-like protein Enn | -1.72 |  | RB07 |
| 620 | 1534 | *emm* | antiphagocytic M protein Emm89.0 | -1.61 |  | RB07 |
| 621 | 1535 | *mrp* | Emm-related protein Mrp | 3.34 | 7.27 | RB07 |
| 622 | 1536 | *mga* | multiple virulence gene transcriptional activator protein Mga | -2.13 | -7.60 | RB07 |
| 623 | 1543 |  | ABC transport system ATP-binding component |  | 1.64 | RB07 |
| 624 | 1544 |  | efflux system MFP-like component |  | 1.99 | RB07 |
| 625 | 1546 |  | hypothetical protein |  | 1.85 |  |
| 626 | 1547 | *sof* | serum opacity factor Sof |  | 1.66 |  |
| 627 | 1549 | *ropA* | peptidylproline cis-trans-isomerase protein PrsA/RopA | 2.79 |  |  |
| 628 | 1550 |  | hypothetical protein |  | -1.59 |  |
| 629 | 1555 | *ropB* | Rgg-like regulator of proteinase protein RopB |  | 2.72 |  |
| 630 | 1556 | *sdaB* | streptodornase B protein SdaB |  | -3.01 |  |
| 631 | 1557 | *pgpA* | putative phosphatidylglycerophosphatase protein PgpA |  | 2.21 |  |
| 632 | 1558 | *gldA* | glycerol dehydrogenase protein GldA | -1.56 | -1.60 |  |
| 633 | 1559 | *mipB* | transaldolase protein MipB | -1.69 | -1.58 |  |
| 634 | 1560 | *pflD* | pyruvate formate-lyase protein PflD | -1.59 |  |  |
| 635 | 1561 | *celB.2* | PTS system cellobiose-specific IIC component CelB |  | -6.40 | RB08 |
| 636 | 1562 | *celA.2* | PTS system cellobiose-specific IIB component CelA | -1.80 | -4.96 | RB08 |
| 637 | 1563 | *celC.2* | PTS system cellobiose-specific IIA component CelC | -2.09 | -4.16 | RB08 |
| 638 | 1570 |  | putative translation initiation inhibitor |  | -1.64 | RB08 |
| 639 | 1571 |  | hypothetical protein |  | 1.66 | RB08 |
| 640 | 1572 | *rluA.2* | ribosomal large subunit pseudouridine synthase protein RluA |  | 1.73 | RB08 |
| 641 | 1574 | *pepD.2* | dipeptidase protein PepD | 1.66 | -2.72 | RB08 |
| 642 | 1576 |  | XRE-family ranscriptional regulator |  | -1.74 | RB08 |
| 643 | 1577 | *groEL* | heat shock protein 60 family chaperone GroEL | 1.69 | 3.95 | RB08 |
| 644 | 1578 | *groES* | heat shock protein 60 family co-chaperone GroES | 2.02 | 5.46 | RB08 |
| 645 | 1579 | *clpC* | ATP-dependent Clp protease ATP-binding subunit ClpC |  | 3.24 | RB08 |
| 646 | 1580 | *ctsR* | transcriptional regulator protein CtsR |  | 2.07 | RB08 |
| 647 | 1585 | *hutI* | imidazolonepropionase protein HutI |  | 1.81 |  |
| 648 | 1591 |  | putative cationic amino acid transporter protein |  | -2.29 |  |
| 649 | 1593 | *hutG* | formiminoglutamase protein HutG |  | 1.69 |  |
| 650 | 1594 |  | putative transcriptional regulator | 1.58 | -5.56 |  |
| 651 | 1595 | *rpsB* | SSU ribosomal protein (S2p) RpsB |  | 1.81 |  |
| 652 | 1598 | *treC* | trehalose-6-phosphate hydrolase protein treC | 3.45 | -8.32 |  |
| 653 | 1599 |  | PTS system trehalose-specific IIABC component | 3.68 | -9.74 |  |
| 654 | 1601 |  | hypothetical protein |  | -1.80 |  |
| 655 | 1608 |  | putative oxidoreductase | -1.50 |  |  |
| 656 | 1609 |  | hypothetical protein | -1.88 |  |  |
| 657 | 1610 | *nrdD* | ribonucleotide reductase large subunit NrdD | -1.72 |  |  |
| 658 | 1624 | *argR.3* | arginine repressor protein ArgR |  | -1.61 |  |
| 659 | 1634 | *cadD* | cadmium resistance protein CadD | -1.52 |  |  |
| 660 | 1635 | *cadX* | cadmium efflux putative regulatory protein CadX | -1.57 |  |  |
| 661 | 1638 |  | hypothetical protein | -1.63 |  |  |
| 662 | 1639 |  | hypothetical protein |  | 1.57 |  |
| 663 | 1641 |  | hypothetical protein |  | 1.57 |  |
| 664 | 1642 |  | MutT/nudix family phosphohydrolase |  | 2.02 |  |
| 665 | 1643 |  | PadR family transcriptional regulator | -2.71 |  |  |
| 666 | 1644 |  | hypothetical protein | -4.77 |  |  |
| 667 | 1645 |  | hypothetical protein | -4.09 |  |  |
| 668 | 1651 | *dnaC* | replicative DNA helicase protein DnaC | 1.59 |  |  |
| 669 | 1652 | *rplI* | LSU ribosomal protein (L9p) rplI |  | 1.82 |  |
| 670 | 1653 |  | putative signaling protein | 1.51 | 1.56 |  |
| 671 | 1654 | *gidA.2* | tRNA uridine modification enzyme GidA |  | 4.31 |  |
| 672 | 1655 |  | MutT/nudix family hyrolase protein |  | 5.12 |  |
| 673 | 1656 | *trmU* | tRNA methyltransferase protein TrmU |  | 3.13 |  |
| 674 | 1657 | *sdhB* | L-serine dehydratase beta subunit SdhB |  | 2.48 |  |
| 675 | 1658 | *sdhA* | L-serine dehydratase alpha subunit SdhA |  | 2.12 |  |
| 676 | 1659 |  | putative transglycosylase | 3.30 | 1.81 |  |
| 677 | 1660 |  | CbiQ-like ABC transport system permease component |  | 1.70 |  |
| 678 | 1661 |  | CbiO-like ABC transport system ATPase component |  | 1.61 |  |
| 679 | 1662 |  | CbiO-like ABC transport system ATPase component |  | 1.71 |  |
| 680 | 1663 | *pgsA* | CDP-diacylglycerol--glycerol-3-P 3-phosphatidyltransferase PgsA |  | 1.63 |  |
| 681 | 1665 |  | Zn-dependent peptidase |  | 1.86 |  |
| 682 | 1666 |  | Zn-dependent peptidase |  | 1.54 |  |
| 683 | 1671 | *recF* | DNA recombination and repair protein RecF |  | 1.88 |  |
| 684 | 1673 | *guaB* | inosine-5'-monophosphate dehydrogenase protein GuaB |  | 2.00 |  |
| 685 | 1677 |  | Uup-like ABC transporter duplicated ATPase domain protein |  | 1.67 |  |
| 686 | 1683 | *rlmH* | LSU methyltransferase RlmH | -1.80 |  |  |

^a^ Locus tag designations are provided relative to clade 2 strain MGAS23530.

**Section 2: Genes differentially expressed between clade 2 strain MGAS23530 and clade 3 strain MGAS26844**

|  |  |  |  | **C3/C2**  **Fold Change** | |  |
| --- | --- | --- | --- | --- | --- | --- |
| **No.** | **Locus**  **Tag^a^** | **Gene** | **Product/Function** | **ME** | **ES** | **RB** |
| 1 | 37 |  | hypothetical protein | 1.73 |  |  |
| 2 | 45 | *purF* | amidophosphoribosyltransferase protein PurF |  | -1.63 |  |
| 3 | 47 | *purN* | phosphoribosylglycinamide formyltransferase protein PurN |  | -1.67 |  |
| 4 | 48 | *purH* | IMP cyclohydrolase protein PurH | | -1.65 |  |
| 5 | 50 | *purD* | phosphoribosylglycinamide synthetase |  | -1.57 |  |
| 6 | 61 | *adh2* | alcohol dehydrogenase/acetaldehyde dehydrogenase protein Adh2 | -1.98 |  |  |
| 7 | 62 | *adh1* | alcohol dehydrogenase protein Adh1 |  | 1.60 |  |
| 8 | 65 | *rplC* | LSU ribosomal protein (L3p) RplC |  | -1.62 |  |
| 9 | 68 | *rplB* | LSU ribosomal protein (L2p) RplB |  | -1.51 |  |
| 10 | 142 | *rofA* | transcriptional regulatory protein RofA | 3.44 | 2.15 |  |
| 11 | 148 | *tee/fctA* | T antigen/fimbrial major structural protein A |  | 1.59 |  |
| 12 | 149 | *srtB* | sortase B protein SrtB | 1.59 |  |  |
| 13 | 150 | *fctB* | fimbrial minor structural protein FctB | 1.60 |  |  |
| 14 | 160 |  | hypothetical protein |  | -1.56 |  |
| 15 | 176 | *nga* | nicotine adenine dinucleotide glycohydrolase protein Nga | 2.46 | 1.56 | RB10 |
| 16 | 177 | *ifs* | immunity factor protein Ifs | 2.16 |  | RB10 |
| 17 | 178 | *slo* | streptolysin O precursor protein Slo | 2.43 |  | RB10 |
| 18 | 179 |  | hypothetical protein |  | -5.30 | RB10 |
| 19 | 182 |  | hypothetical protein | 1.53 | -2.17 | RB10 |
| 20 | 194 | *opuAA* | glycine betaine ABC transporter ATP-binding protein OpuAA |  | -2.04 |  |
| 21 | 195 | *opuABC* | glycine betaine ABC transporter substrate-binding/permease protein OpuABC |  | -1.73 |  |
| 22 | 235 |  | hypothetical protein |  | 1.60 |  |
| 23 | 359 | *scpC* | serine endopeptidase protein ScpC/PrtS | -1.59 | -1.72 |  |
| 24 | 360 |  | hypothetical protein |  | -1.70 |  |
| 25 | 361 |  | hypothetical protein |  | -1.75 |  |
| 26 | 362 |  | hypothetical protein |  | -2.05 |  |
| 27 | 372 |  | transposase | -1.52 | -2.12 |  |
| 28 | 373 |  | transposase |  | -1.63 |  |
| 29 | 375 |  | hypothetical secreted protein | 1.56 |  |  |
| 30 | 376 |  | hypothetical secreted protein | 3.61 | 8.56 |  |
| 31 | 403 | *frr* | ribosome recycling factor protein Frr |  | 1.62 |  |
| 32 | 432 | *secG* | preprotein translocase subunit SecG |  | 1.56 |  |
| 33 | 448 |  | ABC transport system permease |  | 1.65 |  |
| 34 | 490 |  | putative transcription elongation metalloprotease |  | 1.52 |  |
| 35 | 500 |  | hypothetical protein |  | 2.29 |  |
| 36 | 526 |  | PTS system hyaluronate-oligosaccharide-specific IID component | -1.67 | -1.68 |  |
| 37 | 527 |  | PTS system hyaluronate-oligosaccharide-specific IIC component | -1.90 | -1.91 |  |
| 38 | 528 |  | PTS system hyaluronate-oligosaccharide-specific IIB component | -1.59 | -1.56 |  |
| 39 | 529 |  | unsaturated glucuronyl hydrolase | -2.01 | -1.87 |  |
| 40 | 530 |  | PTS system hyaluronate-oligosaccharide-specific IIA component | -1.99 |  |  |
| 41 | 531 |  | 5-keto-D-gluconate 5-reductase |  | -1.68 |  |
| 42 | 532 |  | putative 4-deoxy-L-threo-5- hexosulose-uronate ketol-isomerase |  | -1.67 |  |
| 43 | 533 | *kdgK* | 2-dehydro-3-deoxygluconate kinase protein KdgK |  | -1.67 |  |
| 44 | 534 | *kdgA* | 4-Hydroxy-2-oxoglutarate aldolase protein KgdA | | -1.79 |  |
| 45 | 541 |  | acetoin (diacetyl) reductase | | 1.80 |  |
| 46 | 548 | *pepD.1* | dipeptidase protein PepD | 1.83 | 4.30 |  |
| 47 | 549 | *acdA* | metal-binding protein AcdA |  | 1.70 |  |
| 48 | 564 | *sagA* | streptolysin S precursor SagA |  | 1.95 |  |
| 49 | 571 | *sagH* | streptolysin S export permease protein SagH |  | 1.56 |  |
| 50 | 572 | *sagI* | streptolysin S export permease protein SagI |  | 1.53 |  |
| 51 | 592 |  | hypothetical protein |  | 1.65 |  |
| 52 | 600 | *mscL* | large-conductance mechanosensitive channel protein MscL |  | 1.76 |  |
| 53 | 617 |  | hypothetical protein |  | 1.73 |  |
| 54 | 619 |  | hypothetical protein |  | 1.57 |  |
| 55 | 629 |  | glutathione reductase |  | 1.57 |  |
| 56 | 645 | *carB* | carbamoyl-phosphate synthase large subunit CarB |  | 1.51 |  |
| 57 | 763 |  | hypothetical protein |  | -1.78 |  |
| 58 | 772 |  | hypothetical protein | -1.50 |  |  |
| 59 | 774 | *sclB* | collagen-like surface protein (B) SclB | -1.74 | -1.57 |  |
| 60 | 777 | *manX* | PTS system IIA component ManX |  | 1.79 |  |
| 61 | 778 |  | PTS system IIB component |  | 1.88 |  |
| 62 | 779 | *manY* | PTS system IIC component ManY |  | 1.94 |  |
| 63 | 780 | *manZ* | PTS system mannose/fructose family IID component ManZ |  | 1.90 |  |
| 64 | 796 | *srtA* | streptin lantibiotic precursor SrtA | 7.12 | 4.84 |  |
| 65 | 797 | *srtT* | streptin lantibiotic ABC transporter permease SrtT | 5.97 | 4.48 |  |
| 66 | 798 | *srtC* | streptin lantibiotic synthesis cyclase SrtC | 6.56 | 3.86 |  |
| 67 | 799 | *srtB* | streptin lantibiotic synthesis serine/threoninedehydratase SrtB | 4.05 | 5.16 |  |
| 68 | 800 | *srtF* | streptin lantibiotic ABC transporter ATP-binding protein SrtF | 4.39 | 4.61 |  |
| 69 | 801 | *srtE* | streptin lantibiotic ABC transporter permease SrtE | 4.73 | 4.02 |  |
| 70 | 802 | *srtG* | streptin lantibiotic ABC transporter permease SrtG | 3.93 | 3.83 |  |
| 71 | 803 |  | hypothetical protein | 1.95 |  |  |
| 72 | 807 | *folC.2* | dihydrofolate synthase/folylpolyglutamate synthase protein FolC |  | 1.68 |  |
| 73 | 812 | *murB* | UDP-N-acetylenolpyruvoylglucosamine reductase protein MurB |  | -1.51 |  |
| 74 | 821 | *aphA* | acid phosphatase/phosphotransferase protein AphA |  | 1.55 |  |
| 75 | 841 | *xpt* | xanthine phosphoribosyltransferase protein Xpt |  | -1.74 |  |
| 76 | 842 | *pbuX* | xanthine permease protein PbuX |  | -1.72 |  |
| 77 | 843 | *apbE* | thiamin biosynthesis lipoprotein ApbE |  | -1.84 |  |
| 78 | 861 |  | putative lipid kinase |  | 1.55 |  |
| 79 | 862 | *hylIII* | hemolysin III protein HylIII |  | 1.51 |  |
| 80 | 905 |  | anaerobic ribonucleoside-triphosphate reductase, NrdD-like |  | 1.70 |  |
| 81 | 938 |  | hypothetical protein |  | 1.50 |  |
| 82 | 939 | *SpxA* | transcriptional regulator, SpxA |  | 1.93 |  |
| 83 | 949 |  | transcriptional regulator |  | 1.51 |  |
| 84 | 950 |  | hypothetical protein |  | 1.90 |  |
| 85 | 951 |  | general stress response protein |  | 1.62 |  |
| 86 | 952 |  | general stress protein |  | 1.81 |  |
| 87 | 953 |  | hypothetical protein |  | 1.65 |  |
| 88 | 954 |  | hypothetical protein |  | 1.67 |  |
| 89 | 955 |  | hypothetical protein |  | 1.97 |  |
| 90 | 962 |  | ABC-type polar amino acid transporter ATPase component |  | -1.73 |  |
| 91 | 963 |  | ABC-type amino acid transporter membrane-spanning permease |  | -1.61 |  |
| 92 | 975 |  | hypothetical protein | -1.52 | -1.81 |  |
| 93 | 979 | *malE* | maltose/maltodextrin ABC transporter substrate-binding lipoprotein MalE |  | 1.66 |  |
| 94 | 980 | *malF* | maltose/maltodextrin ABC transporter permease protein MalF |  | 1.52 |  |
| 95 | 997 | *glnP* | ABC transporter glutamine-binding protein/permease protein GlnP |  | -1.53 |  |
| 96 | 999 | *celB.1* | PTS system cellobiose specific IIC component CelB |  | 1.66 |  |
| 97 | 1000 |  | hypothetical protein |  | 1.71 |  |
| 98 | 1001 | *celC.1* | PTS system sugar cellobiose specific IIA component CelC |  | 1.65 |  |
| 99 | 1002 | *celA.1* | PTS system cellobiose-specific IIB component CelA |  | 1.70 |  |
| 100 | 1003 |  | transcriptional antiterminator of lichenan operon, BglG family |  | 1.56 |  |
| 101 | 1010 |  | IS861 transposase orfA | -1.78 | -1.64 |  |
| 102 | 1011 |  | IS861 transposase orfB | -1.75 | -1.60 |  |
| 103 | 1016 | *paaI* | phenylacetic acid degradation protein PaaI | 2.77 | 1.90 |  |
| 104 | 1026 | *graB* | protein G-related alpha 2 macroglobulin-binding protein GraB |  | -1.65 |  |
| 105 | 1057 | *coiA* | competence protein CoiA |  | -1.59 |  |
| 106 | 1058 | *rsuA.3* | ribosomal SSU pseudouridine synthase A protein RsuA |  | -1.85 |  |
| 107 | 1059 | *nagB* | glucosamine-6-phosphate deaminase protein NagB |  | -1.57 |  |
| 108 | 1102 |  | amino acid ABC transport system permease |  | -1.58 |  |
| 109 | 1132 | *arcC* | carbamate kinase protein ArcC |  | 1.88 |  |
| 110 | 1133 |  | Xaa-His dipeptidase protein PepV-like |  | 1.77 |  |
| 111 | 1134 | *arcD* | arginine/ornithine antiporter ArcD |  | 1.62 |  |
| 112 | 1139 | *argR.2* | arginine pathway regulatory protein ArgR |  | 1.58 |  |
| 113 | 1140 |  | hypothetical protein | 1.45 | 1.50 | Phage^b^ |
| 114 | 1141 |  | hypothetical protein |  | 2.26 | Phage^b^ |
| 115 | 1142 | *yesM* | TCS signal transduction sensor kinase protein YesM | 1.65 | 2.39 | Phage^b^ |
| 116 | 1143 | *yesN* | TCS signal transduction response regulator protein YesN | 1.75 | 2.58 | Phage^b^ |
| 117 | 1144 | *msrA/B* | peptide methionine sulfoxide reductase protein MsrA/B | 33.56 | 22.18 | Phage^b^ |
| 118 | 1145 | *tlpA* | thiol:disulfide interchange protein tlpA | 28.50 | 18.47 | Phage^b^ |
| 119 | 1146 | *ccdA* | cytochrome c-type biogenesis protein CcdA | 38.53 | 44.16 | Phage^b^ |
| 120 | 1147 | *cas2.2* | CRISPR-associated endoribonuclease Cas2 | -1.86 | -4.17 |  |
| 121 | 1148 | *cas1.2* | CRISPR-associated endonuclease Cas1 |  | -3.54 |  |
| 122 | 1149 | *cas4* | CRISPR-associated RecB family exonuclease Cas4 |  | -3.26 |  |
| 123 | 1150 | *cas7* | CRISPR-associated protein Cas7/Csd2 |  | -3.79 |  |
| 124 | 1151 | *cas8* | CRISPR/Cas system-associated protein Cas8c/Csd1 | -2.31 | -4.74 |  |
| 125 | 1152 | *cas5* | CRISPR/Cas system-associated protein Cas5 |  | -2.62 |  |
| 126 | 1153 | *cas3* | CRISPR/Cas system-associated helicase Cas3 | -2.32 | -2.70 |  |
| 127 | 1154 | *valS* | valyl-tRNA synthetase protein ValS |  | 1.58 |  |
| 128 | 1155 |  | hypothetical protein |  | -1.56 |  |
| 129 | 1161 | *aroA.2* | 3-deoxy-7-phosphoheptulonate synthase protein AroA |  | -1.66 |  |
| 130 | 1173 |  | sugar ABC transporter permease |  | 1.52 |  |
| 131 | 1177 | *bglB.3* | beta-glucosidase protein BglB |  | 1.71 |  |
| 132 | 1200 | *raiA* | ribosomal associated protein |  | 1.85 |  |
| 133 | 1214 | *priA* | helicase protein PriA |  | -1.98 |  |
| 134 | 1216 | *gmk* | guanylate kinase protein Gmk |  | -1.72 |  |
| 135 | 1245 |  | hypothetical protein |  | 1.59 |  |
| 136 | 1259 |  | hypothetical protein |  | 1.52 |  |
| 137 | 1272 | *lacD.1* | tagatose 1,6-diphosphate aldolase protein LacD |  | 2.04 |  |
| 138 | 1273 | *lacC.1* | tagatose-6-phosphate kinase/1-phosphofructokinase protein LacC |  | 1.72 |  |
| 139 | 1274 | *lacB.1* | galactose-6-phosphate isomerase protein LacB |  | 1.64 |  |
| 140 | 1275 | *lacA.1* | galactose-6-phosphate isomerase protein LacA |  | 1.54 |  |
| 141 | 1276 |  | PTS system EIIC component |  | 1.72 |  |
| 142 | 1277 |  | PTS system galactose-specific IIB component |  | 1.67 |  |
| 143 | 1278 |  | PTS system galactose-specific IIA component |  | 1.71 |  |
| 144 | 1281 | *copZ* | copper chaperone protein CopZ | -1.72 |  |  |
| 145 | 1284 |  | esterase/lipase |  | -1.59 |  |
| 146 | 1301 |  | permease |  | -1.68 |  |
| 147 | 1328 |  | hypothetical protein |  | 1.77 |  |
| 148 | 1340 | *ansB* | asparaginase protein AnsB | 1.50 |  |  |
| 149 | 1361 | *scrK* | fructokinase protein ScrK | -1.80 | -2.02 |  |
| 150 | 1362 | *endoS* | endo-beta-N-acetylglucosaminidase protein EndoS | -2.51 |  |  |
| 151 | 1363 |  | hypothetical protein | -2.67 | -1.96 |  |
| 152 | 1364 | *scrA* | PTS system sucrose-specific fused IIB/IIC/IIA component proetin ScrA | -2.50 |  |  |
| 153 | 1394 |  | hypothetical protein |  | -1.63 |  |
| 154 | 1396 |  | NorA-like major facillitator transporter | 1.84 |  |  |
| 155 | 1397 |  | CrP/Fnr family transcriptional regulator |  | -1.53 |  |
| 156 | 1406 | *deoC* | deoxyribose-phosphate aldolase protein DeoC |  | 1.55 |  |
| 157 | 1408 | *udp.2* | uridine phosphorylase protein Udp |  | 1.51 |  |
| 158 | 1456 | *salA* | salivaricin lantibiotic SalA |  | 2.20 |  |
| 159 | 1458 |  | PTS system lactose-specific fused IIBC component LacE | -1.53 |  | RB12^c^ |
| 160 | 1459 | *lacF* | PTS system lactose-specific component LacF | -1.88 |  | RB12^c^ |
| 161 | 1460 | *lacD.2* | tagatose 1,6-diphosphate aldolase protein LacD | -1.78 |  | RB12^c^ |
| 162 | 1461 | *lacC.2* | tagatose-6-phosphate kinase protein LacC | -1.68 |  | RB12^c^ |
| 163 | 1462 | *lacB.2* | galactose-6-phosphate isomerase LacB subunit | -1.79 |  | RB12^c^ |
| 164 | 1463 | *lacA.2* | galactose-6-phosphate isomerase LacA subunit | -1.74 |  | RB12^c^ |
| 165 | 1469 | *rplM* | LSU ribosomal protein (L13p) RplM |  | -1.61 |  |
| 166 | 1488 |  | hypothetical protein |  | -1.54 |  |
| 167 | 1500 | *pulA* | pullulanase protein PulA |  | 2.10 |  |
| 168 | 1501 | *dexB* | glucan 1,6-alpha-glucosidase protein DexB |  | 1.70 |  |
| 169 | 1502 | *msmK* | multiple sugar ABC transport system ATP-binding protein MsmK |  | 1.54 |  |
| 170 | 1504 | *ska* | streptokinase protein Ska |  | 1.55 |  |
| 171 | 1507 | *sclA* | collagen-like surface protein SclA |  | -1.88 |  |
| 172 | 1509 |  | endonuclease/exonuclease/phosphatase family protein |  | 1.66 |  |
| 173 | 1510 |  | PTS system maltose and glucose-specific fused IIABC component |  | 1.96 |  |
| 174 | 1517 |  | hypothetical protein |  | 2.21 |  |
| 175 | 1528 |  | hypothetical protein |  | 1.95 |  |
| 176 | 1531 | *fba* | fibronectin-binding protein Fba |  | -1.79 |  |
| 177 | 1532 | *scpA* | C5a peptidase protein ScpA |  | -2.39 |  |
| 178 | 1533 | *enn* | M-like protein Enn |  | -2.32 |  |
| 179 | 1534 | *emm89* | antiphagocytic M protein Emm89.0 |  | -2.01 |  |
| 180 | 1547 | *sof* | serum opacity factor Sof | -3.55 | -4.81 |  |
| 181 | 1550 |  | hypothetical protein |  | 1.54 |  |
| 182 | 1551 | *spi* | streptopain inhibitor protein Spi |  | 1.73 |  |
| 183 | 1552 | *speB* | strepotococcal cysteine protease (streptopain) protein SpeB |  | 1.56 |  |
| 184 | 1558 | *gldA* | glycerol dehydrogenase protein GldA |  | 1.52 |  |
| 185 | 1567 | *secE* | preprotein translocase subunit SecE |  | 1.55 |  |
| 186 | 1568 | *rpmG.2* | LSU ribosomal protein (L33p) RpmG |  | 1.72 |  |
| 187 | 1570 |  | putative translation initiation inhibitor |  | 2.13 |  |
| 188 | 1598 | *treC* | trehalose-6-phosphate hydrolase protein treC |  | 1.86 |  |
| 189 | 1599 |  | PTS system trehalose-specific IIABC component |  | 2.18 |  |
| 190 | 1602 |  | transcriptional regulator | -1.62 |  |  |
| 191 | 1618 | *tag* | DNA-3-methyladenine glycosylase protein Tag |  | 1.64 |  |
| 192 | 1620 |  | LmrP-like putative multidrug transporter |  | 1.51 |  |
| 193 | 1626 |  | putative bacteriocin immunnity protein |  | 1.60 |  |
| 194 | 1635 | *cadX* | cadmium efflux putative regulatory protein CadX |  | -1.65 |  |
| 195 | 1637 |  | FtsK/SpoIIIE family protein |  | -1.65 |  |
| 196 | 1643 |  | PadR family transcriptional regulator |  | 2.10 |  |
| 197 | 1644 |  | hypothetical protein |  | 2.14 |  |
| 198 | 1645 |  | hypothetical protein |  | 2.21 |  |
| 199 | 1654 | *gidA.2* | tRNA uridine 5-carboxymethylaminomethyl modification enzyme GidA |  | -1.67 |  |
| 200 | 1655 |  | MutT/nudix family hyrolase protein |  | -1.61 |  |
| 201 | 1665 |  | Zn-dependent peptidase |  | -1.63 |  |
| 202 | 1666 |  | Zn-dependent peptidase |  | -1.61 |  |
| 203 | 1671 | *recF* | DNA recombination and repair protein RecF |  | -2.08 | RB14 |
| 204 | 1681 |  | transposase |  | -1.66 |  |

^a^ Locus tag designations are provided relative to clade 2 strain MGAS23530.

^b^ These genes are part of a transcriptional unit likely altered in expression as a consequence of phage 26844.2 integration between *yesN* and *msrA/B*. This phage is present in C3 strain MGAS26844 but not in C2 strain MGAS23530.

^c^ These genes do not set in RB12, they are part of a transcriptional unit likely altered in expression as a direct consequence of the RB12 HGT event altering the adjacent regulator LacR.2.

**Section 3: Genes differentially expressed between clade 3 strain MGAS26844 and subclade 3D strain MGAS27520**

|  |  |  |  | **SC3D/C3**  **Fold Change** | |  |
| --- | --- | --- | --- | --- | --- | --- |
| **No.** | **Locus**  **Tag^a^** | **Gene** | **Product/Function** | **ME** | **ES** | **RB** |
| 1 | 38 |  | hypothetical protein |  | 1.58 |  |
| 2 | 61 | *adh2* | alcohol dehydrogenase/acetaldehyde dehydrogenase protein Adh2 |  | -2.33 |  |
| 3 | 85 | *secY* | preprotein translocase subunit SecY |  | 1.53 |  |
| 4 | 91 | *rpoA* | DNA-directed RNA polymerase alpha subunit RpoA |  | 1.55 |  |
| 5 | 92 | *rplQ* | LSU ribosomal protein (L17p) RplQ |  | 1.59 |  |
| 6 | 117 | *tyrS* | tyrosyl-tRNA synthetase protein TyrS |  | 1.77 |  |
| 7 | 139 |  | deoxyribonucleotide kinase |  | 1.55 |  |
| 8 | 141 |  | chaperonin/heat shock protein |  | 1.52 |  |
| 9 | 142 | *rofA* | transcriptional regulatory protein RofA | -3.51 | -1.95 |  |
| 10 | 145 | *srtC* | sortase protein SrtC |  | -1.53 |  |
| 11 | 146 | *cpa/fctA* | fimbrial minor sturctural protein Cpa/FctA | -1.51 |  |  |
| 12 | 147 |  | signal peptidase I protein SipA/LepA | -1.54 | -1.52 |  |
| 13 | 149 | *srtB* | sortase B protein SrtB | -1.53 |  |  |
| 14 | 150 |  | fimbrial minor structural protein FctB | -1.66 |  |  |
| 15 | 209 |  | RofA-like transcriptional regulator |  | 2.06 |  |
| 16 | 233 |  | N-acetylneuraminate transporter permease protein 1 |  | -1.55 |  |
| 17 | 292 | *atmB* | methionine ABC transporter substrate-binding lipoprotein AtmB |  | -1.57 |  |
| 18 | 293 | *atmD* | methionine ABC transporter ATP-binding protein AtmD |  | -1.54 |  |
| 19 | 294 | *atmE* | methionine ABC transporter permease protein AtmE |  | -1.69 |  |
| 20 | 331 |  | hypothetical protein |  | -1.55 |  |
| 21 | 345 | *upp* | uracil phosphoribosyltransferase protein Upp |  | 1.64 |  |
| 22 | 351* | *spyA* | C3 family ADP-ribosyl transferase toxin |  | -1.79 | RB15 |
| 23 | 359* |  | short-chain dehydrogenase |  | 2.18 | RB15 |
| 24 | 385 |  | NAD-dependent oxidoreductase |  | 2.38 | RB15 |
| 25 | 436 | *pcp* | pyrrolidone-carboxylate peptidase protein Pcp |  | 1.51 |  |
| 26 | 500 |  | hypothetical protein |  | -1.90 |  |
| 27 | 507 |  | phage lysin glycosyl hydrolase |  | 1.56 |  |
| 28 | 512 | *ppc* | phosphoenolpyruvate carboxylase protein Ppc |  | -1.61 |  |
| 29 | 541 |  | 2,3-butanediol dehydrogenase |  | -1.77 |  |
| 30 | 548 | *pepD.1* | dipeptidase protein PepD | -1.88 | -3.83 |  |
| 31 | 549 | *acdA* | metal-binding protein AcdA |  | 1.75 |  |
| 32 | 564 | *sagA* | streptolysin S precursor SagA |  | -1.65 |  |
| 33 | 565 | *sagB* | streptolysin S biosynthesis protein (B) SagB |  | -1.50 |  |
| 34 | 600 | *mscL* | large-conductance mechanosensitive channel protein MscL |  | -1.89 |  |
| 35 | 617 |  | hypothetical protein |  | -1.53 |  |
| 36 | 629 |  | glutathione reductase |  | -1.74 |  |
| 37 | 641 | *pyrR* | pyrimidine operon regulatory protein PyrR |  | 2.49 |  |
| 38 | 642 | *pyrP* | uracil permease protein PyrP |  | 2.35 |  |
| 39 | 643 | *pyrB* | aspartate carbamoyltransferase protein PyrB |  | 2.43 |  |
| 40 | 644 | *carA* | carbamoyl-phosphate synthase small subunit CarA |  | 2.40 |  |
| 41 | 645 | *carB* | carbamoyl-phosphate synthase large subunit CarB |  | 2.39 |  |
| 42 | 685 | *mvaS2* | hydroxymethylglutaryl-CoA synthase protein (2) MvaS2 |  | 1.61 |  |
| 43 | 692 | *clpL* | ATP-dependent Clp proteinase protein ClpL |  | -1.55 |  |
| 44 | 700 |  | hypothetical protein |  | -1.77 |  |
| 45 | 701 | *pyrF* | orotidine 5'-phosphate decarboxylase protein PyrF |  | 1.84 |  |
| 46 | 702 | *pyrE* | orotate phosphoribosyltransferase protein PyrE |  | 2.20 |  |
| 47 | 703 |  | putative amidase |  | 1.92 |  |
| 48 | 704 |  | ABC transporter substrate-binding lipoprotein |  | 1.69 |  |
| 49 | 705 |  | ABC transporter permease protein |  | 1.80 |  |
| 50 | 710 | *parC* | topoisomerase IV subunit (A) ParC |  | 1.54 |  |
| 51 | 715 | *rpsA* | SSU ribosomal protein (S1p) RpsA |  | 1.56 |  |
| 52 | 730 | *dnaD* | chromosome replication initiation protein DnaD |  | 1.52 |  |
| 53 | 731 | *nth* | endonuclease III protein Nth |  | 1.67 |  |
| 54 | 796 | *srtA* | streptin lantibiotic precursor SrtA | -7.04 | -3.68 |  |
| 55 | 797 | *srtT* | streptin lantibiotic ABC transporter permease SrtT | -3.97 | -4.50 |  |
| 56 | 798 | *srtC* | streptin lantibiotic synthesis cyclase SrtC | -4.85 | -4.04 |  |
| 57 | 799 | *srtB* | streptin lantibiotic synthesis serine/threoninedehydratase SrtB | -3.14 | -4.98 |  |
| 58 | 800 | *srtF* | streptin lantibiotic ABC transporter ATP-binding protein SrtF | -4.04 | -4.94 |  |
| 59 | 801 | *srtE* | streptin lantibiotic ABC transporter permease SrtE | -4.58 | -3.94 |  |
| 60 | 802 | *srtG* | streptin lantibiotic ABC transporter permease SrtG | -4.13 | -3.93 |  |
| 61 | 803 |  | hypothetical protein | -1.70 |  |  |
| 62 | 812 | *murB* | UDP-N-acetylenolpyruvoylglucosamine reductase protein MurB |  | 1.81 |  |
| 63 | 813 | *potA* | spermidine putrescine ABC transporter ATP-binding protein PotA |  | 1.62 |  |
| 64 | 814 | *potB* | spermidine putrescine ABC transporter permease protein PotB |  | 1.62 |  |
| 65 | 815 | *potC* | spermidine putrescine ABC transporter permease protein PotC |  | 1.65 |  |
| 66 | 820 |  | dehydrogenase |  | -1.57 |  |
| 67 | 860 |  | hypothetical protein |  | -1.61 |  |
| 68 | 874 | *gidA.1* | glucose inhibited division protein A, GidA |  | 1.73 |  |
| 69 | 905 |  | Anaerobic ribonucleoside-triphosphate reductase, NrdD-like |  | -1.66 |  |
| 70 | 949 |  | Transcriptional regulator |  | -1.70 |  |
| 71 | 950 |  | hypothetical protein |  | -1.96 |  |
| 72 | 951 |  | general stress response protein |  | -1.88 |  |
| 73 | 952 |  | general stress protein |  | -2.15 |  |
| 74 | 953 |  | hypothetical protein |  | -1.99 |  |
| 75 | 954 |  | hypothetical protein |  | -1.85 |  |
| 76 | 955 |  | hypothetical protein |  | -1.81 |  |
| 77 | 971 |  | ABC-type multidrug transporter ATPase component |  | -1.50 |  |
| 78 | 972 |  | ABC transporter, ATP-binding protein |  | -1.86 |  |
| 79 | 1000 |  | hypothetical protein |  | -1.83 |  |
| 80 | 1001 | *celC.1* | PTS system sugar cellobiose specific IIA component CelC |  | -1.73 |  |
| 81 | 1002 | *celA.1* | PTS system cellobiose-specific IIB component CelA |  | -1.79 |  |
| 82 | 1003 |  | Transcriptional antiterminator of lichenan operon, BglG family |  | -1.61 |  |
| 83 | 1004 |  | hypothetical protein |  | -1.73 |  |
| 84 | 1005 | *bglB.2* | beta-glucosidase protein BglB |  | -1.50 |  |
| 85 | 1016 | *paaI* | phenylacetic acid degradation protein PaaI | -2.95 |  |  |
| 86 | 1048 |  | CAAX family protease |  | -1.59 |  |
| 87 | 1050 |  | XRE family transcriptional regulator |  | -1.57 |  |
| 88 | 1051 |  | hypothetical protein |  | -1.75 |  |
| 89 | 1054 |  | putative O-methyltransferase |  | 1.51 |  |
| 90 | 1060 | *queA* | queuosine synthase protein QueA |  | 1.52 |  |
| 91 | 1061 |  | hypothetical protein |  | 1.53 |  |
| 92 | 1073 | *srmB.2* | RNA helicase SrmB |  | -1.54 |  |
| 93 | 1134 | *arcD* | arginine/ornithine antiporter ArcD |  | -1.51 |  |
| 94 | 1136 |  | acetyltransferase |  | -1.50 |  |
| 95 | 1141 |  | hypothetical protein | -1.54 | -2.56 | Phage^b^ |
| 96 | 1142 | *yesM* | TCS signal transduction sensor kinase protein YesM | -1.65 | -2.90 | Phage^b^ |
| 97 | 1143 | *yesN* | TCS signal transduction response regulator protein YesN | -1.68 | -2.88 | Phage^b^ |
| 98 | 1144 | *msrA/B* | peptide methionine sulfoxide reductase protein MsrA/B | -26.69 | -12.86 | Phage^b^ |
| 99 | 1145 | *tlpA* | thiol:disulfide interchange protein tlpA | -17.47 | -11.57 | Phage^b^ |
| 100 | 1146 | *ccdA* | cytochrome c-type biogenesis protein CcdA | -18.06 | -32.59 | Phage^b^ |
| 101 | 1147 | *cas2.2* | CRISPR-associated endoribonuclease Cas2 |  | 2.13 |  |
| 102 | 1148 | *cas1.2* | CRISPR-associated endonuclease Cas1 | -1.65 | 1.63 |  |
| 103 | 1149 | *cas4* | CRISPR-associated RecB family exonuclease Cas4 | -2.13 | 1.64 |  |
| 104 | 1150 | *cas7* | CRISPR-associated protein Cas7/Csd2 | -2.25 | 1.61 |  |
| 105 | 1151 | *cas8* | CRISPR/Cas system-associated protein Cas8c/Csd1 |  | 2.12 |  |
| 106 | 1152 |  | CRISPR/Cas system-associated protein Cas5 | -1.98 |  |  |
| 107 | 1161 | *aroA.2* | 3-deoxy-7-phosphoheptulonate synthase protein AroA |  | 2.03 |  |
| 108 | 1162 | *aroB* | 3-dehydroquinate synthase protein AroB |  | 1.76 |  |
| 109 | 1172 |  | sugar ABC transporter substrate-binding lipoprotein |  | -1.57 |  |
| 110 | 1173 |  | sugar ABC transporter permease |  | -1.56 |  |
| 111 | 1177 | *bglB.3* | beta-glucosidase protein BglB |  | -1.62 |  |
| 112 | 1178 |  | beta-N-acetylglucosaminidase |  | -1.61 |  |
| 113 | 1180 |  | hypothetical protein |  | -1.57 |  |
| 114 | 1181 |  | alpha-mannosidase |  | -1.57 |  |
| 115 | 1200 |  | ribosomal associated protein |  | -1.62 |  |
| 116 | 1213 | *fmt* | methionyl-tRNA formyltransferase protein Fmt |  | -1.61 |  |
| 117 | 1215 | *rpoZ* | DNA-directed RNA polymerase omega subunit RpoZ |  | 1.58 |  |
| 118 | 1216 | *gmk* | guanylate kinase protein Gmk |  | 1.96 |  |
| 119 | 1255 | *glpF.1* | glycerol uptake facilitator protein GlpF |  | -1.52 |  |
| 120 | 1259 |  | hypothetical protein |  | -1.89 |  |
| 121 | 1263 |  | hypothetical protein |  | -1.56 |  |
| 122 | 1272 | *lacD.1* | tagatose 1,6-diphosphate aldolase protein LacD |  | -1.91 |  |
| 123 | 1273 | *lacC.1* | tagatose-6-phosphate kinase/1-phosphofructokinase protein LacC |  | -1.77 |  |
| 124 | 1274 | *lacB.1* | galactose-6-phosphate isomerase protein LacB |  | -1.85 |  |
| 125 | 1275 | *lacA.1* | galactose-6-phosphate isomerase protein LacA |  | -1.72 |  |
| 126 | 1276 |  | PTS system EIIC component |  | -2.00 |  |
| 127 | 1277 |  | PTS system galactose-specific IIB component |  | -2.03 |  |
| 128 | 1278 |  | PTS system galactose-specific IIA component |  | -1.79 |  |
| 129 | 1279 | *lacR.1* | lactose phosphotransferase system repressor protein LacR |  | -1.68 |  |
| 130 | 1312 | *accB* | biotin carboxyl carrier protein AccB |  | 1.53 |  |
| 131 | 1315 | *fabD* | malonyl CoA-acyl carrier protein transacylase FabD |  | 1.60 |  |
| 132 | 1316 | *fabK* | enoyl-[acyl-carrier-protein] reductase FabK |  | 1.56 |  |
| 133 | 1317 | *acpP* | acyl carrier protein AcpP |  | 1.64 |  |
| 134 | 1321 | *dnaJ* | chaperone protein DnaJ |  | -1.70 |  |
| 135 | 1324 | *hrcA* | heat-inducible transcription repressor HrcA |  | -1.57 |  |
| 136 | 1363 |  | hypothetical protein |  | -1.53 |  |
| 137 | 1364 |  | PTS system sucrose-specific fused IIB/C/A component proetin ScrA | 1.50 |  |  |
| 138 | 1373 | *corA* | magnesium and cobalt transport protein CorA |  | 1.59 |  |
| 139 | 1374 |  | hypothetical protein |  | 1.57 |  |
| 140 | 1406 | *deoC* | deoxyribose-phosphate aldolase protein DeoC |  | -1.58 |  |
| 141 | 1408 | *udp.2* | uridine phosphorylase protein Udp |  | -1.72 |  |
| 142 | 1430 | *tig* | cell division trigger factor protein Tig |  | 1.55 |  |
| 143 | 1456 | *salA* | salivaricin lantibiotic SalA |  | -1.70 |  |
| 144 | 1457 | *lacG* | 6-phospho-beta-galactosidase protein LacG |  | -1.60 |  |
| 145 | 1458 | *lacE* | PTS system lactose-specific fused IIBC component LacE |  | -1.74 |  |
| 146 | 1459 | *lacF* | PTS system lactose-specific component LacF |  | -1.62 |  |
| 147 | 1460 | *lacD.2* | tagatose 1,6-diphosphate aldolase protein LacD |  | -1.79 |  |
| 148 | 1461 | *lacC.2* | tagatose-6-phosphate kinase protein LacC |  | -1.50 |  |
| 149 | 1465 |  | hypothetical protein |  | -1.89 |  |
| 150 | 1466 |  | hypothetical protein |  | -1.99 |  |
| 151 | 1468 | *rpsI* | SSU ribosomal protein (S9p) RpsI |  | 1.73 |  |
| 152 | 1469 | *rplM* | LSU ribosomal protein (L13p) RplM |  | 1.68 |  |
| 153 | 1484 | *sgaT.2* | ascorbate-specific PTS system enzyme IIC component sgaT |  | -1.60 |  |
| 154 | 1485 | *sgaB.2* | ascorbate PTS system IIB component SgaB |  | -1.64 |  |
| 155 | 1509 |  | endonuclease/exonuclease/phosphatase family protein |  | -1.76 |  |
| 156 | 1517 |  | hypothetical protein |  | -1.67 |  |
| 157 | 1521 | *smeZ* | mitogenic exotoxin Z SmeZ/pyrogenic exotoxin X SpeX | -1.82 |  |  |
| 158 | 1522 |  | hypothetical protein | -1.92 | -1.68 |  |
| 159 | 1528 |  | hypothetical protein |  | -1.61 |  |
| 160 | 1547 | *sof* | serum opacity factor Sof |  | 1.80 |  |
| 161 | 1549 | *ropA/prsA* | peptidylproline cis-trans-isomerase protein PrsA/RopA |  | -1.87 |  |
| 162 | 1550 |  | hypothetical protein |  | -1.88 |  |
| 163 | 1551 | *spi* | streptopain inhibitor protein Spi |  | -2.13 |  |
| 164 | 1552 | *speB* | strepotococcal cysteine protease (streptopain) protein SpeB |  | -2.33 |  |
| 165 | 1554 |  | hypothetical protein |  | -1.96 |  |
| 166 | 1556 | *sdaB* | streptodornase B protein SdaB |  | -1.68 |  |
| 167 | 1558 | *gldA* | glycerol dehydrogenase protein GldA |  | -1.77 |  |
| 168 | 1559 | *mipB* | transaldolase protein MipB |  | -1.66 |  |
| 169 | 1560 | *pflD* | pyruvate formate-lyase protein PflD |  | -1.71 |  |
| 170 | 1561 | *celB.2* | PTS system cellobiose-specific IIC component CelB |  | -1.52 |  |
| 171 | 1563 | *celC.2* | PTS system cellobiose-specific IIA component CelC |  | -1.70 |  |
| 172 | 1570 |  | putative translation initiation inhibitor |  | -1.73 |  |
| 173 | 1577 | *groEL* | heat shock protein 60 family chaperone GroEL |  | -1.80 |  |
| 174 | 1578 | *groES* | heat shock protein 60 family co-chaperone GroES |  | -1.66 |  |
| 175 | 1598 | *treC* | trehalose-6-phosphate hydrolase protein treC |  | -1.77 |  |
| 176 | 1599 |  | PTS system trehalose-specific IIABC component |  | -1.64 |  |
| 177 | 1606 | *nrdG* | ribonucleotide reductase activating protein NrdG |  | -1.52 |  |
| 178 | 1608 |  | putative oxidoreductase |  | -1.50 |  |
| 179 | 1639 |  | hypothetical protein |  | -2.31 |  |
| 180 | 1640 |  | hypothetical protein |  | -2.47 |  |
| 181 | 1641 |  | hypothetical protein |  | -1.54 |  |
| 182 | 1642 |  | MutT/nudix family phosphohydrolase |  | -2.05 |  |
| 183 | 1643 |  | PadR family transcriptional regulator | -1.85 | -15.21 |  |
| 184 | 1644 |  | hypothetical protein | -2.89 | -13.55 |  |
| 185 | 1645 |  | hypothetical protein | -2.61 | -15.26 |  |
| 186 | 1646 |  | hypothetical protein |  | -2.33 |  |
| 187 | 1647 |  | transcriptional regulator |  | -1.92 |  |
| 188 | 1655 |  | MutT/nudix family hyrolase protein |  | 1.53 |  |
| 189 | 1681 |  | transposase |  | -1.89 |  |

^a^ Locus tag designations are provided relative to clade 2 strain MGAS23530, with the exception of tags 351 and 359 in RB15 which are relative to M1 strain MGAS5005 as these genes were horizontally acquired and cognate genes are not present in MGAS23530.

^b^ These genes are part of a transcriptional unit likely altered in expression as a consequence of phage 26844.2 integration between *yesN* and *msrA/B*. This phage is present in C3 strain MGAS26844 but not in SC3’ strain MGAS27520.

**Section 4: Genes differentially expressed between strains MGAS27556 WT and MGAS27556 LiaS (K214R)**

|  |  | | |  | | |  | | **LiaS(K214R)/WT**  **Fold Change** | | | | |  |  |
| --- | --- | --- | --- | --- | --- | --- | --- | --- | --- | --- | --- | --- | --- | --- | --- |
| **No.** | | | **Locus**  **Tag^a^** | | | **Gene** | | **Product/Function** | | **ME** | | | **ST** | | |
| 1 | | | 1 | | | *dnaA* | | | chromosomal replication initiator protein DnaA | | | | 1.63 | | |
| 2 | | | 38 | | |  | | | hypothetical protein | | | | -1.52 | | |
| 3 | | | 43 | | | *purC* | | | phosphoribosylaminoimidazole-succinocarboxamide synthase protein PurC | | | 2.40 |  | | |
| 4 | | | 44 | | | *purL* | | | phosphoribosylformylglycinamidine synthase protein PurL | | | 2.03 |  | | |
| 5 | | | 45 | | | *purF* | | | amidophosphoribosyltransferase protein PurF | | | 1.55 |  | | |
| 6 | | | 49 | | |  | | | phosphoribosylglycinamide synthetase | | | | -1.62 | | |
| 7 | | | 61 | | | *adh2* | | | alcohol dehydrogenase/acetaldehyde dehydrogenase protein Adh2 | | | 1.59 |  | | |
| 8 | | | 62 | | | *adh1* | | | alcohol dehydrogenase protein Adh1 | | | 2.30 |  | | |
| 9 | | | 64 | | | *rpsJ* | | | SSU ribosomal protein (S10p) RpsJ | | | | 1.50 | | |
| 10 | | | 187 | | | *sgaA* | | | ascorbate-specific PTS system EIIA protein SgaA | | | 1.90 |  | | |
| 11 | | | 188 | | | *ulaD* | | | 3-keto-L-gulonate 6-phosphate decarboxylase protein UlaD | | | 1.69 |  | | |
| 12 | | | 189 | | |  | | | L-xylulose 5-phosphate 3-epimerase | | | 2.01 |  | | |
| 13 | | | 190 | | |  | | | L-ribulose-5-phosphate 4-epimerase | | | 1.65 |  | | |
| 14 | | | 191 | | |  | | | hypothetical protein | | | 1.70 |  | | |
| 15 | | | 194 | | | *opuAA* | | | glycine betaine ABC transport system ATP-binding protein OpuAA | | | | 1.69 | | |
| 16 | | | 195 | | | *opuABC* | | | glycine betaine ABC transport system substrate-binding/permease protein OpuABC | | | | 1.59 | | |
| 17 | | | 212 | | |  | | | hypothetical protein | | | | 1.70 | | |
| 18 | | | 213 | | |  | | | BioY family protein | | | | 1.52 | | |
| 19 | | | 217 | | | *speG* | | | pyrogenic exotoxin protein (G) SpeG | | | | 1.60 | | |
| 20 | | | 301 | | | *atmA* | | | cysteine ABC transport system substrate-binding lipoprotein AtmA | | | -1.74 |  | | |
| 21 | | | 356 | | | *clpP* | | | ATP-dependent Clp protease proteolytic subunit ClpP | | | | 1.66 | | |
| 22 | | | 394 | | | *glpT* | | | phosphoglycerate transporter protein GlpT | | | 1.82 |  | | |
| 23 | | | 441 | | | *vacB* | | | 3'-to-5' exoribonuclease protein VacB | | | | 1.59 | | |
| 24 | | | 442 | | | *smpB* | | | tmRNA-binding protein SmpB | | | | 1.56 | | |
| 25 | | | 443 | | |  | | | glutaminyl-peptide cyclotransferase | | | | 1.52 | | |
| 26 | | | 455 | | |  | | | ABC transport system ATP-binding protein | | | 2.29 |  | | |
| 27 | | | 456 | | |  | | | ABC transport system permease | | | 1.97 |  | | |
| 28 | | | 457 | | |  | | | ABC transport system permease | | | 2.11 |  | | |
| 29 | | | 482 | | | *relB* | | | plasmid stabilization system antitoxin protein RelB | | | | 1.61 | | |
| 30 | | | 515 | | |  | | | phage lysin glycosyl hydrolase | | | | 1.61 | | |
| 31 | | | 599 | | | *sagA* | | | streptolysin S precursor SagA | | | 1.66 |  | | |
| 32 | | | 600 | | | *sagB* | | | streptolysin S biosynthesis protein (B) SagB | | | 2.54 |  | | |
| 33 | | | 601 | | | *sagC* | | | streptolysin S biosynthesis protein (C) SagC | | | 2.59 |  | | |
| 34 | | | 602 | | | *sagD* | | | streptolysin S biosynthesis protein (D) SagD | | | 2.86 |  | | |
| 35 | | | 603 | | | *sagE* | | | streptolysin S self-immunity protein SagE | | | 2.99 |  | | |
| 36 | | | 604 | | | *sagF* | | | streptolysin S export protein SagF | | | 3.12 |  | | |
| 37 | | | 605 | | | *sagG* | | | streptolysin S export protein SagG | | | 3.32 |  | | |
| 38 | | | 606 | | | *sagH* | | | streptolysin S export permease protein SagH | | | 3.46 |  | | |
| 39 | | | 607 | | | *sagI* | | | streptolysin S export permease protein SagI | | | 3.19 |  | | |
| 40 | | | 656 | | | *infC* | | | translation initiation factor (3) InfC | | | | 1.55 | | |
| 41 | | | 657 | | | *rpmL* | | | LSU ribosomal protein (L35p) RpmI | | | | 1.61 | | |
| 42 | | | 671 | | |  | | | hypothetical protein | | | | 1.52 | | |
| 43 | | | 685 | | | *rpsP* | | | SSU ribosomal protein (S16p) RpsP | | | 1.79 |  | | |
| 44 | | | 686 | | |  | | | RNA-binding protein | | | 1.59 | -1.52 | | |
| 45 | | | 689 | | | *czcD* | | | cobalt-zinc-cadmium resistance protein CzcD | | | | 1.64 | | |
| 46 | | | 697 | | | *fruK* | | | 1-phosphofructokinase protein FruK | | | 1.70 |  | | |
| 47 | | | 698 | | | *fruA* | | | PTS system fructose-specific IIA-IIB-IIC component FruA | | | 2.20 |  | | |
| 48 | | | 711 | | | *ushA* | | | extracellular 5'-nucleotidase UshA | | | 2.61 |  | | |
| 49 | | | 727 | | | *clpL* | | | ATP-dependent Clp proteinase protein ClpL | | | | 1.88 | | |
| 50 | | | 812 | | | *manX* | | | PTS system IIA component ManX | | | 2.40 |  | | |
| 51 | | | 813 | | |  | | | PTS system IIB component | | | 2.78 |  | | |
| 52 | | | 814 | | | *manY* | | | PTS system IIC component ManY | | | 2.91 |  | | |
| 53 | | | 815 | | | *manZ* | | | PTS system mannose/fructose family IID component ManZ | | | 2.76 |  | | |
| 54 | | | 856 | | | *aphA* | | | acid phosphatase/phosphotransferase protein AphA | | | 1.89 |  | | |
| 55 | | | 877 | | | *pbuX* | | | xanthine permease protein PbuX | | | | -1.51 | | |
| 56 | | | 878 | | | *apbE* | | | thiamin biosynthesis lipoprotein ApbE | | | | -1.63 | | |
| 57 | | | 910 | | | *oadA.*1 | | | Oxaloacetate decarboxylase alpha chain, OadA | | | 1.80 |  | | |
| 58 | | | 911 | | |  | | | hypothetical protein | | | 1.91 |  | | |
| 59 | | | 912 | | |  | | | Biotin carboxyl carrier protein of oxaloacetate decarboxylase | | | 1.87 |  | | |
| 60 | | | 913 | | | *oadB.*1 | | | Oxaloacetate decarboxylase beta chain, OadB | | | 1.69 |  | | |
| 61 | | | 916 | | | *citM* | | | Citrate complex transporter, CitM | | | 3.25 |  | | |
| 62 | | | 918 | | |  | | | Biotin carboxyl carrier protein of oxaloacetate decarboxylase | | | 2.13 |  | | |
| 63 | | | 919 | | | *oadB.*2 | | | Oxaloacetate decarboxylase beta chain, OadB | | | 1.58 |  | | |
| 64 | | | 920 | | |  | | | hypothetical protein | | | 1.89 |  | | |
| 65 | | | 921 | | | *citD* | | | Citrate lyase gamma chain, CitD | | | 2.94 |  | | |
| 66 | | | 922 | | | *citE* | | | Citrate lyase beta chain, CitE | | | 2.91 |  | | |
| 67 | | | 923 | | | *citF* | | | Citrate lyase alpha chain, CitF | | | 3.36 |  | | |
| 68 | | | 924 | | | *citX* | | | Apo-citrate lyase phosphoribosyl-dephospho-CoA transferase, CitX | | | 3.73 |  | | |
| 69 | | | 925 | | | *oadA.*2 | | | Oxaloacetate decarboxylase alpha chain, OadA | | | 5.28 |  | | |
| 70 | | | 942 | | | *fhs* | | | Formate--tetrahydrofolate ligase, Fhs | | | 1.83 |  | | |
| 71 | | | 943 | | | *lplA.*2 | | | Lipoate-protein ligase A, LplA | | | 2.18 |  | | |
| 72 | | | 944 | | |  | | | ATPase associated with chromosome architecture/replication | | | 2.19 |  | | |
| 73 | | | 945 | | |  | | | hypothetical protein | | | 2.08 |  | | |
| 74 | | | 946 | | | *gcvH* | | | Glycine cleavage system H protein, GcvH | | | 2.01 |  | | |
| 75 | | | 947 | | |  | | | Luciferase-like monooxygenase | | | 1.96 |  | | |
| 76 | | | 948 | | |  | | | NADH:flavin oxidoreductase | | | 1.78 |  | | |
| 77 | | | 949 | | | *lplA.*3 | | | Lipoate-protein ligase A, LplA | | | 1.58 |  | | |
| 78 | | | 959 | | | *rsmC* | | | 16S rRNA methyltransferase, RsmC | | | 1.73 |  | | |
| 79 | | | 985 | | |  | | | hypothetical protein | | | 2.09 |  | | |
| 80 | | | 986 | | |  | | | General stress response protein | | | 2.02 |  | | |
| 81 | | | 987 | | |  | | | General stress protein | | | 1.90 |  | | |
| 82 | | | 988 | | |  | | | hypothetical protein | | | 1.68 |  | | |
| 83 | | | 989 | | |  | | | hypothetical protein | | | 1.69 |  | | |
| 84 | | | 990 | | |  | | | hypothetical protein | | | 1.72 |  | | |
| 85 | | | 995 | | | *cfa* | | | CAMP factor, Cfa | | | 1.57 |  | | |
| 86 | | | 1000 | | | *glmS* | | | glucosamine--fructose-6-phosphate aminotransferase GlmS | | | | 1.69 | | |
| 87 | | | 1011 | | | *glgP* | | | maltodextrin phosphorylase protein GlgP | | | 5.05 |  | | |
| 88 | | | 1012 | | | *malQ* | | | 4-alpha-glucanotransferase (amylomaltase) protein MalQ | | | 3.56 |  | | |
| 89 | | | 1014 | | | *malE* | | | maltose/maltodextrin ABC transport system substrate-binding lipoprotein MalE | | | 4.26 |  | | |
| 90 | | | 1015 | | | *malF* | | | maltose/maltodextrin ABC transport system permease protein MalF | | | 3.43 |  | | |
| 91 | | | 1016 | | | *malG* | | | maltose/maltodextrin ABC transport system permease protein MalG | | | 3.92 |  | | |
| 92 | | | 1017 | | | *malH* | | | LacI family transcriptional regulatory protein MalH | | | 1.89 |  | | |
| 93 | | | 1018 | | | *malA* | | | maltodextrose utilization protein MalA | | | 1.84 |  | | |
| 94 | | | 1019 | | | *malD* | | | maltodextrin transport system permease protein MalD | | | 1.80 |  | | |
| 95 | | | 1020 | | | *malC* | | | maltodextrin transport system permease protein MalC | | | 1.96 |  | | |
| 96 | | | 1021 | | | *amyA* | | | cyclomaltodextrin glucanotransferase precursor AmyA | | | 1.83 |  | | |
| 97 | | | 1022 | | | *amyB* | | | cyclomaltodextrinase AmyB | | | 1.79 |  | | |
| 98 | | | 1023 | | | *malX* | | | maltose/maltodextrin-binding protein MalX | | | 1.52 |  | | |
| 99 | | | 1034 | | | *celB* | | | PTS system cellobiose specific IIC component CelB | | | 1.67 |  | | |
| 100 | | | 1035 | | |  | | | hypothetical protein | | | 1.79 |  | | |
| 101 | | | 1036 | | | *celC* | | | PTS system sugar cellobiose specific IIA component CelC | | | 1.64 |  | | |
| 102 | | | 1037 | | | *celA* | | | PTS system cellobiose-specific IIB component CelA | | | 1.57 |  | | |
| 103 | | | 1038 | | |  | | | Transcriptional antiterminator of lichenan operon, BglG family | | | 1.53 |  | | |
| 104 | | | 1039 | | |  | | | hypothetical protein | | | 1.63 |  | | |
| 105 | | | 1041 | | |  | | | PnuC-like ribosyl nicotinamide transporter | | | | 1.50 | | |
| 106 | | | 1048 | | |  | | | putative thioesterase | | | 1.70 |  | | |
| 107 | | | 1051 | | | *paaI* | | | phenylacetic acid degradation protein PaaI | | | | 1.50 | | |
| 108 | | | 1090 | | |  | | | major facilitator:oxalate:formate antiporter | | | 2.20 |  | | |
| 109 | | | 1167 | | | *arcC* | | | carbamate kinase protein ArcC | | | 10.08 |  | | |
| 110 | | | 1168 | | |  | | | Xaa-His dipeptidase protein PepV-like | | | 7.16 |  | | |
| 111 | | | 1169 | | | *arcD* | | | arginine/ornithine antiporter ArcD | | | 5.52 |  | | |
| 112 | | | 1170 | | | *arcB* | | | ornithine carbamoyltransferase protein ArcB | | | 4.35 |  | | |
| 113 | | | 1171 | | |  | | | acetyltransferase | | | 4.84 |  | | |
| 114 | | | 1172 | | | *arcA* | | | arginine deiminase protein ArcA | | | 3.92 |  | | |
| 115 | | | 1204 | | | *lacZ* | | | beta-galactosidase protein LacZ | | | 1.68 |  | | |
| 116 | | | 1205 | | | *trxR* | | | two component system response regulator TrxR | | | 1.52 |  | | |
| 117 | | | 1206 | | | *trxS* | | | two component system sensor kinase TrxS | | | 1.52 |  | | |
| 118 | | | 1207 | | |  | | | hypothetical protein | | | 1.52 |  | | |
| 119 | | | 1208 | | |  | | | sugar ABC transport system substrate-binding lipoprotein | | | 2.32 |  | | |
| 120 | | | 1209 | | |  | | | sugar ABC transport system permease | | | 2.24 |  | | |
| 121 | | | 1210 | | |  | | | sugar ABC transport system permease | | | 2.35 |  | | |
| 122 | | | 1211 | | |  | | | transcriptional regulator/sugar kinase | | | 2.26 |  | | |
| 123 | | | 1212 | | |  | | | hypothetical protein | | | 2.53 |  | | |
| 124 | | | 1213 | | | *bglB* | | | beta-glucosidase protein BglB | | | 2.44 |  | | |
| 125 | | | 1214 | | |  | | | beta-N-acetylglucosaminidase | | | 2.09 |  | | |
| 126 | | | 1215 | | |  | | | LacI-like tanscriptional regulator | | | 1.76 |  | | |
| 127 | | | 1217 | | |  | | | alpha-mannosidase | | | 1.83 |  | | |
| 128 | | | 1240 | | | *cysK/M* | | | cysteine synthase protein CysK/CysM | | | -2.09 |  | | |
| 129 | | | 1274 | | |  | | | ABC-type amino acid transport system permease component | | | -1.53 |  | | |
| 130 | | | 1288 | | | *tal* | | | transaldolaseprotein Tal | | | 2.12 |  | | |
| 131 | | | 1289 | | |  | | | putative transcriptional regulatory protein | | | 1.89 |  | | |
| 132 | | | 1308 | | | *lacD* | | | tagatose 1,6-diphosphate aldolase protein LacD | | | 1.77 |  | | |
| 133 | | | 1309 | | | *lacC* | | | tagatose-6-phosphate kinase/1-phosphofructokinase protein LacC | | | 1.80 |  | | |
| 134 | | | 1310 | | | *lacB* | | | galactose-6-phosphate isomerase protein LacB | | | 1.68 |  | | |
| 135 | | | 1311 | | | *lacA* | | | galactose-6-phosphate isomerase protein LacA | | | 1.57 |  | | |
| 136 | | | 1318 | | | *copA* | | | opper-translocating P-type ATPase protein CopA | | | | 1.51 | | |
| 137 | | | 1319 | | | *copY* | | | copper transport operon transcriptional repressor protein CopY | | | | 1.59 | | |
| 138 | | | 1345 | | | *accD* | | | acetyl-coenzyme A carboxyl transferase beta subunit AccD | | | | -1.65 | | |
| 139 | | | 1346 | | | *accC* | | | biotin carboxylase of acetyl-CoA carboxylase protein AccC | | | | -1.68 | | |
| 140 | | | 1347 | | | *fabZ* | | | beta-hydroxyacyl-acyl carrier protein FabZ | | | | -1.77 | | |
| 141 | | | 1348 | | | *accB* | | | biotin carboxyl carrier protein AccB | | | | -1.86 | | |
| 142 | | | 1349 | | | *fabF* | | | 3-oxoacyl-[acyl-carrier-protein] synthase protein FabF | | | | -1.84 | | |
| 143 | | | 1350 | | | *fabG* | | | 3-ketoacyl-(acyl-carrier-protein) reductase protein FabG | | | | -1.68 | | |
| 144 | | | 1351 | | | *fabD* | | | malonyl CoA-acyl carrier protein transacylase FabD | | | | -1.75 | | |
| 145 | | | 1352 | | | *fabK* | | | Enoyl-[acyl-carrier-protein] reductase FabK | | | | -1.69 | | |
| 146 | | | 1353 | | | *acpP* | | | acyl carrier protein AcpP | | | | -1.51 | | |
| 147 | | | 1356 | | | *phaB* | | | enoyl-CoA hydratase protein PhaB | | | | -1.54 | | |
| 148 | | | 1358 | | | *dnaK* | | | chaperone protein DnaK | | | | 1.81 | | |
| 149 | | | 1359 | | | *grpE* | | | heat shock protein GrpE | | | | 1.77 | | |
| 150 | | | 1360 | | | *hrcA* | | | heat-inducible transcription repressor HrcA | | | | 1.87 | | |
| 151 | | | 1377 | | |  | | | hypothetical protein | | | | 1.59 | | |
| 152 | | | 1386 | | | *htsC* | | | heme ABC transport system ATP-binding protein htsC | | | | 1.52 | | |
| 153 | | | 1389 | | | *shp* | | | heme binding protein Shp | | | | 1.60 | | |
| 154 | | | 1390 | | | *shr* | | | hemoprotein receptor Shr | | | | 1.70 | | |
| 155 | | | 1414 | | |  | | | hypothetical protein | | | | 1.74 | | |
| 156 | | | 1421 | | |  | | | hypothetical protein | | | | 1.67 | | |
| 157 | | | 1427 | | | *pfl* | | | pyruvate formate-lyase protein Pfl | | | 1.55 |  | | |
| 158 | | | 1432 | | |  | | | NorA-like major facillitator transporter | | | 4.96 |  | | |
| 159 | | | 1446 | | | *rpsN* | | | SSU ribosomal protein (S14p) RpsN | | | 1.68 |  | | |
| 160 | | | 1499 | | | *lacR* | | | lactose phosphotransferase system repressor protein LacR | | | | 1.50 | | |
| 161 | | | 1500 | | |  | | | hypothetical protein | | | | 1.61 | | |
| 162 | | | 1535 | | | *pulA* | | | pullulanase protein PulA | | | 4.66 |  | | |
| 163 | | | 1536 | | | *dexB* | | | glucan 1,6-alpha-glucosidase protein DexB | | | 4.08 |  | | |
| 164 | | | 1544 | | |  | | | endonuclease/exonuclease/phosphatase family protein | | | 3.74 |  | | |
| 165 | | | 1545 | | |  | | | PTS system maltose and glucose-specific fused IIABC component | | | 3.96 |  | | |
| 166 | | | 1546 | | | *rsmE* | | | ribosomal RNA SSU methyltransferase protein (E) RsmE | | | | 1.61 | | |
| 167 | | | 1564 | | |  | | | histidine triad protein | | | 1.81 |  | | |
| 168 | | | 1585 | | | *ropA* | | | peptidylproline cis-trans-isomerase protein PrsA; proteinase maturase RopA | | | -1.53 |  | | |
| 169 | | | 1591 | | | *sdaB* | | | streptodornase B protein SdaB | | | 2.33 |  | | |
| 170 | | | 1609 | | | *pepD* | | | dipeptidase protein PepD | | | 1.64 |  | | |
| 171 | | | 1610 | | |  | | | hypothetical protein | | | | 1.80 | | |
| 172 | | | 1611 | | |  | | | XRE-family ranscriptional regulator | | | | 1.64 | | |
| 173 | | | 1613 | | | *groES* | | | heat shock protein 60 family co-chaperone GroES | | | -1.50 | 1.61 | | |
| 174 | | | 1616 | | | *cspA* | | | cold shock protein CspA | | | 2.15 |  | | |
| 175 | | | 1629 | | |  | | | putative transcriptional regulator | | | 1.62 |  | | |
| 176 | | | 1633 | | | *treC* | | | trehalose-6-phosphate hydrolase protein treC | | | 2.06 |  | | |
| 177 | | | 1634 | | |  | | | PTS system trehalose-specific IIABC component | | | 1.88 |  | | |
| 178 | | | 1641 | | | *nrdG* | | | ribonucleotide reductase activating protein NrdG | | | | 1.56 | | |
| 179 | | | 1642 | | |  | | | acetyltransferase | | | | 1.58 | | |
| 180 | | | 1644 | | |  | | | hypothetical protein | | | | 1.75 | | |
| 181 | | | 1645 | | | *nrdD* | | | ribonucleotide reductase large subunit NrdD | | | | 1.77 | | |
| 182 | | | 1650 | | |  | | | ArsC family transcriptional regulator | | | -1.65 |  | | |
| 183 | | | 1658 | | |  | | | hypothetical protein | | | | 1.60 | | |
| 184 | | | 1659 | | | *argG* | | | arginine repressor protein ArgR | | | | 1.51 | | |
| 185 | | | 1669 | | | *cadD* | | | cadmium resistance protein CadD | | | | 1.85 | | |
| 186 | | | 1670 | | | *cadX* | | | cadmium efflux putative regulatory protein CadX | | | | 2.38 | | |
| 187 | | | 1671 | | |  | | | hypothetical protein | | | | 2.03 | | |
| 188 | | | 1672 | | |  | | | FtsK/SpoIIIE family protein | | | | 1.99 | | |
| 189 | | | 1673 | | |  | | | hypothetical protein | | | | 1.73 | | |
| 190 | | | 1674 | | |  | | | hypothetical protein | | | | 1.75 | | |
| 191 | | | 1675 | | |  | | | hypothetical protein | | | | 1.58 | | |
| 192 | | | 1677 | | |  | | | MutT/nudix family phosphohydrolase | | | | 1.82 | | |
| 193 | | | 1678 | | |  | | | PadR family transcriptional regulator | | | | 1.75 | | |
| 194 | | | 1682 | | |  | | | transcriptional regulator | | | | 1.60 | | |
| 195 | | | 1694 | | |  | | | putative transglycosylase | | | | -1.53 | | |

^a^ Locus tag designations are provided relative to clade 3 strain MGAS27061. There are no blocks of recombination that differ subclade 3D parental strain MGAS27556 for its isogenic LiaS (K214R) derivative.
